# Alteration of DNA supercoiling serves as a trigger of short-term cold shock repressed genes of *E. coli*

**DOI:** 10.1093/nar/gkac643

**Published:** 2022-08-03

**Authors:** Suchintak Dash, Cristina S D Palma, Ines S C Baptista, Bilena L B Almeida, Mohamed N M Bahrudeen, Vatsala Chauhan, Rahul Jagadeesan, Andre S Ribeiro

**Affiliations:** Laboratory of Biosystem Dynamics, Faculty of Medicine and Health Technology, Tampere University, Tampere 33520, Finland; Laboratory of Biosystem Dynamics, Faculty of Medicine and Health Technology, Tampere University, Tampere 33520, Finland; Laboratory of Biosystem Dynamics, Faculty of Medicine and Health Technology, Tampere University, Tampere 33520, Finland; Laboratory of Biosystem Dynamics, Faculty of Medicine and Health Technology, Tampere University, Tampere 33520, Finland; Laboratory of Biosystem Dynamics, Faculty of Medicine and Health Technology, Tampere University, Tampere 33520, Finland; Laboratory of Biosystem Dynamics, Faculty of Medicine and Health Technology, Tampere University, Tampere 33520, Finland; Laboratory of Biosystem Dynamics, Faculty of Medicine and Health Technology, Tampere University, Tampere 33520, Finland; Laboratory of Biosystem Dynamics, Faculty of Medicine and Health Technology, Tampere University, Tampere 33520, Finland; Center of Technology and Systems (CTS-Uninova), NOVA University of Lisbon 2829-516, Monte de Caparica, Portugal

## Abstract

Cold shock adaptability is a key survival skill of gut bacteria of warm-blooded animals. *Escherichia coli* cold shock responses are controlled by a complex multi-gene, timely-ordered transcriptional program. We investigated its underlying mechanisms. Having identified short-term, cold shock repressed genes, we show that their responsiveness is unrelated to their transcription factors or global regulators, while their single-cell protein numbers’ variability increases after cold shock. We hypothesized that some cold shock repressed genes could be triggered by high propensity for transcription locking due to changes in DNA supercoiling (likely due to DNA relaxation caused by an overall reduction in negative supercoiling). Concomitantly, we found that nearly half of cold shock repressed genes are also highly responsive to gyrase inhibition (albeit most genes responsive to gyrase inhibition are not cold shock responsive). Further, their response strengths to cold shock and gyrase inhibition correlate. Meanwhile, under cold shock, nucleoid density increases, and gyrases and nucleoid become more colocalized. Moreover, the cellular energy decreases, which may hinder positive supercoils resolution. Overall, we conclude that sensitivity to diminished negative supercoiling is a core feature of *E. coli*’s short-term, cold shock transcriptional program, and could be used to regulate the temperature sensitivity of synthetic circuits.

## INTRODUCTION

*Escherichia coli* is widely found in the gut of warm-blooded animals in all natural habitats. It usually propagates to new hosts when the original host excretes (or perishes) (1). For this, it becomes airborne until encountering new hosts. Thus, it will face (sometimes extreme) temperature downshifts. To cope with these, it has evolved a complex transcriptional program involving many genes (2,3). Their responses are likely subject to regulatory mechanisms yet to be decoded, which are responsible for the implementation of physiological changes that enhance the chances of survival.

As other prokaryotes under cold shock, *E. coli* halts cell division and undergoes an ‘acclimation phase’, during which changes occur at a multi-scale level, from heterogeneous changes in the kinetics of transcription (4,5) and translation (1,6–9), up to a decrease in membrane fluidity (10,11) and increase cytoplasmic viscosity (12,13).

Measurements of transcriptomes at non-optimal temperatures revealed broad responses by specific gene cohorts (14,15). During cold shock, a small gene cohort has a fast, transient response, another has a long-term response, while most other genes (including essential genes) remain stable (14). This diversity of single-gene responses may be explained by the likely existence of multiple causes for their alterations in expression rates during cold shock. For example, studies using synthetic gene constructs suggest that temperature can affect the kinetics of rate-limiting steps in transcription initiation, such as the closed and open complex formations (4), and such effects can differ between promoters (16). Other studies showed that temperature affects chromosomal DNA compaction (17–19), which is associated with supercoiling buildup (19,20). Changing supercoiling levels can cause genome-wide disturbances in gene expression (21–24). Other influences may be indirect, e.g. temperature affects energy-dependent events, such as interactions between nucleoid-associated proteins (NAPs), and chromosomal DNA (25), which affect DNA topology, and thus transcription kinetics (26–28).

Changes in DNA supercoiling may be a quick, efficient means to tune gene expression during stresses, including osmotic shifts (29), oxidative stress (30) and starvation (31). Many promoters of stress-inducible genes (such as virulence genes in pathogenic bacteria) are sensitive to changes in DNA supercoiling (32,33). Thus, it is possible that temperature-dependent changes in DNA superhelical density may be responsible for the responsiveness of some cold shock repressed genes.

In agreement, a recent study (16) tracked RNA production at the molecular level by synthetic variants of the Lac promoter. It was shown that, at low temperatures, RNA production kinetics is weaker and noisier when the gene is chromosome integrated than when it is plasmid borne (in plasmids, supercoiling buildup should be slower due to the annihilation of positive and negative supercoils (27)). They also showed the same phenomenon under gyrase and topoisomerase I inhibition, as well as in energy-depleted cells. Finally, by integrating data from (14) and (24) they hypothesized that cold shock repressed genes may exhibit atypical supercoiling sensitivity.

**Figure 1.**
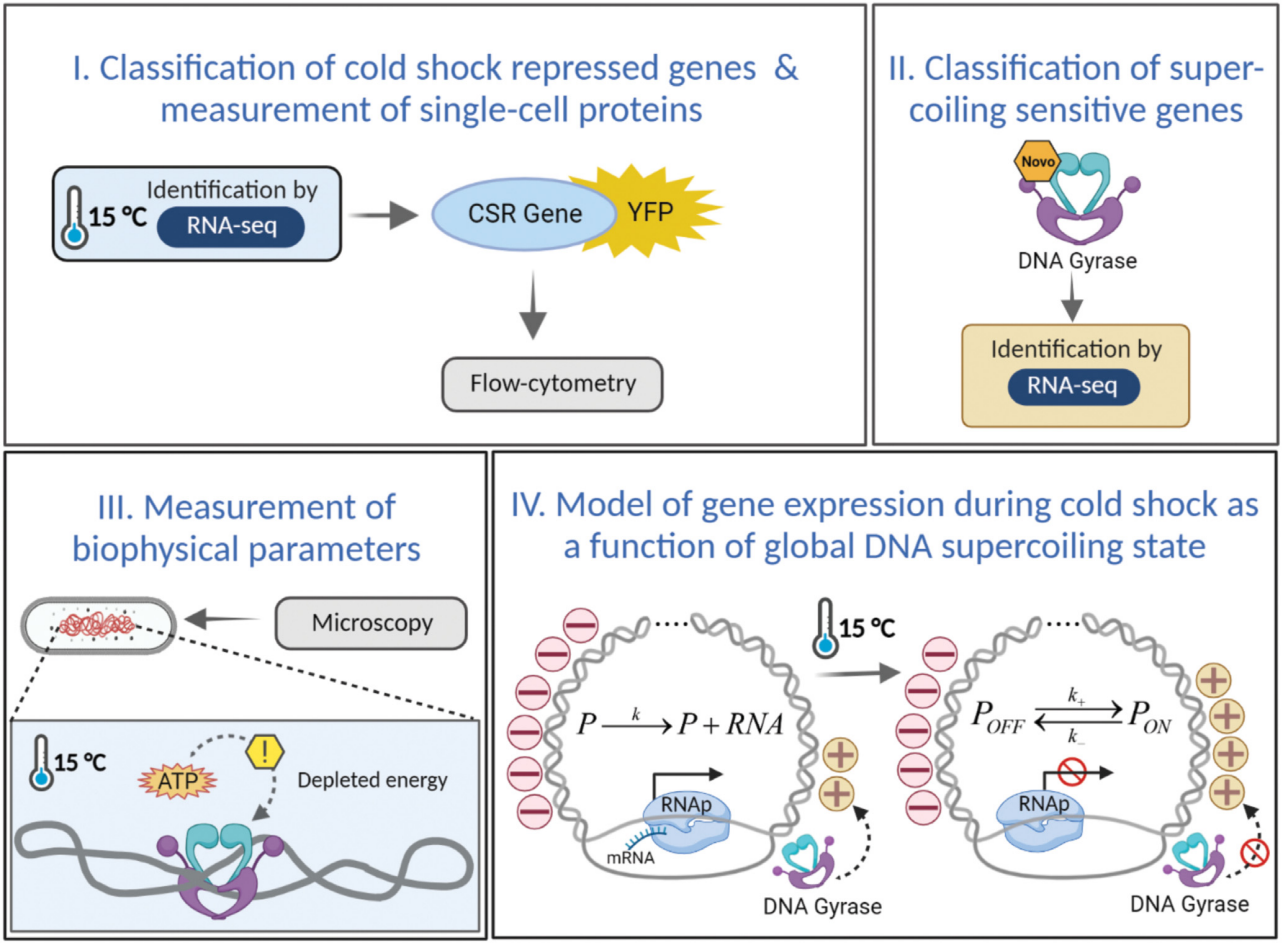
Workflow illustration. (**I**) Identification of short-term cold shock repressed (CSR) genes from RNA-seq in optimal and cold shock conditions. We also performed flow cytometry of protein levels of 30 cold shock repressed genes using a YFP fusion library (34). (**II**) Identification of strongly supercoiling sensitive genes by RNA-seq following gyrase inhibition by novobiocin, followed by an assessment of the correlation between the genes’ responses to both novobiocin and cold shock. (**III**) Measurements of biophysical parameters to estimate cell energy (ATP), morphology, and the engagement of gyrase and RNAP with the nucleoid. (**IV**) Schematic illustration of cold shock repressed genes behaviour and corresponding kinetic model in optimal and cold shock conditions. In optimal conditions the global state of the DNA is negatively supercoiled (20,115). During cold shock, the promoters’ locking propensity increases, due to DNA relaxation (i.e. reduced overall negative DNA supercoiling), likely caused by reduced topoisomerases’ efficiency, particularly gyrase. The signs ‘−’ and ‘+’ represent local, negative and positive supercoiling, respectively. Created with BioRender.com.

Here, we subjected *E. coli* cells to cold shock, identified cold shock repressed genes by RNA-seq and investigated their common features (Figure 1, step I). Also, we measured the corresponding single-cell protein expression dynamics of 30 genes identified as cold shock repressed (Figure 1, step I). From the single-cell gene expression data, we hypothesized potential regulatory mechanisms that could explain the cold shock repressed genes dynamics of response to cold shock. Based on those hypotheses, we performed RNA-seq following gyrase inhibition, to identify supercoiling sensitive genes (Figure 1, step II). Combining the RNA-seq data, we then identified which genes are both cold shock repressed as well as strongly supercoiling sensitive. We then investigated whether the cellular and nucleoid morphology, along with the cell energy levels during cold shock support the hypothesis that high supercoiling sensitivity provides some cold shock repressed genes with their enhanced short-term response to cold shock (Figure 1, step III). Finally, we proposed models that account realistically for the short-term response dynamics of cold shock repressed genes due to high supercoiling sensitivity (Figure 1, step IV). In the end, we discuss potential applications of this underlying mechanism of being repressed during cold shock. Finally, all abbreviations and symbols used in this study are listed in Supplementary Table S1.

## MATERIALS AND METHODS

### Bacterial strains, growth conditions, and gene expression measurements

We used *E. coli* K-12 MG1655 for RNA and protein measurements, since it is the control strain of the YFP fusion library (Supplementary Table S2) (34). From a glycerol stock (at −80°C), cells were streaked on LB agar plates and incubated at 37°C overnight. The next day, a single colony was picked from the plate, inoculated in fresh LB medium supplemented with antibiotics (34 μg/ml chloramphenicol for YFP tagged strains) and incubated at 30°C overnight with shaking at 250 RPM. Overnight culture cells were then diluted into fresh M9 media, supplemented with 0.4% glucose, amino acids, and vitamin solutions, until reaching 0.03 OD_600_ (optical density at 600 nm measured by Ultrospec 10, Amersham biosciences, UK) and allowed to grow at 30°C with aeration until reaching the mid-exponential phase of growth (OD_600_ of 0.3). At this moment, the temperature was downshifted (Innova^®^ 40 incubator, New Brunswick Scientific, USA) and cells were incubated for another 180 min. Cold shock conditions are imposed by placing cells at 10–15°C (14). Culture temperatures were monitored using a thermometer.

For measurements under gyrase inhibition, we added the antibiotic novobiocin (50 μg/ml) when cells reached an OD_600_ ∼0.3. We do not expect this concentration to affect the cell division rate. Specifically, the cells contain the acrA gene (35), whose main function is to protect against hydrophobic inhibitors, such as novobiocin, by pumping them out of the cell (35,36). In agreement, the growth rate relative to the control only decreased for 200 μg/ml or higher concentrations of novobiocin (Supplementary Figure S1)

To measure RpoS, we used a MG*mCherry* (*rpoS*:mCherry) strain (kind gift from James Locke (37)), where the *rpoS* gene codes for σ^38^, which is endogenously tagged with mCherry.

For intracellular ATP measurements, we used the QUEEN 2m, a kind gift from Hiromi Imamura (38) (Supplementary Table S2 for details). For measurements under energy depletion, we added 100 μM 2,4-dinitrophenol (DNP) when cells reached an OD_600_ ∼0.3, without affecting the growth.

We measured RNA and protein expression levels by RNA-seq (Supplementary Section I) and by flow cytometry (Supplementary Section II), respectively. We used pulse width data from flow-cytometry as a proxy for cell size (39–41), required to estimate protein concentrations. We verified these results using microscopy data and image analysis (Supplementary Section III).

### Nucleoid visualization by DAPI

To study the effect of cold shock on nucleoid size, cells were fixed with 3.7% formaldehyde in phosphate-buffered saline (PBS, pH 7.4) for 30 min at room temperature, followed by washing with PBS to remove excess formaldehyde. The pellets were suspended in PBS, and DAPI (4′,6-diamidino-2-phenylindole) (2 μg/ml) was added to the suspension to stain the nucleoid. After incubating for 20 min in the dark, cells were centrifuged and washed twice with PBS to remove excess DAPI. Cells were then re-suspended in PBS and 3 μl of these cells were placed on a 1% agarose gel pad for microscopy (42). Segmentation of nucleoids to extract their size and location in the cells from microscopy images is described Supplementary Section III.

### Cellular ATP levels

QUEEN-2m cells (Supplementary Table S2) were grown as described in Methods Section *Bacterial strains, growth conditions, and gene expression measurements*. We tracked ATP levels (Supplementary Figure S2) using a Biotek Synergy HTX Multi-Mode Reader. The solution was excited at 400 nm and emission was recorded at 513 nm. Similarly, the solution was re-excited at 494 nm and emission was recorded at 513 nm. The ratio of 513 nm emission intensity at these two excitation wavelengths, denoted as ‘400ex/494ex’, is used as a proxy for cellular ATP levels as proposed in (38).

### Stochastic model of cold shock response

We used stochastic simulations to estimate the expected noise in gene expression (as measured by the squared coefficient of variation, CV^2^, of gene expression levels in individual cells), assuming the models described in Section *An ON-OFF model can explain the short-term dynamics of cold shock repressed genes*. Simulations were performed using SGNSim (43), whose dynamics follows the Stochastic Simulation Algorithm (44,45). The time length of each simulation was set to 10^6^ s and the results for each model were collected from 100 independent runs, which sufficed to obtain consistent results (46). Finally, at the start of each run, in addition to the parameter values in Supplementary Table S3, it was set that there is one promoter in the system. The promoter was initially in the ‘ON’ state.

### Information from RegulonDB

Our data on transcription factor (TF) interactions (v10.5), operon organization (v10.9) and nucleotide sequence (v10.9) was extracted from RegulonDB (47).

### Microscopy image analysis

Cells and nucleoids were identified and characterized by automatic segmentation and alignment of microscopy images using the software CellAging (48) and SCIP (49). For details, see Supplementary Section III. For examples of segmentations, see Supplementary Figure S3.

## RESULTS

### Cell morphology, physiology and master transcription regulators during cold shock

**Figure 2.**
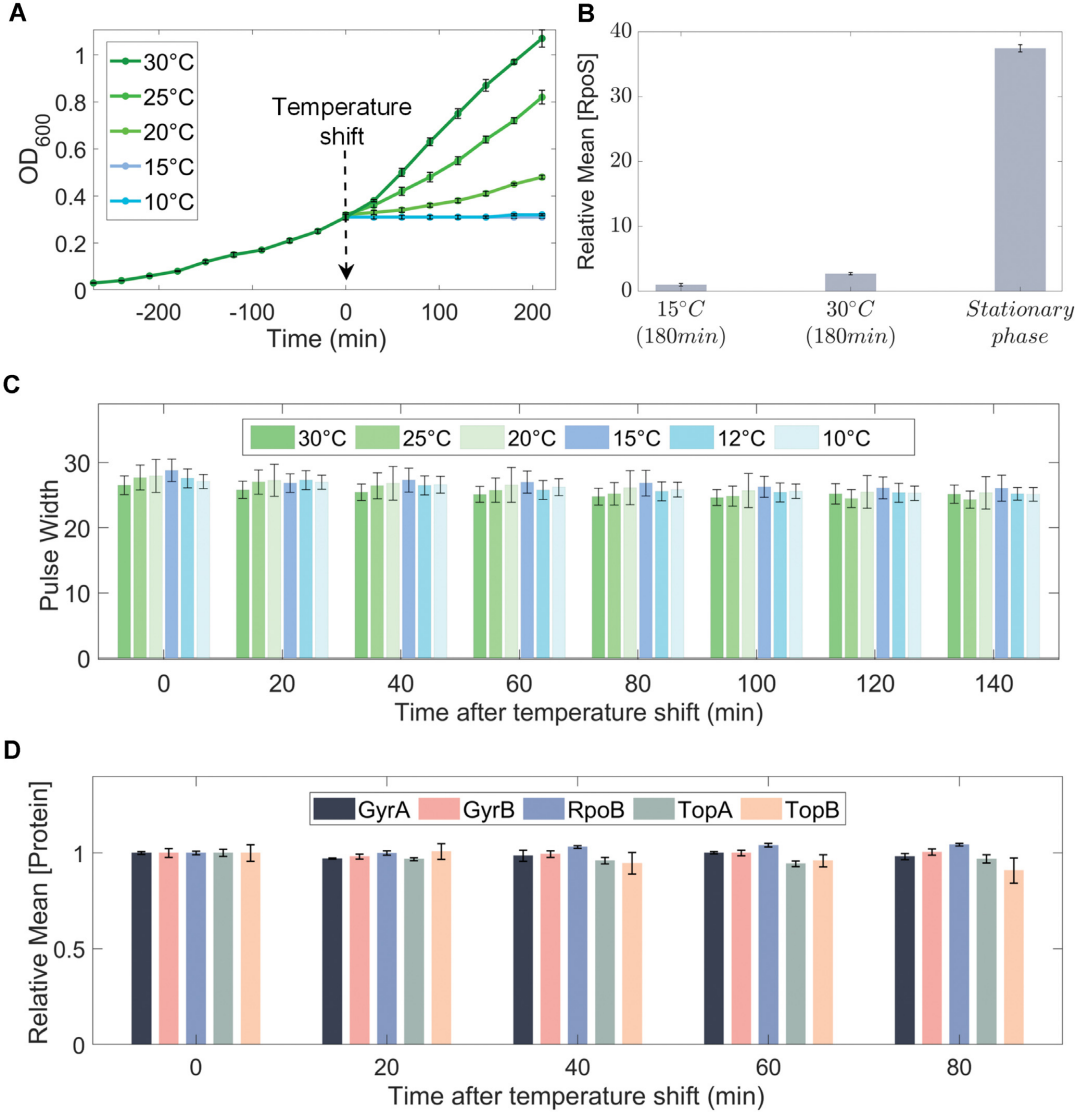
Effects of temperature shifts on cellular morphology, physiology, and global transcriptional regulators. (**A**) Growth curves at 10°C, 15°C, 20°C, 25°C and 30°C following a temperature shift, set to be minute 0. (**B**) Mean RpoS concentration during cold shock (15°C) and optimal conditions after 180 min, and during stationary growth (i.e. after 700 min). (**C**) Pulse width over time following temperature shifts (Methods Section *Bacterial strains, growth conditions, and gene expression measurements*). (**D**) Mean concentration of GyrA, GyrB, TopA, TopB and RpoB proteins over time after shifting temperature to 15°C. The vertical error bars are the standard error of the mean (SEM) from three biological repeats.

Having subject cells to cold shock (Methods Section *Bacterial strains, growth conditions, and gene expression measurements*), we first studied physiological and morphological effects. Once at 15°C or lower temperatures, cells no longer divided (Figure 2A). Nevertheless, these cells are not likely to be shifting to stationary growth, since RpoS concentrations remain low (Supplementary Figure S4) (50,51) (Methods Section *Bacterial strains, growth conditions, and gene expression measurements*), when compared to cells in optimal conditions and to cells in the stationary growth phase (Figure 2B). Meanwhile, their size was not affected, according to microscopy (Supplementary Figure S5) and flow cytometry (Figure 2C and Supplementary Section II) data.

Next, we examined potential short-term effects of cold shock on the concentrations of the master regulators of transcription, since if they change, it could influence the dynamics of cold shock repressed genes (not to be confused with global TF regulators in Supplementary Table S4). In detail, we observed RNA polymerase (RNAP) by tracking a YFP tagged β subunit, which is the product of the rpoB gene (Supplementary Table S2). We also observed the two subunits of gyrase (GyrA and GyrB) and topoisomerases I and III (TopA and TopB, respectively) using a YFP fusion library (34), since they respond to (52,53) and are the master regulators of DNA supercoiling levels (54,55). As such, they heterogeneously influence transcription at a genome-wide level. Further, evidence suggests that the efficiency of gyrase and other topoisomerases is temperature sensitive (16,31,56).

Neither of these master regulators showed concentration changes during 80 min after cold shock (Figure 2D), while the RNA-seq measurements reported below (Section *Identification of short-term cold shock repressed genes*) to identify cold shock repressed genes were performed 20 min after cold shock. As such, short-term cold shock responsiveness, is not expected to be activated by changes in the concentrations of these master transcription regulators.

### Identification of short-term cold shock repressed genes

We performed RNA-seq measurements (Supplementary Section I) at 0, 20, 80 and 180 min after shifting temperature to 15°C and under optimal (control) temperature (Methods Section *Bacterial strains, growth conditions, and gene expression measurements*).

We classified single-gene responses to cold shock as ‘short-term’ when they occur *prior to* influence from direct input TFs (including global TF regulators). As such, based on cell doubling times (Figure 2A) and known rates of transcription and translation in *E. coli* (see e.g. (34,57)) we expect changes in RNA numbers 20 min after cold shock to be short-term, while subsequent changes at 80 and 180 min are here classified as being mid-term and long-term changes, respectively. Thus, to identify short-term cold shock repressed genes, we obtained the RNA log_2_ fold changes (LFC_CS_) at 20 min after shifting to cold shock. We also obtained control LFCs (LFC_CTRL_) after the same time interval when not shifting temperature. In both, the LFCs were calculated relative to RNA levels right before applying cold shock (here named the ‘0 min’ condition).

We classified a gene as ‘cold shock repressed’ when its LFC_CS_ <0 (with *P*-value < 0.05), provided that its corresponding LFC_CTRL_ ≥0 (with *P*-value < 0.05), since this enhances the chance that the repression at cold shock was due to the cold shock. We found that 381 genes (Supplementary File X2) respected these conditions. Meanwhile, the YFP fusion library (34) allows measuring the protein levels of 124 of them. From these 124, we selected genes that: (i) have high expression under optimal conditions reported in (34) (to enhance the changes for fluorescence values higher than the cell background fluorescence) and; (ii) LFC_CS_ <−0.23, i.e. their RNA levels were reduced by 15% or more, relative to the same RNA in the control condition, to ensure significant downregulation during cold shock at the protein level. We found that 30 of the 124 genes respected these conditions. Thus, we selected them for single-cell fluorescence measurements in the control and cold shock conditions. Finally, we selected 6 of these 30 genes and additionally collected single-cell, time-lapse flow cytometry data on their dynamics. Taken together, their expression levels cover the state space of protein expression levels of the 30 cold shock repressed genes. This filtering process is illustrated in Supplementary Figure S6.

### Ontology and evolutionary fitness of short-term cold shock repressed genes

We investigated the ontology (58,59) of cold shock repressed genes to identify the most affected biological processes. From an over-representation test (Supplementary Section IV), we compared the number of cold shock repressed genes related to specific biological processes (quantified by the fold enrichment) with the *expected* number, given genome-wide numbers.

The significantly over-represented biological processes are listed in Supplementary Table S5. Visibly, of 30 major biological processes in *E. coli* considered in gene ontology studies (58,59), cold shock repressed genes are mainly associated with metabolism and response to external stimulus (Supplementary Figure S7, Supplementary File X3). This agrees with reports that genes involved in metabolism are commonly affected during cold shock, which reduces growth rate and the rate of glycolysis (3,60,61).

**Figure 3.**
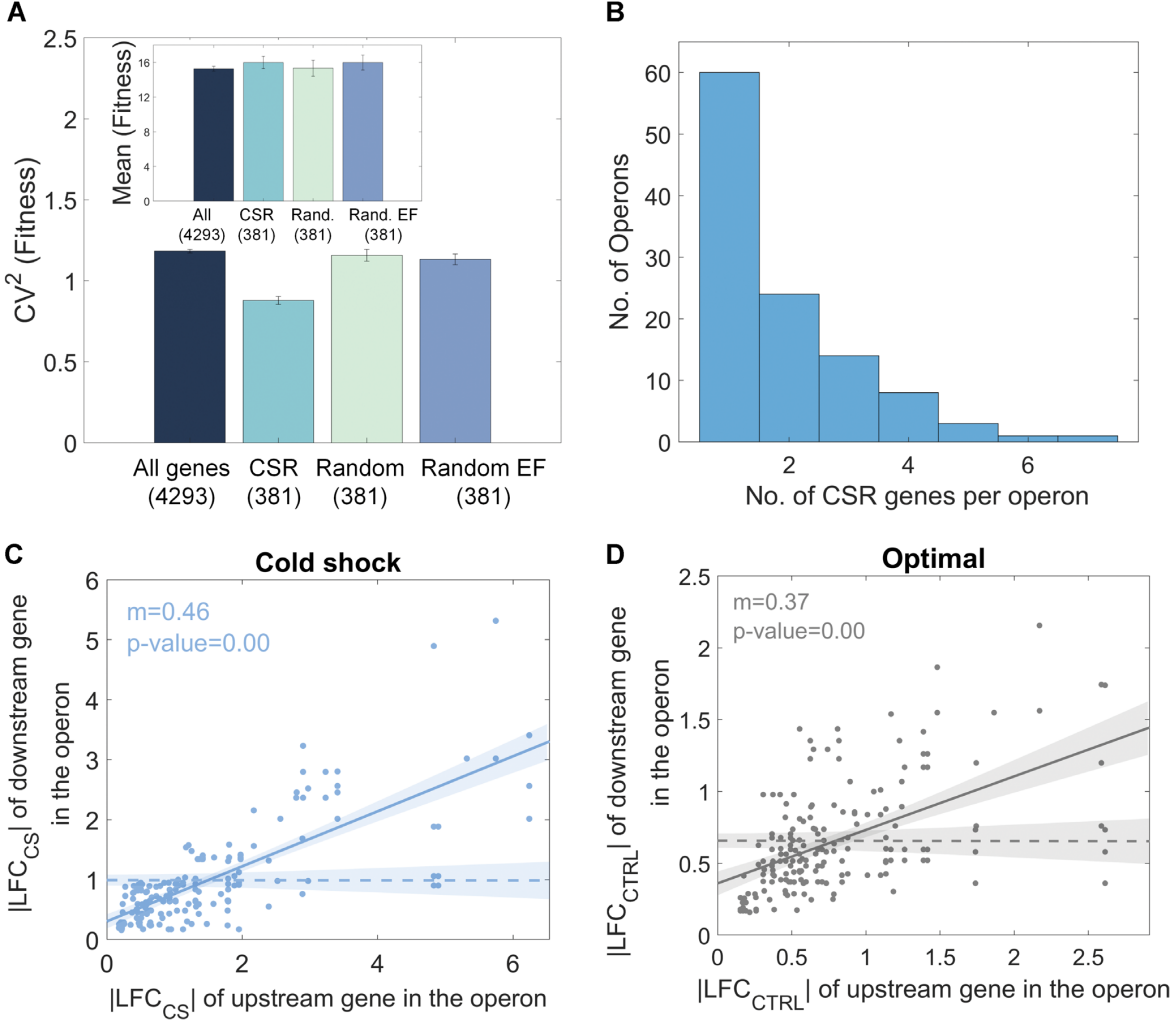
Characterization of cold shock repressed (CSR) genes. (**A**) Bar plot of the variability, CV^2^, of the fitness of all genes of *E. coli*’s genome (‘All genes’, dark blue bar), cold shock repressed cohort (‘CSR’, light blue bar), randomly selected cohort (‘Random’, light green bar) and a randomly selected cohort with the same size and same biological function (‘Random EF’, purple bar), where EF stands for ‘equal function’. The inset shows the mean fitness (in %) for each cohort. (**B**) Distribution of cold shock repressed (CSR) genes in operons. (**C**) Scatter plot between the |LFC_CS_| of pairs of cold shock repressed genes downstream and upstream in the same operon during cold shock. (**D**) |LFC_CTRL_| of cold shock repressed genes downstream in the operon plotted against the |LFC_CTRL_| of cold shock repressed genes upstream in the same operon at optimal temperatures. Dashed lines are the null models (Supplementary Section XIX). We performed an ANCOVA test for the null hypothesis that the line and the dashed line are not statistical distinguishable. *P*-value <0.05 rejects the null hypothesis.

Next, we studied the evolutionary fitness of the repressed genes (Supplementary Section V). Interestingly, while their average fitness is the same as expected by chance, their fitness variability is smaller than in same-sized cohorts of randomly selected genes (Figure 3A). This is not because they are over-represented in two functional groups, since the fitness variability of random cohorts with the same distribution of gene functions (164 genes related to metabolism, 41 genes responsive to stimulus, 36 genes in both groups, and 140 with other functions) also have statistically distinguishable fitness variability from cold shock repressed genes. Given that the fitness is positively correlated to the evolutionary conservation (Supplementary Section V), we hypothesize that their evolutionary ages are likely to be more similar than expected by chance as is the fitness.

### Short-term responses of cold shock repressed genes cannot be explained by transcription factor interactions, AT richness or closely spaced promoters

We investigated the potential influences on cold shock responsiveness from TFs, promoter AT richness, and closely spaced promoters. However, we failed to find any relationships.

First, we studied if the RNA-seq data on genes’ LFC responses to cold shock (Supplementary Section I) could be explained by the LFCs of their input TFs. Regulon DB (47) informs on 4435 TF interactions between the 4328 genes reported in our RNA-seq data. Of these, 733 TF interactions have, as output, a cold shock repressed gene (Supplementary File X2). We searched for correlations between the short-term |LFC| of cold shock repressed and non- cold shock repressed genes coding for a TF (input) and the |LFC_CS_| (mid- and long-term) of output genes of the TF. These time lags between the short-term and the mid- and long-term measurements (60 and 160 min, respectively) should suffice to account for the average time taken by TF proteins to be translated, assembled, matured, and/or degraded (62–64), following the increase or decrease in the numbers of RNAs that code them.

From (Supplementary Figure S8 and Supplementary Table S6), neither the mid- nor the long-term responses of cold shock repressed genes (at 80 and 180 min after cold shock, respectively) correlate with the short-term changes (at 20 min after cold shock) in their input TFs. Even when considering only global TF regulators (Supplementary Table S4), we did not find correlations (Supplementary Figure S9 and Supplementary Table S7). Nevertheless, there is evidence of information propagation in the TF network during cold shock (grey line in Supplementary Figure S8). On average, changes in non- cold shock repressed genes correlated with changes in their input TFs in cold shock and optimal temperatures (Supplementary Figures S8A and B and *P*-values in Supplementary Table S8). As such, the lack of correlation is characteristic of cold shock repressed genes.

Second, we considered that AT-rich promoters are more strongly expressed than GC-rich promoters in optimal conditions (65). Meanwhile, genes here classified as cold shock repressed are (necessarily) strongly expressing in optimal conditions (verified in Supplementary Figure S10A, and in agreement with (34)). Thus, potentially, cold shock repressed genes could have AT-rich promoters. We confronted the levels of AT richness (Supplementary Section VI) of promoter sequences with their short-term responses to cold shock (Supplementary Figure S10B). While there is a genome-wide correlation, when considering only cold shock repressed genes we do not find a correlation (Supplementary Figure S10B inset). Thus, AT richness is not likely involved in short-term responsiveness to cold shock.

Finally, we considered closely spaced promoters (reported in RegulonDB (47)). For convergent and divergent promoters, we searched for pairs of promoters in opposite strands, separated by <1500 nucleotides and without other transcription start sites, terminator sequences, or genes in between their transcription start sites. In tandem promoters, they are searched for using the same conditions, but imposing that they are located in the same DNA strand. Of the 4328 genes in the RNA-seq data following cold shock, 285 are controlled by two closely spaced promoters, based on these conditions. Of these, only 34 genes are cold shock repressed (by random chance it would be 24). Again, this is not statistically significant (Fisher test *P*-value > 0.05).

### Short-term responses of cold shock repressed genes can be partially explained by operon organization and by (p)ppGpp sensitivity

Genes in the same operon commonly exhibit co-expression (66,67). Meanwhile, of the 381 cold shock repressed genes, 169 are not in operon structures (according to RegulonDB (47)), while the remaining 212 are organized in a total of 111 operons (Figure 3B). We found that the |LFC_CS_| of pairs of cold shock repressed genes in the same operon are correlated, both in optimal conditions as well as in cold shock (Figures 3C and D).

To determine if this correlation is due to being in the same operon, we defined a null model with the same distribution of numbers of cold shock repressed genes per operon as in *E. coli* (Figure 3B), but whose genes forming each ‘pair’ are randomly selected. The random pairs show no correlation in |LFC| values (Figures 3C and D). Thus, the operons’ organization affects which genes are cold shock repressed, i.e. some are cold shock repressed because they are located downstream to a cold shock repressed gene in the same operon.

Nevertheless, there are 60 operons with only one cold shock repressed gene (Figure 3B). Thus, for a gene to be cold shock repressed, it does not suffice to be downstream from a cold shock repressed gene in an operon.

Finally, given a report that (p)ppGpp levels change and assist in cellular adaptation during cold shock (68), we considered the potential role of (p)ppGpp sensitivity during cold shock. Out of 1224 genes reported to be (p)ppGpp sensitive (1161 are present in our RNA-seq data) (69) and of 381 genes reported here to be cold shock repressed, we found that 147 genes combine both features. This is higher than expected by random chance. Specifically, the odds of a cold shock repressed gene to be (p)ppGpp sensitive is 1.82 (Fisher's exact test). Therefore, cold shock repressed genes have more chances of being (p)ppGpp sensitive than non- cold shock repressed genes. Nevertheless, the relatively small number of (p)ppGpp sensitive genes that are also cold shock repressed (compared to 381) confirms that this feature does not suffice for a gene to be cold shock repressed. The numbers of genes with the above features, along with the numbers of genes accumulating more than one feature are shown in the Venn diagram in Supplementary Figure S11A.

### The scaling between noise and mean of single-cell cold shock repressed protein numbers is temperature dependent

Given that the short-term response of cold shock repressed genes was uncorrelated with their input TFs dynamics, it is more likely that individual gene features were responsible for their repression during cold shock. We expect that, by repressing gene expression at the transcription level, these temperature-dependent mechanisms will affect how noise and mean expression correlate (34,70,71). To investigate this, we studied the single-cell distributions in protein numbers of 30 cold shock repressed genes (Methods Section *Bacterial strains, growth conditions, and gene expression measurements*, Supplementary Table S2, Supplementary File X1).

To quantify single-cell protein numbers, we first corrected the statistical moments of the distributions to account for cell auto-fluorescence (Supplementary Section VII). Then, we plotted the mean expression levels in optimal conditions against the corresponding protein numbers reported in (34) (Supplementary Figure S12). Given the best fitting line, from here onwards we convert protein expression levels into protein numbers using a scaling factor of 0.1.

Meanwhile, we did not find correlations between protein levels and cell size (Supplementary Figure S13A), in agreement with the lack of change in cell size with cold shock (Figure 2C and Supplementary Figure S5) implying that cell size is not affecting expression levels. Further, as expected from the mechanical coupling between transcription and translation in *E. coli* (72), the changes with cold shock in these 30 protein numbers correlated to the changes in the corresponding RNA numbers (Supplementary Figure S13B, Supplementary Section VIII), indicating that protein levels can be used to study the effects of regulatory mechanisms of transcription.

Finally, the robustness of the single-cell expression levels measured using the YFP-fusion library (34) was assessed. For this, we performed measurements of the expression levels of the same promoters using a promoter-fusion library instead (73). We find mean expression levels to be linearly correlated, with *R*^2^ values >0.74, at either temperature. We conclude that the measurements using the YFP-fusion library are robust for this cohort of genes under these conditions (Supplementary Figure S14, Supplementary Section XIII).

We plotted the mean single-cell protein numbers of cold shock repressed genes, *M*, against the corresponding noise (i.e. single-cell variability), as quantified by CV^2^, for each gene. Then, we best fitted the data by ordinary least squares (OLS) with the function (34,74):

(1) $$\begin{equation*}{C}{V}^{2} = \Omega {{/M}}\end{equation*}$$

Here, *Ω* is a constant while *M* is the mean number of proteins (estimated in Supplementary Figure S12). In general, *Ω* quantifies a signal-to-noise ratio, between the signal power, i.e. strength (here, the mean single-cell protein production rate over the degradation rate) and the power of the noise of the signal (here, the cell-to-cell variability in protein numbers, quantified by CV^2^). Equation (1) fits well the genome-wide single-cell protein numbers of *E. coli* in optimal growth conditions (34). Meanwhile, we hypothesized that *Ω* would differ, following cold shock. Specifically, if gene expression is noisier in cold shock, *Ω* should be higher. Further, that difference should depend on the mechanism causing the repression of cold shock repressed genes.

**Figure 4.**
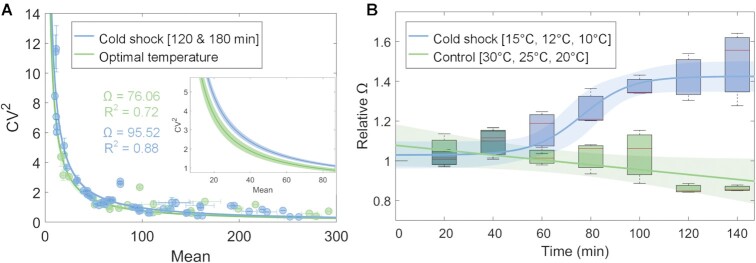
Relationship between CV^2^ and mean protein numbers over time, at different temperatures. Blue corresponds to cold shock conditions, while green corresponds to optimal conditions. (**A**) Squared coefficient of variation (CV^2^) versus mean protein numbers of 30 cold shock repressed genes (Supplementary file X1). Data at 120 and 180 min was merged as they did not differ (Supplementary Figure S15). We performed a 2-sample *t*-test to test the null hypothesis that *Ω* at 30°C and 15°C does not differ. The test rejected the null hypothesis (*P*-value of 0.02). (**B**) Box plot of relative *Ω* over time (set to 1 at *t* = 0 min) at ‘control’ and ‘cold shock’ temperatures (Supplementary File X1). The red line in the box is the median. The distance between the bottom and top of each box is the interquartile range. The vertical black bars are the range between the minimum and maximum value at each moment. For control and cold shock temperatures, we fit the best fitting function. An *F*-test on the regression model failed to reject the null hypothesis that the first order polynomial does not significantly improve the fitting compared to a 0-order polynomial (*P*-value = 0.06). The lines are the best-fit functions that maximize *R*^2^.

From Figure 4A, the inverse proportionality between CV^2^ and *M*, previously observed in optimal conditions (34,74,75), is valid during cold shock. However, CV^2^ is higher for the same *M* (*Ω* ∼26% higher than in optimal conditions). Meanwhile, since *Ω* does not change from 120 to 180 min after the cold shock, the changes likely occurred prior to 120 min following cold shock (Supplementary Figure S15).

To further investigate how *Ω* changed following cold shock, we measured single-cell distributions of protein levels of six genes each 20 min for 140 min following the temperature shifts. These genes (aldA, feoA, manY, ndk, pepN, tktB) have mean protein levels that cover the state space of *M* of the 30 cold shock repressed genes. For each time moment, we extracted the corresponding *Ω* that best fits the data (Figure 4B, Supplementary Figure S16). Visibly, *Ω* increases with time during cold shock, but not at optimal temperatures (Supplementary Figure S17).

Namely, at *T* ≤15°C, 40 min after cold shock, there is a sharp increase in *Ω*, while at *T* >15°C, *Ω* remains constant. In detail, for cold shock temperatures (10°C, 12°C and 15°C), the data is best fit by a sigmoid curve (*R*^2^ = 0.96) of the type $\frac{L}{{1 + {e}^{ - a( {x - {x}_0} )}}}$, where *L* is the curve's maximum value, *x*_0_ is the sigmoid midpoint and *a* is the steepness of the curve, which was set to 0.1 in order to maximize the *R*^2^ (we also attempted to fit polynomials, but none fitted better). Meanwhile, for the set of control temperatures, we fitted the data with a first order polynomial. The fitting of the first order polynomial did not significantly improve in comparison with the zero-order polynomial fitting (*P*-value of 0.06).

Overall, we suggest that, as cold shock is applied, a step emerges in transcription that is responsible for the strong repression of cold shock repressed genes, which not only reduces expression levels of cold shock repressed genes, but it also increases the scaling factor between noise and mean of protein numbers.

Finally, from (34), most protein number distributions in optimal conditions are well described by a Γ distribution. Given this, Equation (1) is valid, and the skewness (*S*) can also be written as a function of *M* (derivation in Supplementary Section XIV.a) as follows:

(2) $$\begin{equation*}S = \frac{2}{{\sqrt M }} \cdot \sqrt \Omega \end{equation*}$$

**Figure 5.**
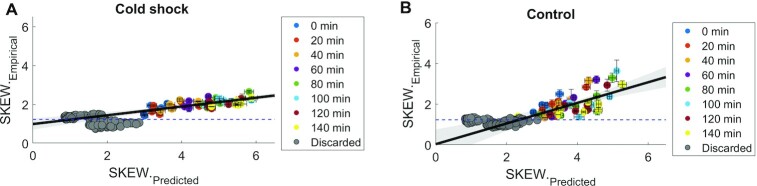
Correlation between empirical and predicted skewness (SKEW). (**A**) Cold shock temperatures (15°C, 12°C and 10°C). Skewness is predicted using Equation (2) and the empirical values of *Ω* (Section *Short-term responses of cold shock repressed genes can be partially explained by operon organization and by (p)ppGpp sensitivity*). (**B**) Control temperatures (30°C, 25°C and 20°C). Meanwhile, empirical data on skewness is extracted from single-cell distributions obtained by flow cytometry (Supplementary File X1) after being corrected for background noise. Blue dashed line is the estimated lower bound (Supplementary Section XV). Grey circles are data points excluded from the fitting due to being below or crossing the noise floor.

Given the *Ω* values above, we estimated the skewness using Equation (2) and compared to the empirical skewness values in cold shock and control conditions (Figure 5A and B). We find that the two correlate linearly (see also Supplementary Figure S18), above the noise floor, which was estimated using the data in Figure 4A (Supplementary Figure S19, Supplementary Section XV, Supplementary Table S9). This suggests that the effects of cold shock propagate up to the third moment of the single-cell distribution of protein numbers.

### An ON–OFF model can explain the short-term dynamics of cold shock repressed genes

**Figure 6.**
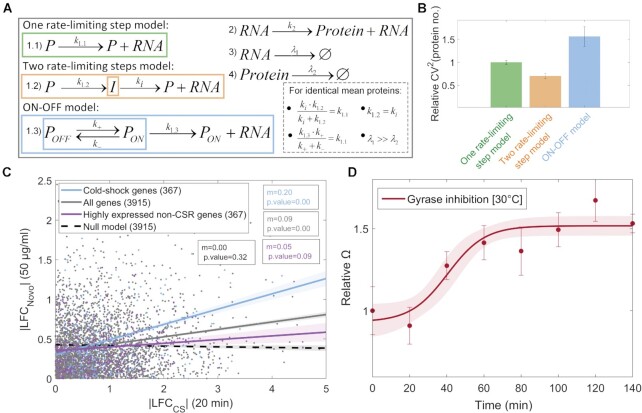
Nature of the short-term cold shock responses. (**A**) The three models considered differ in transcription (reaction (1.1) for the one rate-limiting step model, reactions (1.2) for the two rate-limiting steps model, and reactions (1.3) for the ON–OFF model), while having the same reactions for translation and RNA and protein decay (reactions (2), (3) and (4), respectively). The inset shows the conditions that the rate constants must respect to impose identical mean protein numbers for each model. (**B**) CV^2^ of protein numbers (relative to the one step model) from *in silico* predictions, assuming the parameter values in Supplementary Table S3. Vertical error bars are the SEM. (**C**) Scatter plots of |LFC_NOVO_| after adding novobiocin (relative to a control condition, absent of novobiocin) versus the |LFC_CS_| after shifting to cold shock. The data informs on the 3915 genes (grey circles), for whom there is RNA-seq on both cold shock and novobiocin responses. The blue circles are the 367 cold shock repressed genes. As a null model, we randomized both |LFC| values of each gene (black dashed line). We also created cohorts of randomly selected, non cold shock repressed (non-CSR) genes, whose average mean LFC_CS_ was similar to that of cold shock repressed genes (violet dots show the example results of 1 of the 1000 randomly assembled cohorts). Best fit lines obtained by OLS. We performed an F-test on the linear regression model, to test for the null hypothesis that the first order polynomial does not significantly improve the fitting compared to a zero order polynomial. If p-values <0.05, the null hypothesis is rejected, and the best fit line is a first order polynomial. (**D**) Flow cytometry data on the effects of novobiocin over time on Ω. Data best fit by a sigmoid, ${\left(\frac{L}{{1 + {e}^{ - a( {x - {x}_0} )}}}\right)}$, where *L* is the maximum value, *x*_0_ is the sigmoid midpoint, and *a* is the curve steepness, which we set to 0.1 in order to maximize the *R*^2^ (we also attempted to fit polynomials up to several orders, but none fitted better). *Ω* values obtained for each time point, by fitting the single cell data with the function CV^2^ = Ω/*M* (34,74). Vertical error bars are the SEM.

From past studies (34), in general, transcription in optimal conditions can be well modeled as a one-step process (reaction (1.1) in Figure 6A). Using reaction (1.1) along with reactions for translation (reaction (2) in Figure 6A) and for RNA and protein decay due to degradation and dilution in cell division (reactions (3) and (4) in Figure 6A, respectively), one can model the approximate dynamics of RNA and protein numbers of a standard gene of *E. coli* (34). Assuming this model, we derived an analytical solution for *Ω* (Supplementary Section XIV.b and XVI):

(3) $$\begin{equation*}\Omega = 1 + \frac{{{k}_2}}{{{\lambda }_1 + {\lambda }_2}}\end{equation*}$$

Here, *k*_2_ is the translation rate and *λ*_1_ and *λ*_2_ are the RNA and protein decay rates, respectively.

Given Equation (3), since *λ*_1_ >> *λ*_2_ (34,76), *Ω* would necessarily be controlled by (*k*_2_/λ_1_). However, neither *k*_2_ nor λ_1_ are the likely regulators of *Ω* when changing from the control to the cold shock condition. Specifically, first, regarding *k*_2_, the response of the cold shock repressed genes occurs at the RNA level (detected 20 min after cold shock, Section *Identification of short-term cold shock repressed genes*), and the changes in the protein numbers of those genes during cold shock are correlated with the changes in the corresponding RNA numbers (Supplementary Figure S13B). Thus, the regulation of the cold shock repressed genes occurs at the transcription level. In addition, we failed to find any statistically significant differences in the ribosome binding site sequence of the RNAs coded by the cold shock repressed genes (which affects translation rates (77) and by randomly selected genes (Supplementary Figures S20 and S21 see also Supplementary Sections IX, X and XI), in what regards their Shine-Dalgarno (Supplementary Table S10) as well as their start codon sequences (Supplementary Table S11). Second, regarding *λ*_1_, in *E. coli* the RNA degradation rates do not correlate with the RNA sequence, abundance or metabolic function (57,78,79). Thus, we do not expect that the *λ*_1_ of cold shock repressed genes changes with cold shock in a manner that could explain their responsiveness to cold shock. Thus, a one-step model cannot explain the selection of the cold shock repressed genes in cold shock.

We therefore hypothesized that another mechanism, not present in the one-step model, ought to be responsible for the selective responsiveness of cold shock repressed genes. Such mechanism should explain the non-linear shift in the relationship between mean and noise (Figure 4B).

First, from past *in vitro* data from a synthetic promoter below 20°C (80–82), we hypothesized that cold shock could slow down the isomerization during open complex formation. This can be modeled by adding a rate-limiting step in transcription initiation (82), such as by replacing reaction 1.1 by reactions 1.2 (Figure 6A). To test this ‘two-steps’ model, we tuned it to match the one-step model in mean expression, and then compared their noise, CV^2^ (parameter values in Supplementary Table S3). From stochastic simulations (Methods Section *Stochastic model of cold shock response*), the additional rate limiting step reduces noise (Figure 6B). However, contrary to this, *Ω* is higher during cold shock. Thus, we rule out this two-steps model.

Next, since *Ω* is higher during cold shock, based on (4,83,84), we instead hypothesized that cold shock could lock some promoters’ activity, by disturbing DNA supercoiling. As in (83), this can be modeled by an ON–OFF process of promoter activity, by replacing reaction (1.1) by reactions (1.3) (Figure 6A). From stochastic simulations, setting the same mean expression (parameter values in Supplementary Table S3), this model dynamics is noisier (higher CV^2^, Figure 6B). Thus, an ON–OFF model could explain the increase in *Ω* at low temperatures.

Nevertheless, we cannot rule out the possibility that cold shock, by disturbing DNA supercoiling, can slow down the closed and/or the open complex formation, as well as increase the locking of promoter activity. This is possible provided that the combined outcome of both phenomena is the observed increase in *Ω*. In detail, maintaining equilibrium between the accumulation and the removal of supercoils may be energetically costly, given that the removal of positive supercoils is ATP dependent (85). Therefore, cells may not be able to maintain the same DNA supercoiling levels as in optimal conditions. This more complex scenario may be more likely than what model 1.3 assumes (i.e. the locking of promoter activity alone).

Specifically, first, DNA supercoiling affects, independently, both the closed complex (86), as well as the open complex formation (87,88). Mechanistically, DNA supercoiling can alter the 3D distances between sites along the DNA, which could increase the time length of closed complex formations (86). Also, DNA supercoiling can hamper the separation of the DNA strands, increasing the time length of the open complex formation (26,89). These effects on the closed and the open complex formations could be modeled by reducing the rates of the two-rate limiting steps in model 1.2.

Meanwhile, instead of delaying the times for completion of closed and open complex formation, the changes in DNA supercoiling may require the intervention of topoisomerases to resume transcription. Increases in the number of such events and/or in the dissociation times are best modelled by the ON–OFF reactions in model 1.3, particularly due to the limited number of topoisomerases in *E. coli* (83).

Overall, to account for both effects of cold shock, one could use a model that combines the two rate-limiting steps in transcription initiation with an ON–OFF process (model 1.4 in Supplementary Figure S22).

Finally, a comprehensive kinetic model of these effects during cold shock may further require that each step during initiation is independently subject to locking. For that, one could model two independent ON–OFF processes, one associated with the closed complex formation and the other with the open complex formation (model 1.5 in Supplementary Figure S22).

Unfortunately, our data on gene expression does not suffice to distinguish between models 1.3, 1.4 and 1.5. Thus, as in (83), from here onwards, we assume model 1.3 in Figure 6A to interpret the empirical data in Figure 4.

### Response strength to cold shock is correlated with reduced levels of negative supercoiling

We next explored the hypothesis that the short-term cold shock repression emerges from reduced negative supercoiling levels. For this, we performed RNA-seq after subjecting cells to 50 μg/ml novobiocin, which inhibits gyrase (90,91) (Methods Section *Bacterial strains, growth conditions, and gene expression measurements*) and, thus, would cause a similar effect as cold shock if the hypothesis holds true. We note that this partial inhibition of gyrase (83) may only diminish negative supercoiling, rather than accumulating positive supercoiling (Figure 1, panel IV).

From Figure 6C, the response strengths of cold shock repressed genes to novobiocin are positively correlated to their response strengths to cold shock (blue line in Figure 6C, *P*-value < 0.05), which supports the hypothesis. Further, cold shock repressed genes are more sensitive to novobiocin than other genes, which further supports that they are more supercoiling sensitive.

On the other hand, this could instead be because their original expression in the control condition was relatively high, when compared to the average gene. To test this, we compared the response strength to novobiocin of genes that are *not* cold shock repressed. Specifically, we selected cohorts of randomly selected non- cold shock repressed genes, with the same number of genes and the same average expression level in the optimal condition as the cold shock repressed cohort. We found that the best fitting line between their responses to cold shock and to novobiocin (Figure 6C) has a smaller slope than the line for cold shock repressed genes. Also, the two slopes can be statistically distinguished. We conclude that it is not their high expression level that explains why cold shock repressed genes are also supercoiling sensitive.

Finally, we confronted our results to past data on how antibiotics (novobiocin and norfloxacin) affect gene expression (24). From Supplementary Section XVII, we found a non-negligible correlation between our classification of genes as cold shock repressed, and their classification as supercoiling sensitive.

Given the above, we hypothesized that reduced negative supercoiling levels is a key underlying mechanism of the short-term transcriptional program of cold shock responsiveness. To find if cold shock repressed genes are also supercoiling sensitive, we considered the genes whose responses to cold shock were stronger. To select them, we set a threshold between weak and strong at |LFC_CS_| = 0.8 (Supplementary Figure S23) since, below it, several p-values are close to the significance level (Supplementary Section I).

Next, to investigate if genes with strong cold shock repressed also have high supercoiling sensitivity, we also needed to classify genes as having ‘high’ |LFC_NOVO_|. For this, we considered that the inclination of the best fitting lines in Figure 6C likely differ with perturbation strengths (e.g. adding more than 50 μg/ml novobiocin would cause stronger LFCs (84)). Since, on average, the response strength of cold shock repressed genes to 15°C was twice as strong as to 50 μg/ml novobiocin (for which responses were classified as strong if |LFC_CS_| > 0.8), we classified responses of |LFC_NOVO_| > 0.4 as ‘strong’ (and *P*-value < 0.05). Given this, 1215 out of 3948 genes of *E. coli* (∼31%) were classified as having a strong response to novobiocin.

Given these classifications, of the 381 genes classified as cold shock repressed, 201 are strongly responsive to cold shock. Of these, we considered only 190, since the remaining 11 failed to obey the filtering criteria *iii*, in step I.c. in Supplementary Section I. Of the 190, there are 92 genes (i.e. ∼48%) strongly responsive to novobiocin. That is, approximately half of the cold shock repressed genes are strongly supercoiling sensitive, which is higher than expected by random chance. Further, from Supplementary Figure S24, the more supercoiling sensitive a gene is, the more likely that it is also cold shock repressed. Thus, we conclude that high supercoiling sensitivity is at the core of the short-term, cold shock responsive transcriptional program of *E. coli*.

However, we also find that supercoiling sensitivity is not the only means by which genes can be quickly repressed during cold shock (52% of the strong cold shock repressed genes are not strongly supercoiling sensitive). Further, while of 3948 genes of *E. coli*, 1215 exhibited |LFC_NOVO_| > 0.4 (*P*-value < 0.05), only 92 of these 1215 are strong cold shock repressed genes. Similarly, not all the 100 genes with highest |LFC_NOVO_| are cold shock repressed. Combined, these three results suggest that being strongly supercoiling sensitive is not sufficient for being a short-term cold shock repressed gene. We thereby hypothesized that cold shock repressed genes that are strongly supercoiling sensitive have an additional feature that, combined with strong supercoiling sensitivity, makes them cold shock repressed.

Next, we investigated whether the conclusions above differ significantly when setting different thresholds for the definitions of strongly supercoiling sensitive and strongly cold shock repressed genes. We found that, for the wide range of values tested, the probability of a strongly cold shock repressed gene to be also strongly supercoiling sensitive is always above chance (Supplementary Figure S24). This suggests that our conclusions, qualitatively, are not affected by the threshold setting.

Finally, we studied whether some features, other than strong supercoiling sensitivity, could explain the genes’ responsiveness to novobiocin. Specifically, we tested the potential role of transcription factor interactions (including global regulators), of closely spaced promoters, and of the sensitivity to (p)ppGpp. However, we failed to find any correlations with the responsiveness to novobiocin (Supplementary Section XVIII, Supplementary Figure S25). The numbers of genes responsive to these factors and combinations of factors are shown in Supplementary Figure S11B.

Given the above, from *in vivo* single-cell, time lapse protein data (Methods Section *Bacterial strains, growth conditions, and gene expression measurements*), we studied the dynamics of the six genes used to produce Figure 4B and investigated if their cold shock responsiveness is due to their supercoiling sensitivity. In detail, if during cold shock, a rate-limiting step emerges in their dynamics (reaction (1.3) in the ON-OFF model in Figure 6A), we expect that the noise for a given mean expression level should be higher than during optimal conditions. For this, we further measured four additional cold shock repressed genes because they, in addition to pepN and ndk of the six genes, are the only ones out of the 381 cold shock repressed genes that: (i) do not have any known input TFs and, thus, even in optimal conditions, should be less influenced by the TF network of *E. coli*; (ii) their expression levels in control conditions are above background noise, and; (iii) they are not integrated in a position of an operon structure other than the first one downstream the promoter.

Results in Supplementary Figure S26 show that, in accordance with the predictions, there is a decrease in mean protein levels during cold shock and gyrase inhibition. Only two genes, pepN and feoA, exhibit increased levels, contrary to the model, after 60 and 100 min following the addition of novobiocin, respectively. This is, potentially, due to mid- and long-term phenomena (also respectively) occurring as part of the cellular response program to cold. For example, feoA has 4 input TFs, while pepN is closely spaced to another gene, ssuB, in a convergent configuration. Also, ssuB has no transcription termination site. As such, it can perturb pepN’s expression, e.g. by first repressing and then stopping doing so, when under the effects of novobiocin.

Meanwhile, *Ω*, following gyrase inhibition, (Figure 6D) fits well by a sigmoid, as it did when subjecting cells to cold shock (Figure 4B). The main difference between Figures 4B and 6D is that it takes less 20 min for the shift to occur following novobiocin addition. This might be due to the slowing down of metabolic events during cold.

Finally, we note that the similarity in the mean changes in *Ω* is not used as criteria to support that the underlying mechanism is the same, because we tuned the novobiocin levels to make the average strength of the perturbations similar.

### The engagement between gyrases and nucleoid increases during cold shock

**Figure 7.**
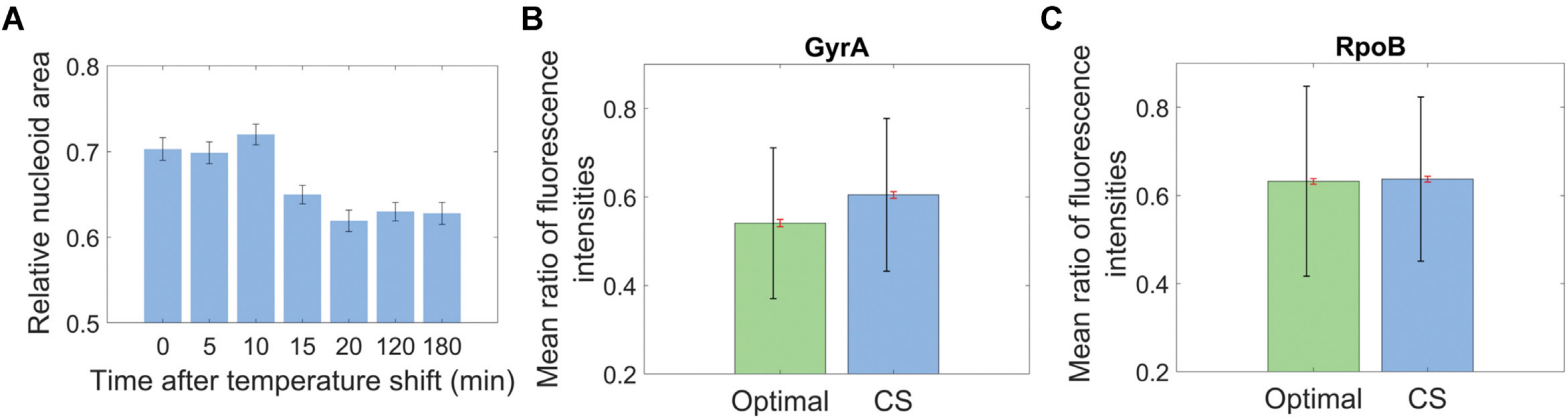
Biophysical parameters during cold shock. (**A**) Nucleoid areas relative to the cell areas following cold shock (CS) over time. The error bars are the SEM. More than 500 cells analyzed per time moment (different cells analyzed in each time moment). (**B**) Mean ratio of fluorescence intensities from GyrA-YFP inside the nucleoid and the total GyrA-YFP inside the cell. (**C**) Mean ratio of fluorescence intensities between RpoB-YFP inside the nucleoid and the total RpoB-YFP inside the cell. In (B) and (C) more than 400 cells were analyzed per condition. The red error bars are the SEM and the black error bars are the standard deviation (STD).

If supercoiling sensitivity is one of the triggers of genes’ responsiveness to cold shock, we expect global DNA supercoiling levels to differ in cold shock. This difference could be indirectly detected, since it should influence the nucleoid volume, given that supercoiling affects DNA compaction (17,19,92). In agreement, we observed that the nucleoid areas (used as a proxy for nucleoid volume) decreased during cold shock (Figure 7A and Methods Section *Nucleoid visualization by DAPI*), while the cell area remained relatively undisturbed (Figures 2C and Supplementary Figure S5B).

Meanwhile, we also observed correlated single-gene responses to cold shock and to novobiocin (Figure 6C). Thus, similarly to novobiocin, cold shock may increase the mean time to resolve (at least, but not restricted to) positive supercoils. This would result in an higher level of engagement between gyrase and the DNA, since most gyrases are involved in maintaining the chromosome supercoiling level in a steady state (93). To test for increased engagement, we performed microscopy following DAPI staining of cells expressing GyrA-YFP (example cell images in Supplementary Figure S27). Next, we quantified the fluorescence from GyrA-YFP in the nucleoid region, relative to the total GyrA-YFP fluorescence in the cell (Methods Section *Microscopy image analysis*). We found a small, but statistically significant increase during cold shock (∼6%), suggesting increased gyrase engagement with the nucleoid (Figure 7B). In detail, while the error bars of the standard deviation overlap, we conclude that the means differ because their standard errors do not overlap. In agreement, a two-sample *t*-test rejected the null hypothesis that the datasets are from independent random samples with equal means, with *P*-value < 10^–6^.

Overall, these results are consistent with an enhanced difficulty, during cold shock, for topoisomerases (particularly gyrases), to maintain the global negative supercoiling at the same level as in optimal conditions. This is more accurately represented by models that incorporate an ON–OFF process, triggered by changes in DNA supercoiling, and requiring topoisomerase intervention for transcription to be resumed (Figure 6A).

For comparison, we observed RpoB-YFP (example cell images in Supplementary Figure S28). In this case, we did not find evidence for changed engagement with the DNA during cold shock, in that the relative fluorescence in the nucleoid region did not change (Figure 7C). Specifically, a two-sample t-test did not reject the null hypothesis that the datasets are from independent random samples with equal means (p-value of 0.58). This implies that the change did not occur in all DNA binding proteins. Noteworthy, in agreement with Figures 7B and C, Supplementary Figures S27 and S28 (data from optimal conditions), the RNAP cloud appears to be closer to the nucleoid(s) centre(s) than the gyrase cloud.

Other studies have measured changes in the engagement of DNA binding proteins with DNA damage (94–97) and with drugs preventing gyrase and topoisomerase IV from unbinding the DNA (93,98). Super-resolution time-lapse microscopy showed distinguished specific and non-specific bindings (with the latter being transient interactions that do not lead to catalysis). Since we are unable to distinguish between these two forms of binding, we cannot conclude if the 6% increase in gyrase engagement can cause changes in DNA supercoiling (which would require changes in specific bindings).

### Cellular energy levels decrease during cold shock

Gyrase removal of positive supercoils requires ATP binding (85,99,100). Novobiocin, which we used above to inhibit gyrase activity, acts by hampering that binding (101). Given this, and since gyrase numbers did not change during cold shock (Figure 2D), a decrease in ATP levels could explain the increased difficulty in resolving positive supercoils during cold shock.

We measured ATP in the control and in cold shock conditions (Methods Section *Cellular ATP levels*, Supplementary Section XX). We observed that ATP levels decrease during cold shock (Supplementary Figure S2). This decrease can explain why the mean escape time from OFF states increased in cold shock, albeit gyrase numbers did not change, which supports the ON-OFF model.

To test this, we caused a similar decrease in ATP levels, by a method other than cold shock. Namely, we subjected cells to 2,4-dinitrophenol (DNP), which uncouples the oxidative phosphorylation, causing ATP depletion (102). We observed increasing ATP depletion with increasing DNP concentration (Supplementary Figure S29A).

Meanwhile, we also observed that DNP affects cell growth rates when above 100 μM (Supplementary Figure S29B). To avoid changes in cell growth rates, since this can have unknown effects, we next subjected cells to 100 μM of DNP. This concentration caused an ATP depletion of 40%, which is similar to the ATP depletion of 46% during cold shock (120 min after shifted to cold shock, Supplementary Figure S2).

Interestingly, this caused changes in the *Ω* of cold shock repressed genes (Supplementary Figure S29C), similar to cold shock (15°C for 120 min, Supplementary Figure S16A) and to gyrase inhibition (Figure 6D). This suggests that ATP depletion during cold shock, affects gyrase activity, which affects gene expression.

### Relative Ω as a function of OFF-ON rates in cold shock repressed genes

Having this, we expect cold shock to alter how *Ω* is regulated due to the emergence of an ON–OFF step controlling transcription. We estimated the expected ratio between values of *Ω* at cold and control conditions assuming ON–OFF and one-step models (Figure 6A), respectively (Supplementary Section XIV.c-e, Supplementary Table S12). From there:

(4) $$\begin{equation*}\frac{{{\Omega }_{CS}}}{{{\Omega }_{CTRL}}} \propto \left( {1 + \frac{{{k}_1.{k}_ - }}{{{{({k}_ + + {k}_ - )}}^2}}} \right)\end{equation*}$$

Equation (4) informs on how the ratio ${\Omega }_{CS}/{\Omega }_{CTRL}$ is expected to be affected by the rate constants controlling the ON–OFF steps, *k_+_* and *k_-_* (reactions 1.3 in Figure 6A), and the average transcription activity from active promoters, *k*_1_ (Supplementary Section XII).

We do not expect *k*_1_ to be a major regulator of this ratio, since this rate constant is present in the one-step model, which was unable to mimic the measurements. Meanwhile, of the two remaining events controlling promoter activity (reactions (1.3), Figure 6A) only promoter escape from the OFF state (regulated by *k_+_*) is energy consuming (100). Thus, this event is expected to be most decelerated one during cold shock. We therefore hypothesized that *k_+_* is the most temperature sensitive parameter in Equation (4).

We therefore investigated the relationship between *k_+_* and temperature. We explored four temperature sensitive models of *k_+_* which were fitted to the empirical data from Figure 4B. Models and best fitting parameter values are shown in Supplementary Table S13, while results of the fitting are shown in Supplementary Figure S30. From the *R*^2^ values, the best fitting model assumes that *k_+_* changes over time following an exponential function.

## DISCUSSION

We identified a large number of short-term cold shock repressed genes and studied what causes their quick repression in cold shock. A few of them are likely responsive due to being in an operon with upstream cold shock repressed genes, (p)ppGpp sensitivity, etc., but the majority appears to be independently responsive to cold shock.

Interestingly, following cold shock, cold shock repressed genes rapidly decrease expression level, while their noise relative to the mean expression increases. This noise increase is consistent with the emergence of transient locking events during transcription. Since a similar phenomenon was observed following gyrase inhibition (83,84) and because we observed here that nearly half of the cold shock repressed genes are also highly supercoiling sensitive, we hypothesized that their responsiveness emerges from their supercoiling sensitivity. Meanwhile, we also observed that gyrase converges to the nucleoid and that cell energy decreases during cold shock, suggesting that the number of promoters locked due to supercoiling sensitivity increases during cold shock. We therefore proposed a model of the responsiveness of cold shock repressed genes based on their temperature-dependent supercoiling sensitivity.

To our knowledge, temperature-dependent supercoiling is the first identified physical mechanism of how *E. coli* genes can be cold shock repressed. Our data suggests that it contributes to the cold shock responsiveness of, potentially, nearly half of the cold shock repressed genes. Mechanically, it may act by locking transcription initiation, which can be modelled as a ON–OFF process. Physically, this might emerge from lower negative DNA supercoiling during cold shock, which may alter the 3-dimensional distances between DNA sites and/or the unwinding of the DNA strands (86,89). These alterations may hamper the closed and/or open complex formation, until the intervention of topoisomerases. Contrary to this, the other half of cold shock repressed genes were not strongly responsive to novobiocin, suggesting that their cold shock repression cannot be solved by gyrases. This may be because their repression was mechanistically different, potentially because other transcription steps were affected (RNAP promoter escape, elongation, translation initiation, etc.). The answer could be explored by, e.g. studying how these genes respond to antibiotics (other than novobiocin) targeting different events in transcription. Their means of repression could then be incorporated in model 1.5 in Supplementary Figure S22, which includes both two rate-limiting steps as well as ON-OFF mechanisms affecting each step. Nevertheless, more complex models including other sub-processes (e.g. how ATP affects gyrase activity), may be required.

The existence of a mechanism relying on temperature-dependent supercoiling, first hypothesized in (16), opens an avenue for the engineering of synthetic, temperature sensitive and temperature resistant gene regulatory circuits, whose functioning could be tuned by the adaptive regulation of gyrase activity. Further, we expect that it will contribute to learning how the short- and long-term transcriptional programs of *E. coli* responsive to cold shock have evolved.

Meanwhile, we also found that neither all short-term cold shock repressed genes are strongly supercoiling sensitive, nor all strongly supercoiling sensitive genes are short-term cold shock repressed. Thus, for genes to be short-term cold shock repressed due to their strong supercoiling sensitivity, they likely need an additional intrinsic feature(s). Further, in the introduction we reported that past studies suggested that DNA supercoiling can tune gene expression responses to several stresses other than cold shock (29–33). Perhaps, the combination of strong supercoiling sensitivity with another feature may be a widely used means by which cohorts of responsive genes to specific stresses operate, with the second feature differing between the stresses/cohorts.

Given this, much study is needed to identify the set of features that can make a gene cold shock repressed. Potentially, genes could be cold shock repressed by cold shock-based locking of their rate-limiting steps (103) during transcription initiation, as reported for a synthetic promoter (82). This could explain how, in 4 out of the 30 genes measured at the protein level, noise did not increase, although the mean levels decreased. Finally, it may be that, in some genes, their RNA or proteins have increased decay rates during cold shock, rather than altered production rates.

Another aspect requiring much study is how supercoiling levels percolate genes in the same operon in a manner that, while some genes downstream a cold shock repressed gene are also cold shock repressed, many are not.

From the present data alone, we cannot discern all causes for the changes in gene expression during cold shock. First, only half of the cold shock repressed genes are strongly responsive to novobiocin, thus, other causes must exist. Also, we expect cold shock to affect the activities of other, if not all, topoisomerases as well. Further, we expect their activities to not be altered similarly, since, e.g. the removal of negative supercoils is not ATP-dependent, while of positive supercoils is (85,99,100). Potentially, RNA-seq data following perturbations of the other topoisomerases (e.g. using target-specific antibiotics as well) may reveal similar changes in RNA levels to what was observed during cold shock in the same or in other cold shock repressed genes (whose response mechanisms are yet to identify). Moreover, other regulators influencing DNA relaxation, such as RNAP (27), DNA polymerase (104) and DNA binding proteins such as HU, H-NS, etc. (105), are also perturbed by cold shock (even indirectly, due to increased cytoplasm viscosity (12,13)). Overall, all these perturbations may alter the equilibrium between the removal and the accumulation of positive supercoils, causing changes in DNA relaxation.

Bacterial transcriptional programs of cold shock responsiveness are critical survival skills that indirectly affect a wide range of vital Human activities (3). Meanwhile, since in *E. coli* nearly half of the short-term cold shock repressed genes may rely on supercoiling sensitivity, interfering with them could allow for wide changes in bacterial cold shock response programs. As such, this may be a viable strategy with potentially great rewards.

For example, bacteria have evolved to prioritize survival and growth rates, rather than to produce components at low energetic costs. Reverting this in the transcriptional program of cold shock response of genetically modified bacteria will be of value in biotechnological applications (e.g. by making bioreactors more energy efficient). One means to silence this cold shock program could be achieved by introducing a synthetic circuit with stronger sensitivity to low temperatures, e.g. by having the component synthetic genes with high supercoiling sensitivity. Meanwhile, this could be achieved by tuning (directly or indirectly) its genes’ supercoiling sensitivity, potentially without disturbing the natural system. Means to implement this tuning may include altering the DNA location of cold shock repressed genes (106,107) and of strong gyrase binding sites (108,109). Another strategy could be to alter promoter spacer and discriminator sequences (110,111) and closely spaced promoter configurations (112) of the synthetic genes since, albeit not influencing most natural cold shock repressed genes, these mechanisms could, in theory, be used to tune (at least indirectly) the supercoiling sensitivity of some genes.

These strategies could be used to either enhance or decrease the responsiveness of genes to cold shock. The former would assist in engineering low-temperature bioreactors (e.g. used for fermentation in the dairy industry). The latter could enhance biofertilization and plant resistance to bacteria, among others (3).

## DATA AVAILABILITY

RNA-seq *.fastq data and processed data under cold shock and novobiocin are deposited in NCBI GEO with accession code GSE194037. Meanwhile, since the raw RNA-seq data (control condition) was already used in (113,114), please access it using its NCBI GEO accession code GSE183139. Another data package was deposited in Dryad with flow cytometry, microscopy, and plate reader data (DOI: 10.5061/dryad.pvmcvdnnm).

## Supplementary Material

gkac643_Supplemental_Files

## References

[1] Phadtare S. , Alsina J., Inouye M. Cold-shock response and cold-shock proteins. Curr. Opin. Microbiol. 1999; 2:175–180. 10322168 10.1016/S1369-5274(99)80031-9

[2] Jones P.G. , VanBogelen R.A., Neidhardt F.C. Induction of proteins in response to low temperature in *Escherichia coli*. J. Bacteriol. 1987; 169:2092–2095. 3553157 10.1128/jb.169.5.2092-2095.1987 PMC212099

[3] Phadtare S. Recent developments in bacterial cold-shock response. Curr. Issues Mol. Biol. 2004; 6:125–136. 15119823

[4] Oliveira S.M.D. , Häkkinen A., Lloyd-Price J., Tran H., Kandavalli V., Ribeiro A.S. Temperature-dependent model of Multi-step transcription initiation in Escherichia coli based on live single-cell measurements. PLoS Comput. Biol. 2016; 12:e1005174. 27792724 10.1371/journal.pcbi.1005174 PMC5085040

[5] Charlebois D.A. , Hauser K., Marshall S., Balázsi G. Multiscale effects of heating and cooling on genes and gene networks. Proc. Natl. Acad. Sci. U.S.A. 2018; 115:E10797–E10806. 30341217 10.1073/pnas.1810858115 PMC6233105

[6] Giuliodori A.M. , Brandi A., Gualerzi C.O., Pon C.L. Preferential translation of cold-shock mRNAs during cold adaptation. RNA. 2004; 10:265–276. 14730025 10.1261/rna.5164904 PMC1370538

[7] Farewell A. , Neidhardt F.C. Effect of temperature on in vivo protein synthetic capacity in Escherichia coli. J. Bacteriol. 1998; 180:4704–4710. 9721314 10.1128/jb.180.17.4704-4710.1998 PMC107486

[8] Keto-Timonen R. , Hietala N., Palonen E., Hakakorpi A., Lindström M., Korkeala H. Cold shock proteins: a minireview with special emphasis on Csp-family of enteropathogenic yersinia. Front. Microbiol. 2016; 7:1151. 27499753 10.3389/fmicb.2016.01151 PMC4956666

[9] Madrid C. , Nieto J.M., Paytubi S., Falconi M., Gualerzi C.O., Juárez A. Temperature- and H-NS-Dependent regulation of a plasmid-encoded virulence operon expressing Escherichia coli hemolysin. J. Bacteriol. 2002; 184:5058–5066. 12193622 10.1128/JB.184.18.5058-5066.2002 PMC135304

[10] Mansilla M.C. , Cybulski L.E., Albanesi D., de Mendoza D Control of membrane lipid fluidity by molecular thermosensors. J. Bacteriol. 2004; 186:6681–6688. 15466018 10.1128/JB.186.20.6681-6688.2004 PMC522199

[11] Yamanaka K. Cold shock response in Escherichia coli. J. Mol. Microbiol. Biotechnol. 1999; 1:193–202. 10943550

[12] Parry B.R. , Surovtsev I.V., Cabeen M.T., O’Hern C.S., Dufresne E.R., Jacobs-Wagner C. The bacterial cytoplasm has Glass-like properties and is fluidized by metabolic activity. Cell. 2014; 156:183–194. 24361104 10.1016/j.cell.2013.11.028 PMC3956598

[13] Oliveira S.M.D. , Neeli-Venkata R., Goncalves N.S.M., Santinha J.A., Martins L., Tran H., Mäkelä J., Gupta A., Barandas M., Häkkinen A. et al. Increased cytoplasm viscosity hampers aggregate polar segregation in Escherichia coli. Mol. Microbiol. 2016; 99:686–699. 26507787 10.1111/mmi.13257

[14] Phadtare S. , Inouye M. Genome-wide transcriptional analysis of the cold shock response in wild-type and cold-sensitive, quadruple-csp-deletion strains of Escherichia coli. J. Bacteriol. 2004; 186:7007–7014. 15466053 10.1128/JB.186.20.7007-7014.2004 PMC522181

[15] Arsène F. , Tomoyasu T., Bukau B. The heat shock response of Escherichia coli. Int. J. Food Microbiol. 2000; 55:3–9. 10791710 10.1016/s0168-1605(00)00206-3

[16] Oliveira S.M.D. , Goncalves N.S.M., Kandavalli V.K., Martins L., Neeli-Venkata R., Reyelt J., Fonseca J.M., Lloyd-Price J., Kranz H., Ribeiro A.S. Chromosome and plasmid-borne PLacO3O1 promoters differ in sensitivity to critically low temperatures. Sci. Rep. 2019; 9:4486. 30872616 10.1038/s41598-019-39618-z PMC6418193

[17] Goldstein E. , Drlica K. Regulation of bacterial DNA supercoiling: plasmid linking numbers vary with growth temperature. Proc. Natl. Acad. Sci. U.S.A. 1984; 81:4046–4050. 6377307 10.1073/pnas.81.13.4046 PMC345365

[18] López-García P. , Forterre P. DNA topology and the thermal stress response, a tale from mesophiles and hyperthermophiles. Bioessays. 2000; 22:738–746. 10918304 10.1002/1521-1878(200008)22:8<738::AID-BIES7>3.0.CO;2-5

[19] Stuger R. , Woldringh C.L., van der Weijden C.C., Vischer N.O.E., Bakker B.M., van Spanning R.J.M., Snoep J.L., Westerhoff H.V. DNA supercoiling by gyrase is linked to nucleoid compaction. Mol. Biol. Rep. 2002; 29:79–82. 12241080 10.1023/a:1020318705894

[20] Holmes V.F. , Cozzarelli N.R. Closing the ring: links between SMC proteins and chromosome partitioning, condensation, and supercoiling. Proc. Natl. Acad. Sci. U.S.A. 2000; 97:1322–1324. 10677457 10.1073/pnas.040576797 PMC34294

[21] Travers A. , Muskhelishvili G. DNA supercoiling — a global transcriptional regulator for enterobacterial growth?. Nat. Rev. Microbiol. 2005; 3:157–169. 15685225 10.1038/nrmicro1088

[22] Dorman C.J. DNA supercoiling and bacterial gene expression. Sci. Prog. 2006; 89:151–166. 17338437 10.3184/003685006783238317 PMC10368349

[23] Dorman C.J. , Dorman M.J. DNA supercoiling is a fundamental regulatory principle in the control of bacterial gene expression. Biophys. Rev. 2016; 8:89–100. 28510216 10.1007/s12551-016-0238-2 PMC5418507

[24] Peter B.J. , Arsuaga J., Breier A.M., Khodursky A.B., Brown P.O., Cozzarelli N.R. Genomic transcriptional response to loss of chromosomal supercoiling in Escherichia coli. Genome Biol. 2004; 5:R87. 15535863 10.1186/gb-2004-5-11-r87 PMC545778

[25] Amit R. , Oppenheim A.B., Stavans J. Increased bending rigidity of single DNA molecules by H-NS, a temperature and osmolarity sensor. Biophys. J. 2003; 84:2467–2473. 12668454 10.1016/S0006-3495(03)75051-6 PMC1302812

[26] Pruss G.J. , Drlica K. DNA supercoiling and prokaryotic transcription. Cell. 1989; 56:521–523. 2645054 10.1016/0092-8674(89)90574-6

[27] Liu L.F. , Wang J.C. Supercoiling of the DNA template during transcription. Proc. Natl. Acad. Sci. U.S.A. 1987; 84:7024–7027. 2823250 10.1073/pnas.84.20.7024 PMC299221

[28] Ma J. , Bai L., Wang M.D. Transcription under torsion. Science. 2013; 340:1580–1583. 23812716 10.1126/science.1235441 PMC5657242

[29] Cheung K.J. , Badarinarayana V., Selinger D.W., Janse D., Church G.M. A microarray-based antibiotic screen identifies a regulatory role for supercoiling in the osmotic stress response of *Escherichia coli*. Genome Res. 2003; 13:206–215. 12566398 10.1101/gr.401003 PMC420364

[30] Weinstein-Fischer D. , Elgrably-Weiss M., Altuvia S. Escherichia coli response to hydrogen peroxide: a role for DNA supercoiling, topoisomerase I and fis. Mol. Microbiol. 2000; 35:1413–1420. 10760142 10.1046/j.1365-2958.2000.01805.x

[31] Drlica K. Control of bacterial DNA supercoiling. Mol. Microbiol. 1992; 6:425–433. 1313943 10.1111/j.1365-2958.1992.tb01486.x

[32] Dorman C.J. Flexible response: DNA supercoiling, transcription and bacterial adaptation to environmental stress. Trends Microbiol. 1996; 4:214–216. 8795154 10.1016/0966-842X(96)30015-2

[33] Dorman C.J. 1DNA topology and the global control of bacterial gene expression: implications for the regulation of virulence gene expression. Microbiology. 1995; 141:1271–1280. 7670631 10.1099/13500872-141-6-1271

[34] Taniguchi Y. , Choi P.J., Li G.-W., Chen H., Babu M., Hearn J., Emili A., Xie X.S. Quantifying *E. coli* proteome and transcriptome with single-molecule sensitivity in single cells. Science. 2010; 329:533–538. 20671182 10.1126/science.1188308 PMC2922915

[35] Ma D. , Cook D.N., Alberti M., Pon N.G., Nikaido H., Hearst J.E. Genes acrA and acrB encode a stress-induced efflux system of Escherichia coli. Mol. Microbiol. 1995; 16:45–55. 7651136 10.1111/j.1365-2958.1995.tb02390.x

[36] Ma D. , Cook D.N., Hearst J.E., Nikaido H. Efflux pumps and drug resistance in gram-negative bacteria. Trends Microbiol. 1994; 2:489–493. 7889326 10.1016/0966-842x(94)90654-8

[37] Patange O. , Schwall C., Jones M., Villava C., Griffith D.A., Phillips A., Locke J.C.W. *Escherichia coli* can survive stress by noisy growth modulation. Nat. Commun. 2018; 9:5333. 30559445 10.1038/s41467-018-07702-z PMC6297224

[38] Yaginuma H. , Kawai S., Tabata K.V., Tomiyama K., Kakizuka A., Komatsuzaki T., Noji H., Imamura H. Diversity in ATP concentrations in a single bacterial cell population revealed by quantitative single-cell imaging. Sci. Rep. 2014; 4:6522. 25283467 10.1038/srep06522 PMC4185378

[39] Bahrudeen M.N.M. , Chauhan V., Palma C.S.D., Oliveira S.M.D., Kandavalli V.K., Ribeiro A.S. Estimating RNA numbers in single cells by RNA fluorescent tagging and flow cytometry. J. Microbiol. Methods. 2019; 166:105745. 31654657 10.1016/j.mimet.2019.105745

[40] Cunningham A. Fluorescence pulse shape as a morphological indicator in the analysis of colonial microalgae by flow cytometry. J. Microbiol. Methods. 1990; 11:27–36.

[41] Traganos F. Flow cytometry: principles and applications. I. Cancer Invest. 1984; 2:149–163. 6203624 10.3109/07357908409020296

[42] Chazotte B. Labeling nuclear DNA using DAPI. Cold Spring Harb. Protoc. 2011; 2011:db.prot5556. 10.1101/pdb.prot5556 21205856

[43] Ribeiro A.S. , Lloyd-Price J. SGN sim, a stochastic genetic networks simulator. Bioinformatics. 2007; 23:777–779. 17267430 10.1093/bioinformatics/btm004

[44] Gillespie D.T. A general method for numerically simulating the stochastic time evolution of coupled chemical reactions. J. Comput. Phys. 1976; 22:403–434.

[45] Gillespie D.T. Exact stochastic simulation of coupled chemical reactions. J. Phys. Chem. 1977; 81:2340–2361.

[46] Häkkinen A. , Ribeiro A.S. Characterizing rate limiting steps in transcription from RNA production times in live cells. Bioinformatics. 2016; 32:1346–1352. 26722120 10.1093/bioinformatics/btv744

[47] Santos-Zavaleta A. , Salgado H., Gama-Castro S., Sánchez-Pérez M., Gómez-Romero L., Ledezma-Tejeida D., García-Sotelo J.S., Alquicira-Hernández K., Muñiz-Rascado L.J., Peña-Loredo P. et al. RegulonDB v 10.5: tackling challenges to unify classic and high throughput knowledge of gene regulation in E. coli K-12. Nucleic Acids Res. 2018; 47:D212–D220. 10.1093/nar/gky1077 PMC6324031 30395280

[48] Häkkinen A. , Muthukrishnan A.-B., Mora A., Fonseca J.M., Ribeiro A.S. CellAging: a tool to study segregation and partitioning in division in cell lineages of Escherichia coli. Bioinformatics. 2013; 29:1708–1709. 23613488 10.1093/bioinformatics/btt194

[49] Martins L. , Neeli-Venkata R., Oliveira S.M.D., Häkkinen A., Ribeiro A.S., Fonseca J.M. SCIP: a single-cell image processor toolbox. Bioinformatics. 2018; 34:4318–4320. 29931314 10.1093/bioinformatics/bty505

[50] Lange R. , Hengge-Aronis R. Identification of a central regulator of stationary-phase gene expression in Escherichia coli. Mol. Microbiol. 1991; 5:49–59. 1849609 10.1111/j.1365-2958.1991.tb01825.x

[51] Jishage M. , Iwata A., Ueda S., Ishihama A. Regulation of RNA polymerase sigma subunit synthesis in Escherichia coli: intracellular levels of four species of sigma subunit under various growth conditions. J. Bacteriol. 1996; 178:5447–5451. 8808934 10.1128/jb.178.18.5447-5451.1996 PMC178365

[52] Menzel R. , Gellert M. Regulation of the genes for E. coli DNA gyrase: homeostatic control of DNA supercoiling. Cell. 1983; 34:105–113. 6309403 10.1016/0092-8674(83)90140-x

[53] Dages S. , Dages K., Zhi X., Leng F. Inhibition of the gyrA promoter by transcription-coupled DNA supercoiling in Escherichia coli. Sci. Rep. 2018; 8:14759. 30282997 10.1038/s41598-018-33089-4 PMC6170449

[54] Gellert M. , Mizuuchi K., O’Dea M.H., Nash H.A. DNA gyrase: an enzyme that introduces superhelical turns into DNA. Proc. Natl. Acad. Sci. U.S.A. 1976; 73:3872–3876. 186775 10.1073/pnas.73.11.3872 PMC431247

[55] Wang J.C. Interaction between DNA and an *Escherichia coli* protein ω. J. Mol. Biol. 1971; 55:523–533. 4927945 10.1016/0022-2836(71)90334-2

[56] Kirkegaard K. , Wang J.C. Bacterial DNA topoisomerase I can relax positively supercoiled DNA containing a single-stranded loop. J. Mol. Biol. 1985; 185:625–637. 2997454 10.1016/0022-2836(85)90075-0

[57] Bernstein J.A. , Khodursky A.B., Lin P.-H., Lin-Chao S., Cohen S.N. Global analysis of mRNA decay and abundance in Escherichia coli at single-gene resolution using two-color fluorescent DNA microarrays. Proc. Natl. Acad. Sci. U.S.A. 2002; 99:9697–9702. 12119387 10.1073/pnas.112318199 PMC124983

[58] Ashburner M. , Ball C.A., Blake J.A., Botstein D., Butler H., Cherry J.M., Davis A.P., Dolinski K., Dwight S.S., Eppig J.T. et al. Gene ontology: tool for the unification of biology. Nat. Genet. 2000; 25:25–29. 10802651 10.1038/75556 PMC3037419

[59] Gene Ontology Consortium The gene ontology resource: enriching a GOld mine. Nucleic Acids Res. 2021; 49:D325–D334. 33290552 10.1093/nar/gkaa1113 PMC7779012

[60] Gadgil M. , Kapur V., Hu W.-S. Transcriptional response of Escherichia coli to temperature shift. Biotechnol. Prog. 2005; 21:689–699. 15932244 10.1021/bp049630l

[61] Andersen KB , von Meyenburg K Are growth rates of Escherichia coli in batch cultures limited by respiration?. J. Bacteriol. 1980; 144:114–123. 6998942 10.1128/jb.144.1.114-123.1980 PMC294601

[62] Balleza E. , Kim J.M., Cluzel P. Systematic characterization of maturation time of fluorescent proteins in living cells. Nat. Methods. 2018; 15:47–51. 29320486 10.1038/nmeth.4509 PMC5765880

[63] Hebisch E. , Knebel J., Landsberg J., Frey E., Leisner M. High variation of fluorescence protein maturation times in closely related Escherichia coli strains. PLoS One. 2013; 8:e75991. 24155882 10.1371/journal.pone.0075991 PMC3796512

[64] Maurizi M.R. Proteases and protein degradation in *Escherichia coli*. Experientia. 1992; 48:178–201. 1740190 10.1007/BF01923511

[65] Liu M. , Tolstorukov M., Zhurkin V., Garges S., Adhya S. A mutant spacer sequence between -35 and -10 elements makes the P_lac_ promoter hyperactive and cAMP receptor protein-independent. Proc. Natl. Acad. Sci. U.S.A. 2004; 101:6911–6916. 15118087 10.1073/pnas.0401929101 PMC406441

[66] Jacob F. , Monod J. Genetic regulatory mechanisms in the synthesis of proteins. J. Mol. Biol. 1961; 3:318–356. 13718526 10.1016/s0022-2836(61)80072-7

[67] Sabatti C. , Rohlin L., Oh M.-K., Liao J.C. Co-expression pattern from DNA microarray experiments as a tool for operon prediction. Nucleic Acids Res. 2002; 30:2886–2893. 12087173 10.1093/nar/gkf388 PMC117043

[68] Jones P.G. , Cashel M., Glaser G., Neidhardt F.C. Function of a relaxed-like state following temperature downshifts in Escherichia coli. J. Bacteriol. 1992; 174:3903–3914. 1597413 10.1128/jb.174.12.3903-3914.1992 PMC206098

[69] Sanchez-Vazquez P. , Dewey C.N., Kitten N., Ross W., Gourse R.L. Genome-wide effects on Escherichia coli transcription from ppGpp binding to its two sites on RNA polymerase. Proc. Natl. Acad. Sci. U.S.A. 2019; 116:8310–8319. 30971496 10.1073/pnas.1819682116 PMC6486775

[70] Peccoud J. , Ycart B. Markovian modeling of gene-product synthesis. Theor. Popul. Biol. 1995; 48:222–234.

[71] Golding I. , Paulsson J., Zawilski S.M., Cox E.C. Real-Time kinetics of gene activity in individual bacteria. Cell. 2005; 123:1025–1036. 16360033 10.1016/j.cell.2005.09.031

[72] Miller O.L. Jr , Hamkalo B.A., Thomas C.A. Jr Visualization of bacterial genes in action. Science. 1970; 169:392–395. 4915822 10.1126/science.169.3943.392

[73] Zaslaver A. , Bren A., Ronen M., Itzkovitz S., Kikoin I., Shavit S., Liebermeister W., Surette M.G., Alon U. A comprehensive library of fluorescent transcriptional reporters for Escherichia coli. Nat. Methods. 2006; 3:623–628. 16862137 10.1038/nmeth895

[74] Bar-even A. , Paulsson J., Maheshri N., Carmi M., Shea E.O., Pilpel Y., Barkai N. Noise in protein expression scales with natural protein abundance. Nat. Genet. 2006; 38:636–643. 16715097 10.1038/ng1807

[75] Newman J.R.S. , Ghaemmaghami S., Ihmels J., Breslow D.K., Noble M., DeRisi J.L., Weissman J.S. Single-cell proteomic analysis of S. cerevisiae reveals the architecture of biological noise. Nature. 2006; 441:840–846. 16699522 10.1038/nature04785

[76] Koch A.L. , Levy H.R. Protein turnover in growing cultures of Escherichia coli. J. Biol. Chem. 1955; 217:947–957. 13271454

[77] Ringquist S. , Shinedling S., Barrick D., Green L., Binkley J., Stormo G.D., Gold L. Translation initiation in Escherichia coli: sequences within the ribosome-binding site. Mol. Microbiol. 1992; 6:1219–1229. 1375310 10.1111/j.1365-2958.1992.tb01561.x

[78] Chen H. , Shiroguchi K., Ge H., Xie X.S. Genome-wide study of mRNA degradation and transcript elongation in Escherichia coli. Mol. Syst. Biol. 2015; 11:781. 25583150 10.15252/msb.20145794 PMC4332155

[79] Deutscher M.P. Degradation of RNA in bacteria: comparison of mRNA and stable RNA. Nucleic Acids Res. 2006; 34:659–666. 16452296 10.1093/nar/gkj472 PMC1360286

[80] Roe J.H. , Burgess R.R., Record M.T. Jr Temperature dependence of the rate constants of the Escherichia coli RNA polymerase-lambda PR promoter interaction. Assignment of the kinetic steps corresponding to protein conformational change and DNA opening. J. Mol. Biol. 1985; 184:441–453. 3900414 10.1016/0022-2836(85)90293-1

[81] deHaseth P.L. , Zupancic M.L., Record M.T. Jr RNA polymerase-promoter interactions: the comings and goings of RNA polymerase. J. Bacteriol. 1998; 180:3019–3025. 9620948 10.1128/jb.180.12.3019-3025.1998 PMC107799

[82] Buc H. , McClure W.R. Kinetics of open complex formation between *Escherichia coli* RNA polymerase and the lac UV5 promoter. Evidence for a sequential mechanism involving three steps. Biochemistry. 1985; 24:2712–2723. 3896304 10.1021/bi00332a018

[83] Chong S. , Chen C., Ge H., Xie X.S. Mechanism of transcriptional bursting in bacteria. Cell. 2014; 158:314–326. 25036631 10.1016/j.cell.2014.05.038 PMC4105854

[84] Palma C.S.D. , Kandavalli V., Bahrudeen M.N.M., Minoia M., Chauhan V., Dash S., Ribeiro A.S. Dissecting the in vivo dynamics of transcription locking due to positive supercoiling buildup. Biochim. Biophys. Acta (BBA) - Gene Regul. Mech. 2020; 1863:194515. 10.1016/j.bbagrm.2020.194515 32113983

[85] Nöllmann M. , Stone M.D., Bryant Z., Gore J., Crisona N.J., Hong S.-C., Mitelheiser S., Maxwell A., Bustamante C., Cozzarelli N.R. Multiple modes of Escherichia coli DNA gyrase activity revealed by force and torque. Nat. Struct. Mol. Biol. 2007; 14:264–271. 17334374 10.1038/nsmb1213

[86] Yan Y. , Ding Y., Leng F., Dunlap D., Finzi L. Protein-mediated loops in supercoiled DNA create large topological domains. Nucleic Acids Res. 2018; 46:4417–4424. 29538766 10.1093/nar/gky153 PMC5961096

[87] Dorman C.J. DNA supercoiling and transcription in bacteria: a two-way street. BMC Mol Cell Biol. 2019; 20:26. 31319794 10.1186/s12860-019-0211-6 PMC6639932

[88] Revyakin A. , Ebright R.H., Strick T.R. Promoter unwinding and promoter clearance by RNA polymerase: detection by single-molecule DNA nanomanipulation. Proc. Natl. Acad. Sci. U.S.A. 2004; 101:4776–4780. 15037753 10.1073/pnas.0307241101 PMC387324

[89] Ma J. , Wang M.D. DNA supercoiling during transcription. Biophys. Rev. 2016; 8:75–87. 10.1007/s12551-016-0215-9 PMC5338639 28275417

[90] Gellert M. , O’Dea M.H., Itoh T., Tomizawa J. Novobiocin and coumermycin inhibit DNA supercoiling catalyzed by DNA gyrase. Proc. Natl. Acad. Sci. U.S.A. 1976; 73:4474–4478. 794878 10.1073/pnas.73.12.4474 PMC431506

[91] Mizuuchi K. , O’Dea M.H., Gellert M. DNA gyrase: subunit structure and ATPase activity of the purified enzyme. Proc. Natl. Acad. Sci. U.S.A. 1978; 75:5960–5963. 153529 10.1073/pnas.75.12.5960 PMC393096

[92] Wang X. , Llopis P.M., Rudner D.Z. Organization and segregation of bacterial chromosomes. Nat. Rev. Genet. 2013; 14:191–203. 23400100 10.1038/nrg3375 PMC3869393

[93] Stracy M. , Wollman A.J.M., Kaja E., Gapinski J., Lee J.-E., Leek V.A., McKie S.J., Mitchenall L.A., Maxwell A., Sherratt D.J. et al. Single-molecule imaging of DNA gyrase activity in living Escherichia coli. Nucleic Acids Res. 2019; 47:210–220. 30445553 10.1093/nar/gky1143 PMC6326794

[94] Stracy M. , Lesterlin C., Garza de Leon F., Uphoff S., Zawadzki P., Kapanidis A.N. Live-cell superresolution microscopy reveals the organization of RNA polymerase in the bacterial nucleoid. Proc. Natl. Acad. Sci. U.S.A. 2015; 112:E4390–E4399. 26224838 10.1073/pnas.1507592112 PMC4538611

[95] Stracy M. , Schweizer J., Sherratt D.J., Kapanidis A.N., Uphoff S., Lesterlin C. Transient non-specific DNA binding dominates the target search of bacterial DNA-binding proteins. Mol. Cell. 2021; 81:1499–1514. 33621478 10.1016/j.molcel.2021.01.039 PMC8022225

[96] Uphoff S. , Reyes-Lamothe R., Garza de Leon F., Sherratt D.J., Kapanidis A.N. Single-molecule DNA repair in live bacteria. Proc. Natl. Acad. Sci. U.S.A. 2013; 110:8063–8068. 23630273 10.1073/pnas.1301804110 PMC3657774

[97] Uphoff S. Super-Resolution microscopy and tracking of DNA-Binding proteins in bacterial cells. Methods Mol. Biol. 2016; 1431:221–234. 27283312 10.1007/978-1-4939-3631-1_16 PMC4970795

[98] Zawadzki P. , Stracy M., Ginda K., Zawadzka K., Lesterlin C., Kapanidis A.N., Sherratt D.J. The localization and action of topoisomerase IV in Escherichia coli chromosome segregation is coordinated by the SMC complex, MukBEF. Cell Rep. 2015; 13:2587–2596. 26686641 10.1016/j.celrep.2015.11.034 PMC5061553

[99] Rovinskiy N. , Agbleke A.A., Chesnokova O., Pang Z., Higgins N.P. Rates of gyrase supercoiling and transcription elongation control supercoil density in a bacterial chromosome. PLoS Genet. 2012; 8:e1002845. 22916023 10.1371/journal.pgen.1002845 PMC3420936

[100] Gubaev A. , Weidlich D., Klostermeier D. DNA gyrase with a single catalytic tyrosine can catalyze DNA supercoiling by a nicking-closing mechanism. Nucleic Acids Res. 2016; 44:10354–10366. 27557712 10.1093/nar/gkw740 PMC5137430

[101] Sugino A. , Higgins N.P., Brown P.O., Peebles C.L., Cozzarelli N.R. Energy coupling in DNA gyrase and the mechanism of action of novobiocin. Proc. Natl. Acad. Sci. U.S.A. 1978; 75:4838–4842. 368801 10.1073/pnas.75.10.4838 PMC336216

[102] de Boer H.A. , Bakker A.J., Weyer W.J., Gruber M. The role of energy-generating processes in the degradation of guanosine tetraphosphate, ppGpp, in Escherichia coli. Biochim. Biophys. Acta (BBA) - Nucleic Acids Protein Synth. 1976; 432:361–368. 10.1016/0005-2787(76)90146-5 773436

[103] Malinen A.M. , Bakermans J., Aalto-Setälä E., Blessing M., Bauer D.L.V., Parilova O., Belogurov G.A., Dulin D., Kapanidis A.N. Real-Time single-molecule studies of RNA polymerase-promoter open complex formation reveal substantial heterogeneity along the promoter-opening pathway. J. Mol. Biol. 2022; 434:167383. 34863780 10.1016/j.jmb.2021.167383 PMC8783055

[104] Postow L. , Crisona N.J., Peter B.J., Hardy C.D., Cozzarelli N.R. Topological challenges to DNA replication: conformations at the fork. Proc. Natl. Acad. Sci. U.S.A. 2001; 98:8219–8226. 11459956 10.1073/pnas.111006998 PMC37424

[105] Dillon S.C. , Dorman C.J. Bacterial nucleoid-associated proteins, nucleoid structure and gene expression. Nat. Rev. Microbiol. 2010; 8:185–195. 20140026 10.1038/nrmicro2261

[106] Bryant J.A. , Sellars L.E., Busby S.J.W., Lee D.J. Chromosome position effects on gene expression in Escherichia coli K-12. Nucleic Acids Res. 2014; 42:11383–11392. 25209233 10.1093/nar/gku828 PMC4191405

[107] Sobetzko P. Transcription-coupled DNA supercoiling dictates the chromosomal arrangement of bacterial genes. Nucleic Acids Res. 2016; 44:1514–1524. 26783203 10.1093/nar/gkw007 PMC4770239

[108] Sutormin D. , Rubanova N., Logacheva M., Ghilarov D., Severinov K. Single-nucleotide-resolution mapping of DNA gyrase cleavage sites across the escherichia coli genome. Nucleic Acids Res. 2019; 47:1373–1388. 30517674 10.1093/nar/gky1222 PMC6379681

[109] Hassan S. , Keshavarz-Moore E., Ward J. A cell engineering strategy to enhance supercoiled plasmid DNA production for gene therapy. Biotechnol. Bioeng. 2016; 113:2064–2071. 26928284 10.1002/bit.25971 PMC4982056

[110] Klein C.A. , Teufel M., Weile C.J., Sobetzko P. The bacterial promoter spacer modulates promoter strength and timing by length, TG-motifs and DNA supercoiling sensitivity. Sci. Rep. 2021; 11:24399. 34937877 10.1038/s41598-021-03817-4 PMC8695583

[111] Forquet R. , Pineau M., Nasser W., Reverchon S., Meyer S. Role of the discriminator sequence in the supercoiling sensitivity of bacterial promoters. Msystems. 2021; 6:e0097821. 34427530 10.1128/mSystems.00978-21 PMC8422995

[112] Yeung E. , Dy A.J., Martin K.B., Ng A.H., Del Vecchio D., Beck J.L., Collins J.J., Murray R.M Biophysical constraints arising from compositional context in synthetic gene networks. Cell Syst. 2017; 5:11–24. 28734826 10.1016/j.cels.2017.06.001

[113] Baptista I.S.C. , Kandavalli V., Chauhan V., Bahrudeen M.N.M., Almeida B.L.B., Palma C.S.D., Dash S., Ribeiro A.S. Sequence-dependent model of genes with dual σ factor preference. Biochim. Biophys. Acta Gene Regul. Mech. 2022; 1865:194812. 35338024 10.1016/j.bbagrm.2022.194812

[114] Chauhan V. , Bahrudeen M.N.M., Palma C.S.D., Baptista I.S.C., Almeida B.L.B., Dash S., Kandavalli V., Ribeiro A.S. Analytical kinetic model of native tandem promoters in e. coli. PLoS Comput. Biol. 2022; 18:e1009824. 35100257 10.1371/journal.pcbi.1009824 PMC8830795

[115] Boles T.C. , White J.H., Cozzarelli N.R. Structure of plectonemically supercoiled DNA. J. Mol. Biol. 1990; 213:931–951. 2359128 10.1016/S0022-2836(05)80272-4

