# Supplementary material for: Alteration of DNA supercoiling serves as a trigger of short-term cold shock repressed genes of E. coli
Source: Nucleic Acids Res. 2022 Aug 3;50(15):8512–28. doi: 10.1093/nar/gkac643 (PMC9410904; doi:10.1093/nar/gkac643)
Supplement: gkac643_Supplemental_Files [file gkac643_supplemental_files.zip › Supplementary_Information.pdf]

Supplementary Information for

Alteration of DNA supercoiling serves as a trigger of short-term cold shock repressed genes of *E. coli*

Suchintak Dash[1†*], Cristina S.D. Palma[1†*], Ines S.C. Baptista[1], Bilena L.B. Almeida[1], Mohamed N.M. Bahrudeen[1], Vatsala Chauhan[1], Rahul Jagadeesan[1] and Andre S. Ribeiro[1,2*]

[1] Laboratory of Biosystem Dynamics, Faculty of Medicine and Health Technology, Tampere University, Tampere, 33520, Finland.

[2] Center of Technology and Systems (CTS-Uninova), NOVA University of Lisbon, 2829-516 Monte de Caparica, Portugal.

[†] Equal contributions.

*To whom correspondence should be addressed. E-mail: andre.sanchesribeiro@tuni.fi.
Correspondence may also be addressed to Suchintak Dash, e-mail: suchintak.dash@tuni.fi and Cristina S.D. Palma, e-mail: cristina.santosdiaspalma@tuni.fi.
Present Address: Andre S. Ribeiro, Arvo Ylpön katu 34, 33520, Tampere University, Finland.

Additional Supplementary files:

Supplementary File X1. Statistics of single-cell distributions of protein fluorescence of the genes measured by flow cytometry in optimal, cold shock and gyrase inhibition conditions. (Excel)

Supplementary File X2. Transcription factor network of cold shock repressed genes. (Excel)

Supplementary File X3. List of biological processes of cold shock repressed genes. (Excel)

Supplementary Methods

I. RNA-Seq experiments and analysis

I.a) Sample preparation

We shifted the temperature for cells in the mid-exponential growth phase (OD_{600} = 0.3) to 15 °C (here named minute 0) and collected cells from three independent colonies, as well as after 20, 80, and, 180 minutes, respectively. The same was done with cell colonies not subjected to the temperature shift, for purposes of control. Meanwhile, when subjecting cells to the antibiotic novobiocin (50 µg/mL), the cells were only collected 120 minutes after the treatment, to reduce cell-to-cell diversity due to e.g., different absorption times, as in (1).

After collecting the samples, 5 mL of the culture was immediately treated with a double volume (10 mL) of RNA protect bacteria reagent (Qiagen, Germany) for 5 minutes at room temperature, to prevent RNA degradation. Next, treated cells were pelleted and frozen at -80 °C overnight. The next morning, total RNA was extracted using the RNeasy kit (Qiagen, Germany).

I.b) Sequencing

Extracted RNA was treated twice with DNase (Turbo DNA-free kit, Ambion, USA) and quantified using Qubit 2.0 Fluorometer RNA assay (Invitrogen, Carlsbad, CA, USA). The total RNA quality was determined using a 1% agarose gel stained with SYBR Safe (Invitrogen, Carlsbad, CA, USA), where RNA was detected using UV in a Chemidoc XRS imager (Biorad, USA). RNA integrity was measured by the Agilent 4200 TapeStation (Agilent Technologies, Palo Alto, CA, USA).

RNA library preparations, sequencing, and quality control analysis of sequenced data were conducted at GENEWIZ, Inc. (Leipzig, Germany). In detail, ribosomal RNA depletion was performed using Ribo-Zero Gold Kits (Bacteria probe) (Illumina, San Diego, CA, USA), while the RNA sequencing library was prepared using the NEBNext Ultra RNA Library Prep Kit.

The sequencing libraries were multiplexed and clustered on one lane of a Flowcell, which was loaded on an Illumina HiSeq 4000 instrument (after cold shock) or on an Illumina NovaSeq 6000 instrument (after gyrase inhibition). In both instruments, the samples were sequenced using a single-index 2x150 Paired-End (PE) configuration. Image analysis and base calling were conducted by the HiSeq Control Software (Illumina HiSeq) and by the NovaSeq Control Software v1.7 (Illumina NovaSeq). The raw sequence data (.bcl files) was converted into "fastq" files and de-multiplexed using Illumina bsl2fastq v.2.20. One mismatch was allowed for index sequence identification.

I.c) RNA-seq data analysis pipeline

i) RNA sequencing reads were trimmed to remove possible adapter sequences and nucleotides with poor quality using *Trimmomatic* v.0.36 (2). ii) Trimmed reads were mapped to the reference genome, *E. coli* MG1655 (NC_000913.3), using the STAR aligner v.2.5.2b (after cold shock) or the *Bowtie2*

aligner v.2.3.5.1 (after gyrase inhibition), generating BAM files (3, 4). iii) Unique gene hit counts were calculated with *featureCounts* from the *Rsubread* R package (v.1.34.7) (5). Genes with less than 5 counts in more than 3 samples, and genes whose mean counts were smaller than 10 were removed from further analysis. iv) The read counts were used for downstream differential expression analysis. The *DESeq2* R package (v.1.24.0) (6) was used to calculate $\log_2$ of fold changes (LFC) of RNA levels between group of samples and calculate p-values using Wald tests (function *nbinomWaldTest*). We also calculated relative abundances of mRNA in a given condition using the Transcripts Per Million (TPM) normalization (7).

Finally, the sequencing platforms for the cold shock experiment and for the novobiocin experiment differ for logistical reasons (similarly, the aligners differ). Consequently, we avoided quantitative comparisons between genes responses (e.g., between specific numbers). Our conclusions are based on qualitative comparisons (i.e., we checked if the changes in the two perturbations are linearly correlated or not).

The RNA-seq on cold shock responses informs on 4328 genes, while the RNA-seq on novobiocin responses informs on 3948 genes.

II. Flow cytometry and data analysis

We measured cells fluorescence using an ACEA NovoCyte Flow Cytometer (ACEA Biosciences Inc., San Diego, USA). Cells were diluted (1:10000) into 1 mL of phosphate buffer saline (PBS) solution, vortexed for 10 seconds. In each condition, 3 biological replicates were obtained. In each replicate, we collected data from 50,000 cells. The flow rate was set to 14 µL/minute. The data was collected by the Novo Express software (ACEA Biosciences Inc.).

For detecting YFP, we used a blue laser (488 nm) for excitation and the fluorescein isothiocyanate detection channel (FITC-H) (530/30 nm filter) for emission, with a core diameter of 7.7 μM and a PMT voltage of 600. For detecting mCherry, we used the PE-Texas Red fluorescence detection channel (615/20 nm) for emission, with a PMT voltage of 584.

The lower bound for the detection threshold in FSC-H was set to 5000 to remove interference from particles. We also removed the 1% highest FITC-H values. Further, to remove abnormal cells, we then used an iterative procedure to discard outliers, which are those points whose vertical distance from the best-fit function is larger than 2 (8). The process always converged in 1 to 2 iterations. Finally, we searched for additional abnormal measurements at the single gene level in the 3 repeats, but we did not find any.

III. Microscopy and image analysis

Cells were pelleted and re-suspended in ~100 µL of the remaining media. Three microliters of cell suspension were placed on a 2% agarose gel pad made up of M9 medium and kept in between the round microscope slide and a coverslip. It took ~5 min to move cells from the incubator to the microscope and start the observation. This includes the assembly of the microscope imaging chamber

containing the slides and cells. Cells were visualized by confocal microscopy with a 100x objective. Phase-contrast images were taken by an external phase-contrast system. YFP tagged strain was visualized by a 488 nm laser and a 514/30 emission filter, while DAPI stained nucleoids were visualized by a 405 nm laser and a 447/60 emission filter. Phase contrast and confocal images were taken simultaneously.

Microscopy images were analysed automatically by the ‘CellAging’ software (9), followed by the ‘SCIP’ software (10). First, cell borders were automatically segmented from phase contrast images using CellAging. This software then aligns, also automatically, the results of the cell segmentation with the corresponding fluorescence images. Next, ‘SCIP’ (10) was used to segment the spatial fluorescence distributions from DAPI-stained nucleoids in single cells. Both software based their methods on Gradient Path Labelling (11).

Specifically, e.g., to segment nucleoids, SCIP creates an initial segmentation seed per nucleoid from the image intensity gradients. Next, it segments each nucleoid using the seed as the centre of a two-dimensional Gaussian. The intensity of the neighbouring pixels of the seeds are used to create the gaussian profile in each direction. Finally, this profile is transformed into a binary mask using a threshold applied on the profile (10). Examples of cell and nucleoid segmentations are shown in Supplementary Figure S3.

Nucleoid segmentation is used to estimate the fluorescence from DNA binding proteins inside the nucleoid region, relative to the total cell florescence. Notably, the ratios of fluorescence intensities here reported ranged from 55% to 65% (Figures 7B and 7C). Past works described stronger overlaps (e.g. (12)). The most likely explanation for this is our use of automatic nucleoid segmentation, which allowed collecting data from several hundred cells per condition, consistently and reproducibly. For this, we set a threshold to differentiate between “high” (inside the nucleoid) versus “low” (outside the nucleoid) fluorescence intensity regions. Consequently, the nucleoid region will be smaller than if the criterion was the existence of any fluorescence in the pixels of that region (which would not occur due to cell autofluorescence). This latter criterion was not used since it can introduce errors if implemented automatically. This methodological difference should not affect our qualitative conclusion of whether the engagement of gyrase/RpoB with the nucleoid changes significantly, or not, during cold shock.

IV. Gene ontology (GO)

To study the GO representation (13, 14) of cold shock repressed genes, we performed an overrepresentation test using the PANTHER Classification System (15). This test finds statistically significant overrepresentations using Fisher’s exact test, which rejects the null hypothesis that there are no associations between the genes’ cohort and the corresponding GO of the biological process for p-values < 0.05. This p-value is corrected for the False Discovery Rate (FDR) using the Benjamini-Hochberg procedure (16).

V. Gene fitness

From 4133 reference bacterial DNAs with listed genes (17), we used the Rentrez package (18) to count in how many of these genomes one finds each gene of MG1655 (GCF_000005845.2_ASM584v2). We use these numbers (divided by the total number of genomes) as a measure of the evolutionary fitness of each gene, which in bacteria can propagate in the biosphere by cell division or by horizontal gene transfer. In detail, we calculated the mean and CV^2 of gene fitness of cold shock repressed genes as well as for all genes in the genome, along with their standard errors using bootstrapping (10^4 resampling with replacement). For mixed genes cohort, we calculated the same along with their standard errors using bootstrapping (10^4 resampling with replacement) but here each sample consists of genes from different groups of samples of different sizes.

VI: Analysis of the AT and CG content of the promoters

From Regulon DB, we obtained the lists of all 8791 promoters and of all 3700 transcription units (TUs) (19). We then filtered the promoter list to contain only the 2355 promoters associated to TUs, and subsequently discarded 93 promoters with unknown sequence. The resulting list was comprised of 2262 promoters, each with a sequence spanning from 60 nucleotides upstream the transcription start site to 20 nucleotides downstream (i.e., from positions -60 to +20, with the transcription start site assumed to be in the position +1).

Next, for each promoter, we extracted the sequences from positions -60 to -35, positions -35 to -10, and positions -10 to +1 (the -35 to -10 being the sequence that most influence the RNAP binding (20), while the others were used for comparison). Similarly, we also extracted 443 promoter sequences of cold shock repressed genes. Finally, for each set of sequences, we calculated the fractions of A, C, G and T. Finally, the AT (or GC) content of each promoter was calculated by summing the fractions of A and T (or C and G, respectively).

VII. Correction for cellular auto-fluorescence in flow cytometry data

When assessing the single-cell distributions of protein expression levels measured by flow cytometry, we corrected for the cell auto-fluorescence (21, 22). For this, we first measured by flow cytometry the auto-fluorescence of control cells (i.e., absent of YFP fusions). Next, we corrected the mean fluorescence measured by flow cytometer by applying equation VII.1 (22):

$$M_p = M_T - M_{cell} \qquad \text{(VII.1)}$$

Here, M_p is the mean cell fluorescence due to YFP presence alone, after subtracting the cell auto-fluorescence. Meanwhile, M_T is the mean cell fluorescence measured by flow cytometry, while

M_{cell} is the mean cell auto-fluorescence. Similarly, to correct the variance σ^2, we apply equation VII.2 (22):

$$\sigma_p^2 = \sigma_T^2 - \sigma_{cell}^2 \qquad \text{(VII.2)}$$

From equations VII.1 and VII.2 one can derive equation VII.3, to estimate the corrected squared coefficient of variation, CV_p^2, of the single-cell distribution of protein expression levels:

$$CV_p^2 = \left(\frac{\sigma_p}{M_p} \right)^2 \qquad \text{(VII.3)}$$

For correcting the skewness (S) of the distribution we apply equation (VII.4) as in (21):

$$S_p = \frac{S_T \cdot \sigma_T{}^3 - S_{cell} \cdot \sigma_{cell}{}^3}{\sigma_p{}^3} \qquad \text{(VII.4)}$$

VIII: Correlation between RNA and protein numbers

RNA and protein numbers are expected to be positively correlated in bacteria, since transcription and translation are mechanically bound (23–25) and because most gene expression regulation occurs during transcription initiation (26).

To assess if this holds true during cold shock, we searched for correlations between LFC's (Supplementary Section I), as measured by RNA-seq at 20 and 80 min after the temperature shift, and the corresponding LFC's in protein numbers, measured by flow cytometry (Supplementary Section II) at 120 and 180 min after the temperature shift. The lag of 100 minutes between RNA and protein measurements should suffice for changes in numbers of the former to propagate to the latter. The list of genes tested is shown in Supplementary Table S2. Results in Supplementary Figure S13B show that changes in RNA and protein numbers are correlated during cold shock.

IX: Promoter sequence logos

Promoter sequence logos were created using WebLogo (27). From positions -25 to -1 of each promoter, it counts in how many promoters is each nucleotide present. Then, it piles up the nucleotides (A, C, T, G), sorted from the rarest in the bottom to the most frequent in the top.

The height of each nucleotide letter in the plot, in each position, equals the frequency multiplied by the total information at that position. That total information is quantified by the difference between the maximum uncertainty at any position ($\log_2(n)$, where n = 4 is the number of possible nucleotides) and the uncertainty given the frequencies found, also quantified by Shannon's information:

$\log_2 n - \sum_{i=1}^{4} f_i \times \log_2(f_i)$. Given this, we expect that DNA with more conserved positions will have more 'bits' (28).

X: P-distances

We calculated the p-distance between a promoter sequence and the consensus sequence (sequence composed of the most common nucleotide for each position of the sequence). The p-distance is the fraction of nucleotides of the promoter sequence that differ from the consensus sequence. Thus, it ranges from [0,1], where 0 corresponds to identical sequences and 1 to sequences whose nucleotides differ in every position. For genes with more than one promoter, we obtained the average of the p-distance of each promoter.

XI: Ribosome binding site and start codon sequences of cold shock repressed genes

RNA translation rates are controlled by the rate at which ribosomes are recruited to the ribosome binding site region of the RNA, along with the rate at which they then initiate translation. The recruitment rate differs with the ribosome binding site sequence (29) and the genome wide consensus sequence of ribosome binding sites is "5'-AGGAGG-3'", being named the Shine-Dalgarno sequence (30).

Meanwhile, the rate of translation initiation is influenced by the start codon upstream the ribosome binding site. In *E. coli*, 83% of the start codons have the sequence AUG (3542/4284), 14% (612) the sequence GUG, 3% (103) the sequence UUG (31) and a couple the sequence AUU (32, 33).

We obtained the mean and standard deviation of the distributions of the p-distances (Supplementary Section X) of the RNAs coded by cold shock repressed genes to the Shine-Dalgarno sequences (Table S10) and to each of the 4 start codons sequences (Table S11) and studied if they differ significantly from the mean and standard deviation of p-distances of the genome wide distribution. Finally, since the distance (in number of nucleotides) between the Shine-Dalgarno sequence and the start codon can affect translation initiation rates (30), we also compared them as above.

From Supplementary Figure S20, not only the consensus levels of the two cohorts are the same (bit values of 0.5 between positions -10 and -15 and 1 in the region -1 to -3), but the distances between them are, in both cohorts, 5 nucleotides.

To support, we also compared the sequence logos of the 25 nucleotides upstream of the start codons (34) (Supplementary Figure S21) of cold shock repressed genes and the genome wide distribution. Again, we find little differences between the logos in Supplementary Figures S20A and S20B.

XII: Estimation of the average transcription rate of cold shock repressed **genes during optimal growth conditions**

From (35), the mean RNA numbers (as measured by FISH) of a cold shock repressed gene during optimal growth is 0.35 per cell. Given the 1-step model (Model 1.1 in Figure 6), which is applied during optimal growth, the mean number of RNAs per cell in steady state is given by:

$$k_1 = M_{RNA} \times \lambda_1 \tag{XII.1}$$

Assuming λ_1=0.004 s^{-1} (36, 37), k_1 is estimated to be 1.4×10^{-3} s^{-1}.

XIII. Robustness of the single-cell measurements of protein fluorescence levels

The robustness of the single-cell expression levels measured using the YFP-fusion library (35), was assessed by performing similar measurements using instead a GFP-promoter-fusion library (38). Comparisons at the promoter level (Supplementary Figure S14) show linear correlations in the mean single-cell fluorescence at both temperatures, as well as high R^2 (> 0.75), from which we conclude that measurements using cells of the YFP-fusion library are, in general, robust qualitatively. Note that, first, since in the YFP strain library the target gene is chromosome-integrated while in the GFP promoter fusion library the target gene is on a low-copy plasmid, the mean expression levels are lower using the YFP library, and for this and other reasons (e.g., different dynamics of supercoiling buildup in plasmids (1, 39) we did not confront their CV^2 values of single-cell fluorescence levels.

XIV. Derivations

XIV.a. Squared coefficient of variation and skewness assuming a Γ distribution of single-cell protein numbers

From (35), most single-cell distributions of protein numbers in *E. coli* are well described by a Γ distribution. If that holds, the first three moments of single-cell distributions of protein numbers should be given by:

$$M = k\theta \tag{XIVa.1}$$

$$\sigma^2 = k\theta^2 \tag{XIVa.2}$$

$$S = \frac{2}{\sqrt{k}} \tag{XIVa.3}$$

where M, σ^2, S, k and θ are the mean, variance, skewness, shape parameter, and scale parameter of a Γ distribution, respectively. From equations XIVa.1 and XIVa.2:

$$CV^2 = \frac{\sigma^2}{M^2} = \frac{\theta}{M} \tag{XIVa.4}$$

This relationship was empirically validated in (8, 35). Finally, from equation XIVa.1 and XIVa.3:

$$S = \frac{2}{\sqrt{k}} = \frac{2}{\sqrt{\frac{M}{\theta}}} = \frac{2}{\sqrt{M}} \cdot \sqrt{\theta} \qquad \text{(XIVa.5)}$$

XIV.b Derivation of Ω assuming the 1-step model

The steady state solutions for mean RNA and protein numbers, assuming the one step model (reactions 1.1, 2, 3, and 4 in Figure 6A), are given by, respectively (35):

$$M_{RNA} = \frac{k_{1.1}}{\lambda_1} \qquad \text{(XIVb.1)}$$

$$M_P = \frac{M_{RNA} \cdot k_2}{\lambda_2} = \frac{k_{1.1} \cdot k_2}{\lambda_1 \cdot \lambda_2} \qquad \text{(XIVb.2)}$$

Meanwhile, the variance of the single-cell protein numbers is given by (35):

$$\sigma_{\mathrm{P}}^2 = \frac{k_{1.1} \cdot k_2}{\lambda_1 \cdot \lambda_2} \cdot \left(1 + \frac{k_2}{\lambda_1 + \lambda_2}\right) \qquad \text{(XIVb.3)}$$

From (XIVb.2) and (XIVb.3):

$$CV_p{}^2 = \frac{\lambda_1 \cdot \lambda_2}{k_{1.1} \cdot k_2} \cdot \left(1 + \frac{k_2}{\lambda_1 + \lambda_2}\right) = \frac{1}{\mathrm{M_P}} \cdot \left(1 + \frac{k_2}{\lambda_1 + \lambda_2}\right) \qquad \text{(XIVb.4)}$$

Here, we define the constant Ω as:

$$\Omega = \left(1 + \frac{k_2}{\lambda_1 + \lambda_2}\right) \qquad \text{(XIVb.5)}$$

This result is in line with past results in (8). Also, given (XIVa.4), we expect $\theta = \Omega$. As such, we refer to this constant as Ω.

XIV.c Derivation of the squared coefficient of variation assuming the 1-step model

Assuming Model 1.1 in Figure 6, from (XIVb.2), and (XIVb.4):

$$CV_p{}^2 = \frac{1}{\frac{k_1 \cdot k_2}{\lambda_1 \cdot \lambda_2}} \cdot \left(1 + \frac{k_2}{\lambda_1 + \lambda_2}\right) \qquad \text{(XIVc.1)}$$

XIV.d Derivation of the squared coefficient of variation assuming the ON-OFF model

Assuming the ON-OFF model (Model 1.3 in Figure 6), the mean RNA and protein numbers in single cells at steady state are, respectively:

$$M_{RNA} = \frac{k_+}{k_+ + k_-} \cdot \frac{k_{1.3}}{\lambda_1} \qquad \text{(XIVd.1)}$$

$$M_P = \frac{M_{RNA} \cdot k_2}{\lambda_2} = \frac{k_+}{k_+ + k_-} \cdot \frac{k_{1.3} \cdot k_2}{\lambda_1 \cdot \lambda_2} \qquad \text{(XIVd.2)}$$

Meanwhile, the variance is (35):

$$\sigma_{\mathrm{P}}^2 = \frac{k_+}{k_+ + k_-} \cdot \frac{k_{1.3} \cdot k_2}{\lambda_1 \cdot \lambda_2} \cdot \left(1 + \frac{k_2}{\lambda_1 + \lambda_2}\left(1 + \left(1 - \frac{k_+}{k_+ + k_-}\right)\frac{k_{1.3}(k_+ + k_- + \lambda_1 + \lambda_2)}{(k_+ + k_- + \lambda_1)(k_+ + k_- + \lambda_2)}\right)\right) \qquad \text{(XIVd.3)}$$

From equations XIVd.2 and XIVd.3:

$$CV_P^2 = \frac{1}{M_P} \cdot \left(1 + \frac{k_2}{\lambda_1 + \lambda_2}\left(1 + \left(1 - \frac{k_+}{k_+ + k_-}\right)\frac{k_{1.3}(k_+ + k_- + \lambda_1 + \lambda_2)}{(k_+ + k_- + \lambda_1)(k_+ + k_- + \lambda_2)}\right)\right) \qquad \text{(XIVd.4)}$$

From XIVd.4 and XIVa.4, Ω should equal:

$$\Omega = \left(1 + \frac{k_2}{\lambda_1 + \lambda_2}\left(1 + \left(1 - \frac{k_+}{k_+ + k_-}\right)\frac{k_{1.3}(k_+ + k_- + \lambda_1 + \lambda_2)}{(k_+ + k_- + \lambda_1)(k_+ + k_- + \lambda_2)}\right)\right) \qquad \text{(XIVd.5)}$$

XIV.e Changes in Ω due to cold shock

To estimate the change in Ω due to shifting to cold shock we considered that, prior to cold shock, the cold shock responsive genes are not significantly affected by locking due to supercoiling sensitivity (since they are relatively highly expressing in optimal growth conditions). As such, their dynamics in optimal conditions should be well modeled by Model 1.1 in Figure 6A in the main manuscript. On the other hand, when subject to cold shock, we expect that frequent locking due to supercoiling sensitivity will be the main responsible for their negative response. Thus, the appropriate model during cold shock should be Model 1.3 in Figure 6A in the main manuscript.

Thus, given the results in Supplementary Sections XIV.b and XIV.d, the change in Ω when shifting to cold shock (CS), relative to the control (CTRL) condition, should equal:

$$\frac{\Omega_{CS}}{\Omega_{CTRL}} = \frac{\left(1 + \frac{k_2^{CS}}{\lambda_1^{CS} + \lambda_2^{CS}}\left(1 + \left(1 - \frac{k_+}{k_+ + k_-}\right)\frac{k_1((k_+ + k_-) + \lambda_1^{CS} + \lambda_2^{CS})}{((k_+ + k_-) + \lambda_1^{CS})((k_+ + k_-) + \lambda_2^{CS})}\right)\right)}{\left(1 + \frac{k_2^{CTRL}}{\lambda_1^{CTRL} + \lambda_2^{CTRL}}\right)} \qquad \text{(XIVe.1)}$$

Next, consider that, from Table S12, $\frac{k_2}{\lambda_1+\lambda_2}$ is much larger than 1. Thus, for simplicity, we replace $\left(\frac{k_2}{\lambda_1+\lambda_2}+1\right)$ by $\frac{k_2}{\lambda_1+\lambda_2}$. Further, we consider that, in *E. coli*, RNA degradation rates are much higher than protein degradation rates, i.e., $\lambda_1 >> \lambda_2$ (Table S12). Given this, we also replace $(\lambda_1+\lambda_2)$ by λ_1. Consequently:

$$\frac{\Omega_{CS}}{\Omega_{CTRL}} = \frac{k_2^{CS} \times \lambda_1^{CTRL}}{k_2^{CTRL} \times \lambda_1^{CS}} \cdot \left(1+\left(1-\frac{k_+}{k_+ + k_-}\right)\frac{k_1((k_+ + k_-)+\lambda_1^{CS}+\lambda_2^{CS})}{((k_+ + k_-)+\lambda_1^{CS})((k_+ + k_-)+\lambda_2^{CS})}\right) \quad \text{(XIVe.2)}$$

This, can be simplified to:

$$\Leftrightarrow \frac{\Omega_{CS}}{\Omega_{CTRL}} = \frac{k_2^{CS} \times \lambda_1^{CTRL}}{k_2^{CTRL} \times \lambda_1^{CS}} \cdot \left(1+\left(\frac{k_1 . k_-}{(k_+ + k_-)^2}\right)\right) \quad \text{(XIVe.3)}$$

We note that we expect that the term $\left(\frac{k_1 . k_-}{(k_+ + k_-)^2}\right)$ is the one containing the most temperature sensitive rate constants, given that the one-step model in Figure 6A includes the other rates constants (k_2 and λ_1) and could not explain the dynamics following cold shock for the reasons listed in Section '*An ON-OFF model can explain the short-term dynamics of cold shock repressed genes'* in the main manuscript.

XV. Estimation of a lower bound of skewness

Supplementary Figure S19, informs on a lower bound for noise (CV^2 ~ 0.38) since noise and mean are no longer correlated below that value. Based on this lower bound, we estimated a lower bound of skewness. From the equations in Table S9, one can write:

$$S = 2 \cdot CV \quad \text{(XV.1)}$$

We estimate a lower bound for the skewness to equal ~1.23, in agreement with the data (Figure 5), in that below that value, the predicted and empirical skewness do not correlate.

XVI: Effects of cell division on Ω

At temperatures above cold shock (30 °C, 25 °C and 20 °C), the cells exhibited significant doubling times (Figure 2A). Meanwhile, at cold shock they did not divide (Figure 2A).

Cell division can affect single-cell variability in protein numbers, provided asymmetries in the partitioning of RNA and protein numbers between sister cells (for a review, see (40)). This difference between the conditions, could affect the comparison of the contribution of noise in gene expression in optimal and cold shock conditions.

We thus estimated the effects of cell division on Ω at temperatures above cold shock (30 °C, 25 °C and 20 °C) if the division rate at 30 °C, 20 °C and 25 °C was null.

Since we are considering temperatures above cold shock (30 °C, 25 °C and 20 °C), we assume the 1-step model (Model 1.1 in Figure 6A). Next, we assume that $\lambda_1 >> \lambda_2$, since, in general RNA degrades much faster than proteins (35). Given this, from Equation XIVb.5 in Supplementary Section XIVb:

$$\frac{\Omega_{(\lambda_d = 7\times10^{-5})}}{\Omega_{(\lambda_d = 0)}} = \frac{\left(1 + \frac{k_2}{(\lambda_1 + \lambda_d) + (\lambda_2 + \lambda_d)}\right)}{\left(1 + \frac{k_2}{\lambda_1 + \lambda_2}\right)} = \frac{\left(\frac{k_2}{(\lambda_1 + \lambda_d) + (\lambda_d)}\right)}{\left(\frac{k_2}{\lambda_1}\right)} = \frac{\lambda_1}{\lambda_1 + 2\lambda_d} \qquad \text{(XVI.1)}$$

Next, from (36), we assume that, on average λ_1=0.004 s^{-1}. Also, we measured the mean cell division rate at 30 °C-20 °C to be 241 min^{-1} (Figure 2A). This inverse should correspond to the protein and RNA dilution rates due to cell division and it equals: λ_d =7×10^{-5} s^{-1}. As such:

$$\frac{\Omega_{(\lambda_d = 7\times10^{-5})}}{\Omega_{(\lambda_d = 0)}} = 0.97 \qquad \text{(XVI.2)}$$

Given this, if cells were not dividing in optimal conditions, Ω would be 3% higher. This is within the 90% confidence interval of the green line in Figure 4B. Thus, we do not include cell division in the models.

XVII. Comparison to a past study on the genome wide effects of antibiotics targeting gyrase

One study (41) also identified which genes have high supercoiling sensitivity by subjecting cells to antibiotics targeting gyrase (novobiocin and norfloxacin) and performing microarrays at 2, 5, 10, and 20 min after introducing the antibiotics. Of the 306 genes classified in (41) as supercoiling sensitive, 289 were measured in our RNA-seq, but only 23 were classified as cold shock repressed. Nevertheless, this is higher than expected by chance (p-value < 0.05). Moreover, of the 381 genes here classified as cold shock repressed, 251 are reported in (41). Their response 20 min after gyrase inhibition is stronger (|LFC| = 0.74) than expected by chance (p-value < 0.05). We conclude that there is weak, but non-negligible agreement between ours and the data in (41). Relevantly, our measurements differ significantly in timing following the addition of the antibiotics. Potentially, lesser differences may increase the agreement.

The first p-value was obtained by calculating the probability of 2 random sets of size 289 and 381 (each set was randomly sampled without replacement from 4003 and 4328 genes, respectively) having an interception of more than 23 elements (10^5 runs). The second p-value was obtained by calculating the probability of random sets of size 251 (each set was randomly sampled from 4003

elements, without replacement) having an average |LFC| > 0.74. The probability was calculated based on 10^5 runs.

XVIII. Responses to novobiocin cannot be explained by transcription factor interactions, closely spaced promoters, or (p)ppGpp sensitivity

We tested if the single gene responses to novobiocin ($|LFC_{Novo}|$) could be explained by TF regulation, including global TF regulators (information from RegulonDB). Specifically, we confronted $|LFC_{Novo}|$ of each input TF and each of its output genes. We found that, on average, they do not correlate statistically (Supplementary Figure S25A). Similarly, we did not find a correlation between strongly supercoiling sensitive genes and their input TFs (Supplementary Figure S25B). Finally, we failed to find a correlation between global TF regulators (Supplementary Table S4) and their output genes that are also strongly supercoiling sensitive (Supplementary Figure S25C). Overall, the single gene responses to novobiocin cannot be explained by TFs regulation.

Next, we considered closely spaced promoters, since past works suggest that their dynamics can be influenced by supercoiling (42). In *E. coli*, of the 3948 genes in our RNA-seq data following the addition of novobiocin, 285 genes are controlled by closely spaced promoters (defined in the main manuscript, Section '*Short-term responses of cold shock repressed genes cannot be explained by transcription factor interactions, AT richness, or closely spaced promoters*'). Of these, 97 are strongly supercoiling sensitive genes. These 97 genes have 1.2 times more chances to be regulated by a closely spaced promoter than non-strongly supercoiling sensitive genes. However, this difference is not statistically significant (Fisher test p-value > 0.05). Therefore, closely spaced promoters are not likely to contribute to strong supercoiling sensitivity.

Finally, we considered that (p)ppGpp is known to affect bacterial tolerance to some antibiotics (43). Also, during starvation, which increases (p)ppGpp levels, the DNA supercoiling of plasmids decreases (44). We therefore tested if atypical sensitivity to (p)ppGpp could explain, at least partially, the responsiveness of some genes to novobiocin.

First, we compared the $|LFC_{Novo}|$ of the 1215 here classified as strongly supercoiling sensitive and of the 1139 (p)ppGpp sensitive genes present in our novobiocin RNA-seq data (classification from (45)). A two-sample t-test resulted in a p-value < 0.05, thus, and since the numbers of genes of each of the two classes are similar, we conclude that the two distributions of single-gene responses to novobiocin differ in mean (0.83 and 0.45, respectively), suggesting that (p)ppGpp sensitivity does not explain the genes' strong supercoiling sensitivity.

We further considered that being strongly supercoiling sensitive correlates to being cold shock repressed (Figure 6C). Thus, if (p)ppGppp sensitivity was a common cause for being cold shock repressed, then the probability of genes having the three features should be relatively high. However, from Supplementary Figure S11B, only 51 genes are simultaneously strongly supercoiling sensitive, cold shock repressed, and ppGpp sensitive. This is lower than expected by chance. Specifically, the odds of a gene, that is both cold shock repressed and strongly supercoiling sensitive, being also

(p)ppGpp sensitive is only 0.6 (Fisher's exact test). I.e., it has less chances of being (p)ppGpp sensitive than if it was a non-strongly supercoiling sensitive and cold shock repressed gene.

Overall, the sensitivity to (p)ppGpp is not likely to influence the single-gene responses to novobiocin.

XIX. Null models and statistical tests

In general, to create null-models of how variable X affects variable Y, we performed random sampling without replacement of both *X* and *Y* datapoints. The number of samplings and the sampling size (number of samples in each sampling) are set to the maximum array size allowed by MATLAB (~45980 × 45980, 15.8GB). The number of samplings (*K*) is set to 100 and the sampling size is set according to *Max_size*/*K* where *Max_size* = 45980/2. Next, for both *X* and *Y*, we combine the sampled datapoints in a vector (*sample_X*, *sample_Y*) and calculate the correlation between *sample_X* and *sample_Y* by linear regression fitting using Ordinary Least Squares (OLS).

We obtained a p-value of the fitted regression lines from t-tests with the null hypothesis that the line is horizontal.

We further evaluated the null hypothesis that slopes and intercepts of the best fitting lines of empirical and null-model data are equal. We performed the ANCOVA test (46), which evaluates the significance of an F-test under the null hypothesis that both slopes and intercepts are equal. To correct for over-representation of datapoints in these tests, we corrected the degrees of freedom to be (*size_XY* – *C*), where *size_XY* is the number of datapoints and *C* is the number of parameters. For the linear regression fitting, *C* equals to 2 (intercept and slope of best fitting line). For the ANCOVA test, *C* equals to 4 (intercept and slope of one best fitting line and the difference of these between both best-fitting lines).

XX: Quantifying ATP using spectrophotometry

To quantify ATP levels inside cells, we use the method in (47). First, the total cell fluorescence at excitation wavelength λ is given by:

$$F_\lambda = F_\lambda^m + f_\lambda^{bg} \cdot C + f_\lambda^p . C \qquad \text{(XX.1)}$$

F stands for total fluorescence and *C* for the number of cells. Meanwhile, F^m stands for media fluorescence, f^{bg} for single-cell fluorescence background and f^p for single-cell protein fluorescence (in our case, λ equals 400 nm in one case and 494 nm in the other).

Meanwhile, the total cell fluorescence (without ATP sensors) is:

$$F_\lambda^c = F_\lambda^m + f_\lambda^{bg} \cdot C \qquad \text{(XX.2)}$$

The subtraction of (XX.1) from (XX.2) corrects for media and cell background autofluorescence:

$$F_{\lambda} - F_{\lambda}^{c} = f_{\lambda}^{p}.C \qquad \text{(XX.3)}$$

Given this, the fluorescence from ATP from a cell is estimated by:

$$\frac{f_{\lambda=494}^{p}(t)}{f_{\lambda=400}^{p}(t)} = \frac{F_{\lambda=494}(t) - F_{\lambda=494}^{c}(t)}{F_{\lambda=400}(t) - F_{\lambda=400}^{c}(t)} \qquad \text{(XX.4)}$$

Supplementary Figures

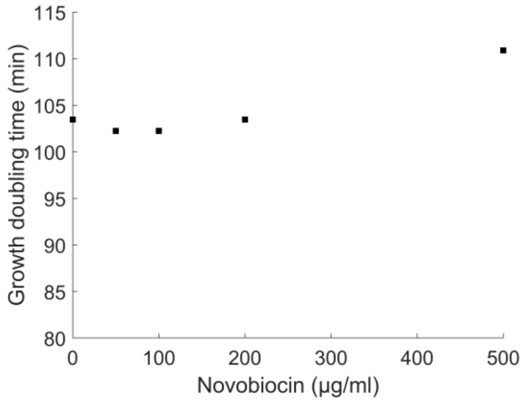

Figure S1: *E. coli* K-12 MG1655 cell growth doubling time versus various concentrations of novobiocin in M9 medium supplemented with 0.4% glucose, amino acids, and vitamins at 30 ºC. The doubling time was calculated by the initial OD_{600nm} value, the final $OD_{600\ nm}$ value and the time interval in between (48). Related with Section '*Bacterial strains, growth conditions, and gene expression measurements'*.

of main manuscript.

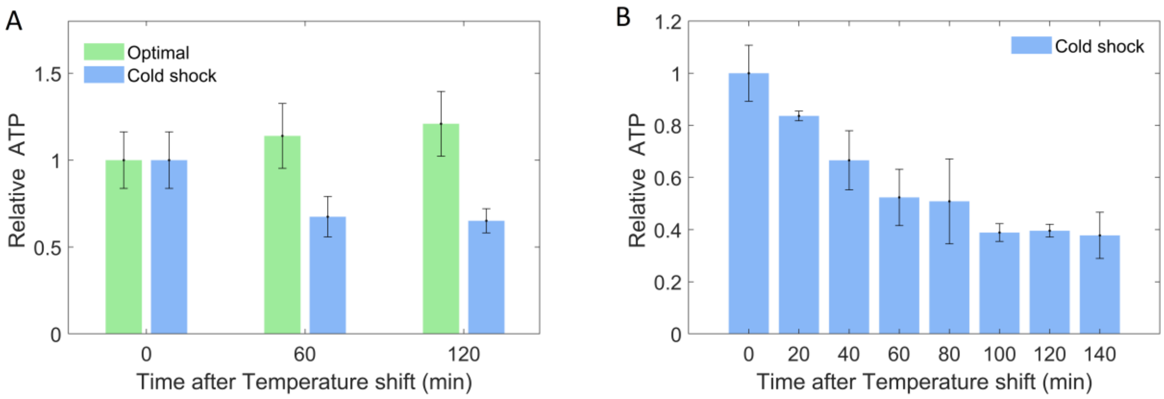

Figure S2: Relative ATP levels measured (Methods Section '*Cellular ATP levels*') using the QUEEN-2m sensor (47). **(A)** Measurements every hour for two hours, with 6 replicates per condition. **(B)** Measurements every 20 min for 140 min, with 3 replicates per condition. Detailed description of correction for autofluorescence and cell division is in (Supplementary Section XX). Related with Section '*Cellular energy levels decrease during cold shock'* of main manuscript.

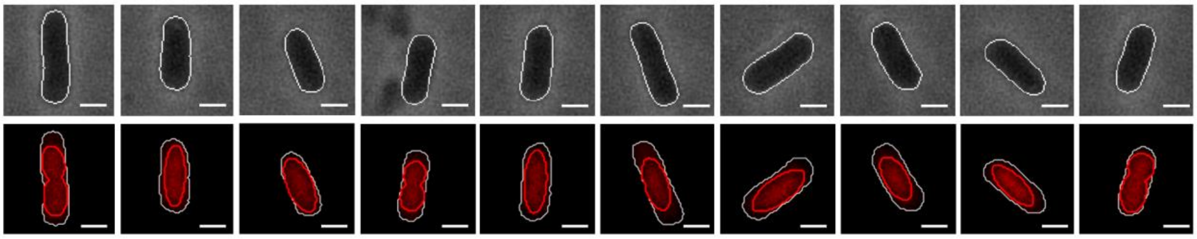

Figure S3: Example cells imaged by phase contrast (top row) and by confocal microscopy (bottom row). The cells were subject to nucleoid DAPI-staining (red dots). Shown is the automatic segmentation of the cell borders (white lines, top row) and of the nucleoids (red lines, bottom row), using the methods described in Supplementary Section III. Also shown in the bottom row are the masks (white lines) extracted from the cell segmentation results (the masks differ from the borders by a few pixels). The nucleoid segmentation results were not manually corrected as they also aim to illustrate that, in general, manual correction was not necessary. These cell images were extracted from the full-sized phase contrast and corresponding confocal microscopy images. As such, the centers and the scale bar lengths of top and bottom images do not necessarily match. Scale bars correspond to 1 µm. Related with Section *'The engagement between gyrases and nucleoid increases during cold shock'* of main manuscript.

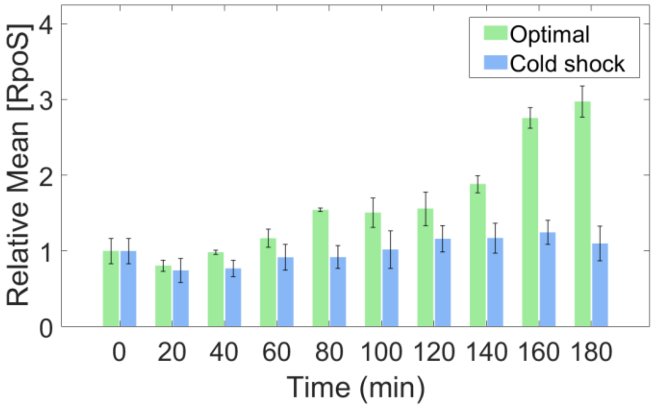

Figure S4: Mean concentration of RpoS (σ^{38}), relative to 0 min, measured every 20 min in both optimal (30 °C) and cold temperature (15 °C), by flow cytometry. Related with Section '*Cell morphology, physiology, and master transcription regulators during cold shock*' of main manuscript.

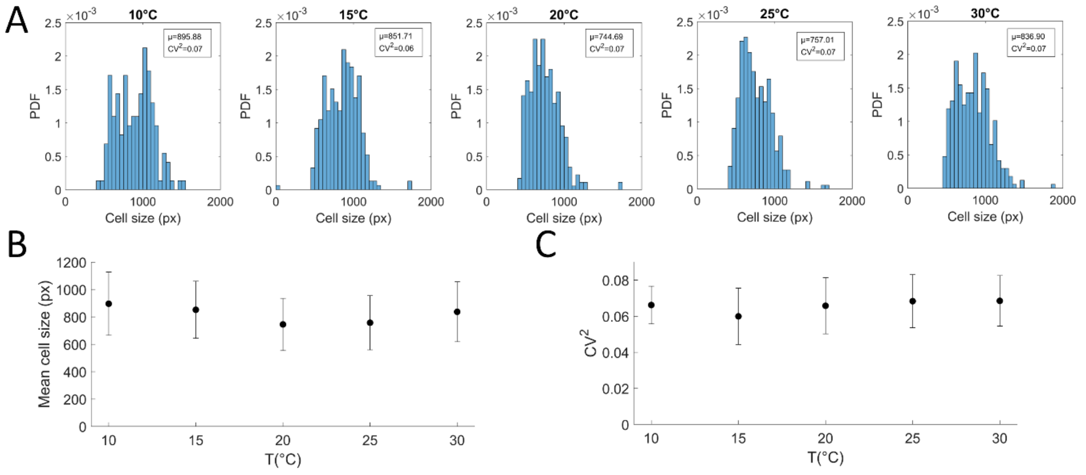

Figure S5: Microscopy measurements of cell size (px) of *E. coli* cells expressing the manY gene endogenously tagged with YFP coding sequence at different temperatures. **(A)** Probability density function (PDF) of the distribution of cell size, 180 min after the temperature shift. **(B)** Mean cell size (px) as a function of temperature. Vertical error bars correspond to the standard deviation. **(C)** Squared coefficient of variation as a function of temperature. Vertical error bars were calculated by bootstrapping (standard deviation of 500 resamples of 20 cells each). Related with Section '*Cell morphology, physiology, and master transcription regulators during cold shock*' of main manuscript.

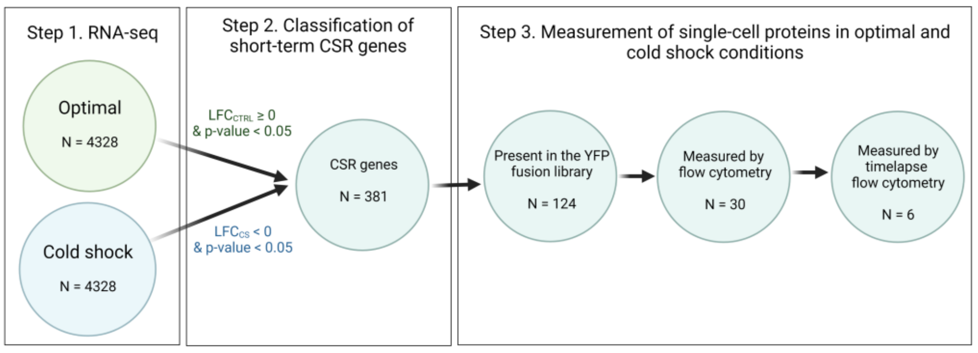

Figure S6: Filtering process for the identification of short-term cold shock repressed (CSR) genes. **(Step 1)** From RNA-seq measurements (Supplementary Section I) under cold shock and optimal temperature (control). **(Step 2)** We classified genes as 'CSR' if their $LFC_{CS} < 0$ (and p-value < 0.05) and their corresponding $LFC_{CTRL} \geq 0$ (and p-value < 0.05). We found that 381 genes respected these conditions (Supplementary File X2). **(Step 3)** Out of the 381 genes, we found 124 in the YFP fusion library (35). Next, we selected 30 genes with high protein expression levels (out of the 124 genes) and measured their single-cell protein expression dynamics by flow cytometry in optimal and cold shock conditions (Supplementary file X1). Finally, we selected 6 of those 30 genes, and further performed time-lapse flow cytometry measurements (Supplementary file X1). These 6 genes have mean protein levels that cover the state space of *M* of the 30 CSR genes. Related with Section '*Identification of short-term cold shock repressed genes'* of main manuscript and with Supplementary Section II.

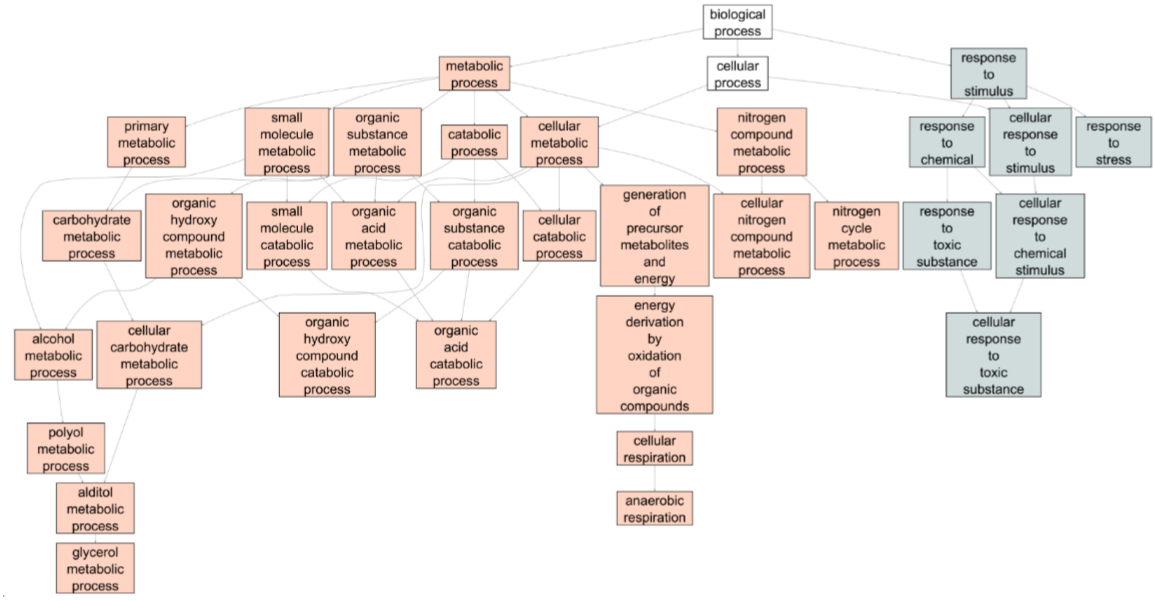

Figure S7: Graph of overrepresented gene ontology terms and their ancestors for cold shock repressed (CSR) genes (Supplementary Section IV). The more general biological processes are connected to specific ontology terms by an arrow pointing to the latter. The ontologies that have the ancestor "metabolic process" and the ones with the ancestor "response to stimulus" are coloured differently. Related with Section '*Ontology and evolutionary fitness of short-term cold shock repressed genes*' of main manuscript.

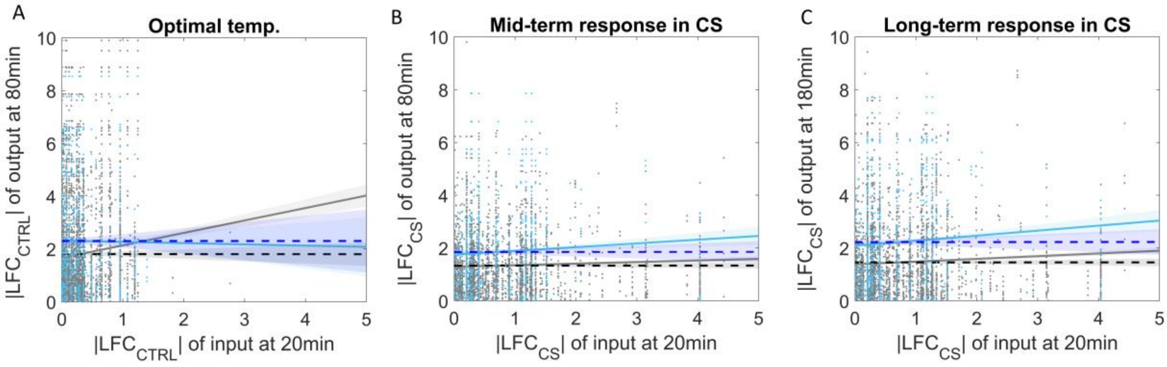

Figure S8: Signal propagation in optimal and cold shock (CS) conditions. **(A)** Scatter plot between $|LFC_{CTRL}|$ of TF output genes at 80 min and corresponding input TF genes at 20 min in optimal conditions. **(B)** and **(C)** Scatter plots between $|LFC_{CS}|$ of output genes at 80 min and 180 min, respectively, and the corresponding $|LFC_{CS}|$ of TF input genes at 20 min during CS. Grey circles are all pairs of genes (4435 pairs). Blue circles (733) are of pairs of input-output TF genes for which the output is a cold shock repressed (CSR) gene. We fitted by OLS regression the best-fit line to the grey and to the blue dots (grey and blue lines, respectively). Dashed lines are the null models (Supplementary Section XIX). For each best fit line (grey and blue line) and correspondent null model (black and dark blue dashed line, respectively), we performed an ANCOVA test with the null hypothesis that the two lines are not statistical distinguishable. P-values < 0.05 reject the null hypothesis. P-values for each test are in Supplementary Tables S6 and Table S8, respectively. Shadow regions are 68% confidence bounds. Related with Section '*Short-term responses of cold shock repressed genes cannot be explained by transcription factor interactions, AT richness, or closely spaced promoters*' of main manuscript.

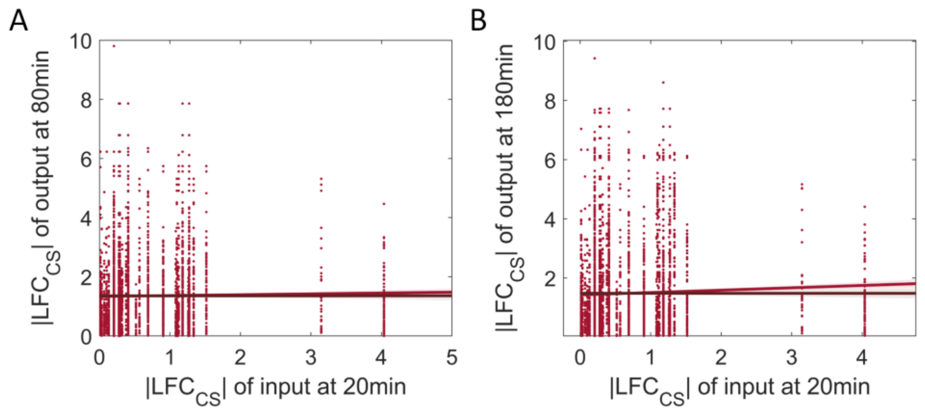

Figure S9: Correlation plots of the |LFC_{CS}| between input and output genes where the input is a global TF regulator (Supplementary Table S4), during cold shock (CS). **(A)** Correlation during cold shock between output genes at 80 min and input genes at 20 min for pairs of output-input genes where the input is a global TF regulator (red circles). **(B)** Correlation during cold shock between output genes at 180min and input genes at 20 min for pairs of output-input genes where the input is a global TF regulator (red circles). To the red circles, we fitted by OLS regression the best fit line. Null models were generated as described in Supplementary Section XIX. For each time point, we did an ANCOVA test with the null hypothesis that the red and dark red lines are not statistically distinguishable. P-values are presented in Supplementary Table S7. Shadow areas are 68% confidence bounds. Related with Section '*Short-term responses of cold shock repressed genes cannot be explained by transcription factor interactions, AT richness, or closely spaced promoters*' of main manuscript.

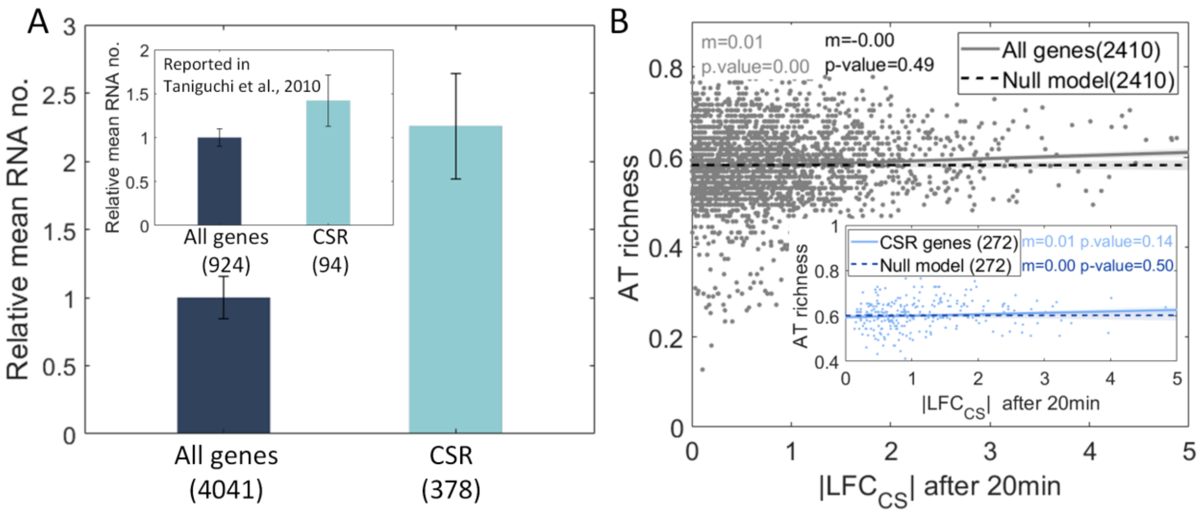

Figure S10: RNA expression level of cold shock repressed (CSR) genes in optimal conditions and their AT promoter richness as a function of response to cold shock. **(A)** Relative RNA expression levels (calculated using TPM normalization) of all genes and of the CSR genes at optimal temperature. The data used to generate the inset figure is from (35). **(B)** Correlation between the genes' AT richness and their response to cold shock ($|LFC_{CS}|$) 20 min after cold shock. The inset shows the same plot when considering only cold shock repressed genes. Related with Section 'Short-term responses of cold shock repressed genes cannot be explained by transcription factor interactions, AT richness, or closely spaced promoters' of main manuscript and Supplementary Section VI.

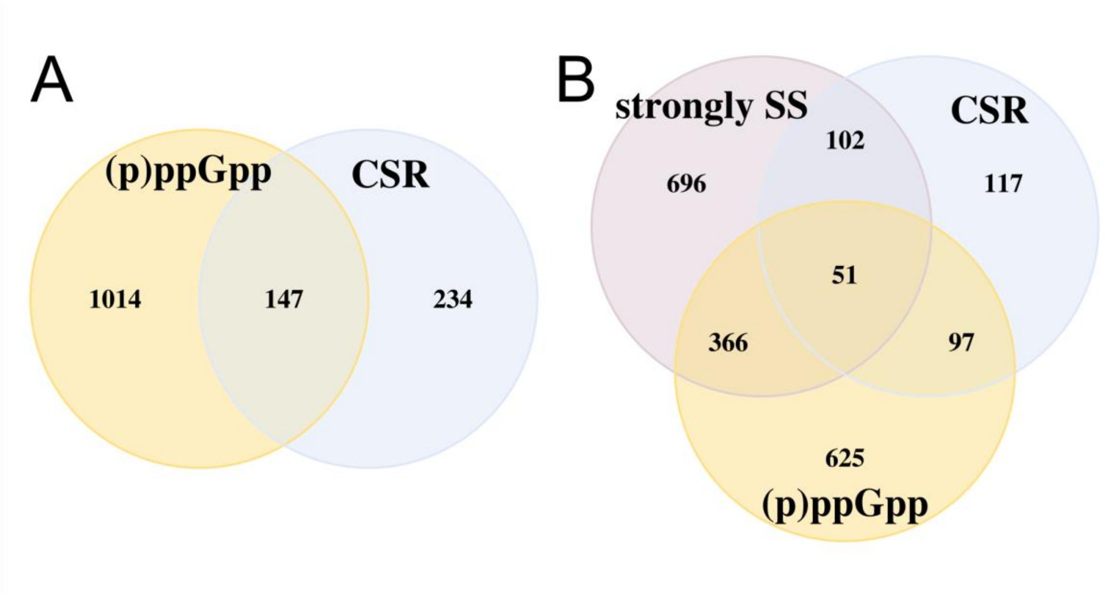

Figure S11: Venn diagram of the number of genes that are cold shock repressed (CSR), responsive to (p)ppGpp, and strongly supercoiling sensitive (SS). **(A)** Venn diagram of overlapping genes with common features of being CSR and (p)ppGpp sensitive (45) present in the RNAseq data during cold shock. Related with Section '*Short-term responses of cold shock repressed genes can be partially explained by operon organization and by (p)ppGpp sensitivity*' of main manuscript. **(B)** Venn diagram of overlapping genes with common features of being CSR, strongly SS, and (p)ppGpp sensitive (45) present in the novobiocin RNAseq data. Related with Section '*Short-term responses of cold shock repressed genes can be partially explained by operon organization and by (p)ppGpp sensitivity*' and '*Response strength to cold shock is correlated with reduced levels of negative supercoiling*' of main manuscript, respectively and to Supplementary Section XVIII.

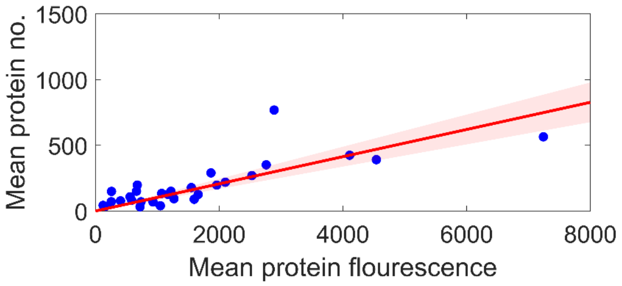

Figure S12: Measured mean protein fluorescence (Supplementary Section II) plotted against the corresponding men protein numbers reported in (35) in the same growth conditions. The best 1st order polynomial fitting line of the type *y=mx* has a statistically significant slope *m* = 0.10 and a $R^2 = 0.59$. We performed an F-test on the regression model, which tests for the null hypothesis that a 1st order polynomial does not significantly improve the fitting compared to a 0-order polynomial. The test rejected the null hypothesis (p-value < 0.05). Related with Section '*The scaling between noise and mean of single-cell cold shock repressed protein numbers is temperature dependent*' of main manuscript.

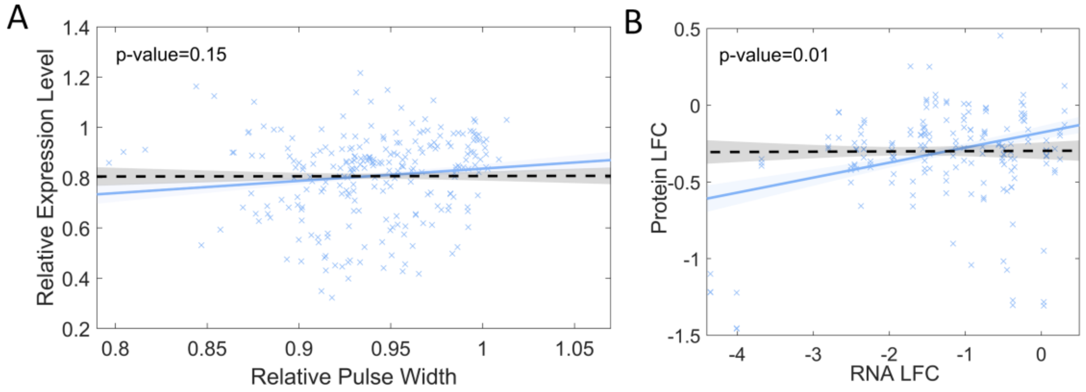

Figure S13: Correlation between relative protein expression levels, RNA expression levels and cell size. **(A)** Correlation plot between the mean protein expression levels and the mean pulse width, of 6 genes (aldA, feoA, manY, ndk, pepN, tktB) measured by flow cytometer every 20 min for 140 min (Supplementary Section II). The *X* and *Y* axis values are relative to the pulse width and the mean protein expression level at 0 min, respectively. Black dashed line is the null model (Supplementary Section XIX), the two lines are not statistical distinguishable (p-value = 0.15). **(B)** Correlation plot of the $|LFC_{CS}|$ of RNAs (measured by RNAseq at 15 °C, at time 20 min and 80 min after the temperature shift) and protein expression levels (measured by flow cytometry at 15 °C, 120 min and 180 min after (with a gap of 100 min (49)) the temperature shift). Black dashed line is the null model, the two lines are statistical distinguishable (p-value = 0.01). This is consistent with the cell division times (~140 min in M9 media). As such, we expect phenotypic consequences from the cold shock response at the RNA level to be propagated into the proteins produced in the same period (50–52). Related with Section '*The scaling between noise and mean of single-cell cold shock repressed protein numbers is temperature dependent*' of main manuscript.

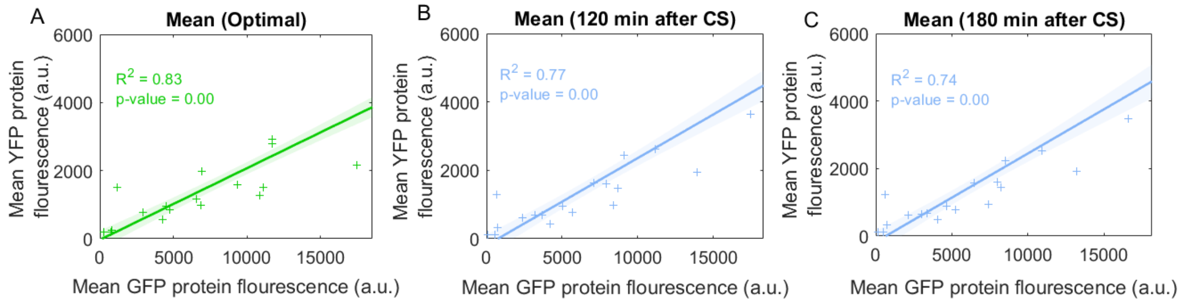

Figure S14: Scatter plots of the mean single-cell expression levels of 19 cold shock (CS) repressed promoters using a promoter-GFP fusion library (38) and a YFP strain library (28). Data shown are from the **(A)** optimal condition, and **(B)** 120 min and **(C)** 180 min after CS, respectively. Related with Section '*The scaling between noise and mean of single-cell cold shock repressed protein numbers is temperature dependent*' of main manuscript and Supplementary Section XIII.

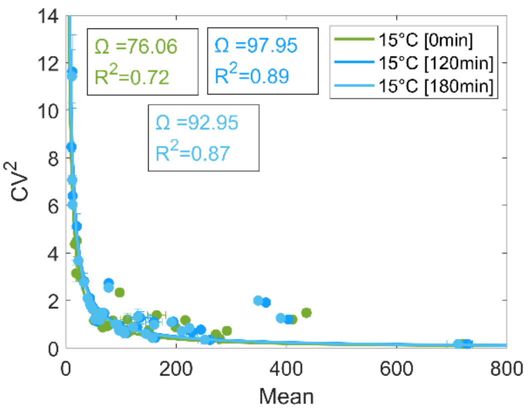

Figure S15: Squared coefficient of variation (CV^2) as a function of mean protein fluorescence level. measured by the FITC-H channel of the flow cytometer at 15 °C, 0 min, 120 min and 180 min. To the data we fit the best-fit function $CV^2 = \Omega/M$ (8, 35), where Ω is a constant (values for each curve are shown in the insets) and *M* is the mean protein number. We performed a 2-sample t-test to check for the null hypothesis that there is no difference between the Ω values at 120 min and 180 min. The test does not reject the null hypothesis with a p-value of 0.43. Related with Section '*The scaling between noise and mean of single-cell cold shock repressed protein numbers is temperature dependent*' of main manuscript.

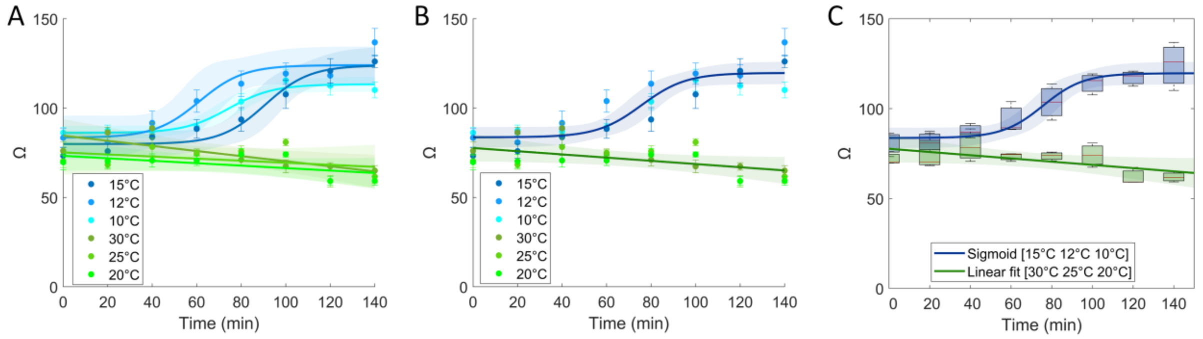

Figure S16: Relationship between Ω and time for optimal and cold shock conditions **(A)** For each temperature we fit the best fitting function. Data >= 20 °C is best fit by a 1st order polynomial while data < 20 °C is best fit by a sigmoid function (function given by $\frac{L}{1+e^{-0.1\cdot(x-x_0)}}$, where *L* is the curve's maximum value and x_0 is the x value of the sigmoid midpoint). **(B)** Average best fitting functions for data < 20 °C and >= 20 °C. The dark blue and green lines correspond to the best fitting curves (that maximize R^2), for the 15 °C - 10 °C dataset and for the 30 °C - 20 °C dataset, respectively. **(C)** Box plot of Ω as a function of time for two temperature sets (control and cold shock). The red line in the box is the median The distance between the bottom and top of each box is the interquartile range. The vertical black bars are the range between the minimum and maximum value at each moment. For control and cold shock temperatures, we fit the best fitting function. Related with Section '*The scaling between noise and mean of single-cell cold shock repressed protein numbers is temperature dependent*' of main manuscript.

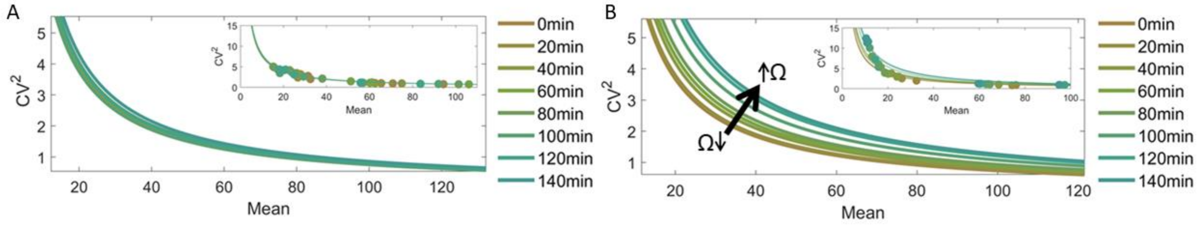

Figure S17: Squared coefficient of variation (CV^2) as a function of mean protein numbers measured by flow cytometer during **(A)** optimal and **(B)** cold shock temperatures, every 20 min for 140 min, respectively. To each time moment we fit the function $CV^2 = \Omega / M$ (8, 35). Fittings are shown for each time moment. Each time moment data comes from triplicates from 6 genes (aldA, feoA, many, ndk, pepN, tktB). **(A-B inset)**. Data points along with best-fit curves are shown. Related with Section '*The scaling between noise and mean of single-cell cold shock repressed protein numbers is temperature dependent*' of main manuscript.

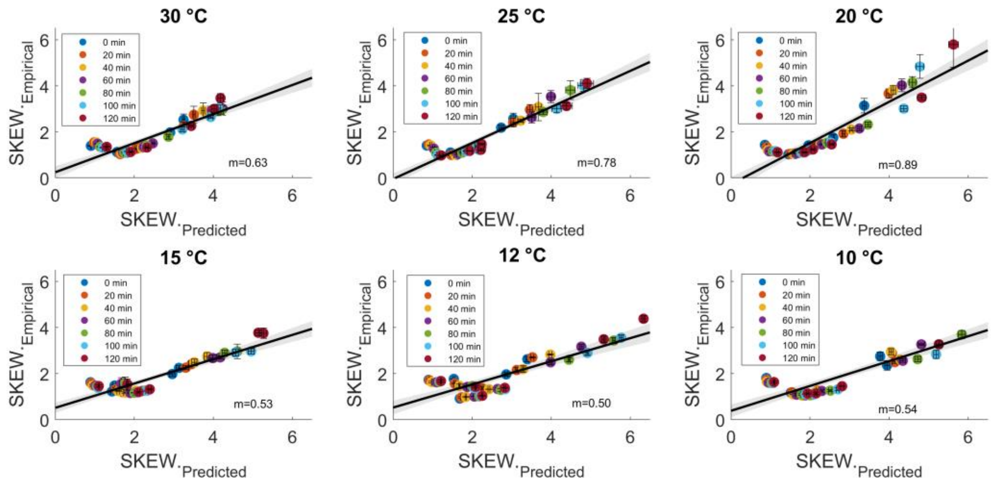

Figure S18: Correlation between empirical and predicted skewness (SKEW.) for 30° C, 25 °C, 20 °C, 15 °C, 12 °C, 10 °C. Predicted skewness is estimated from the Ω estimated from the relationship between CV^2 and M (Supplementary Section XIV.a) from empirical flow cytometry data while the empirical skewness is the skewness of the empirical distributions obtained by flow cytometry. For all fittings R^2 is > 0.8. Related with Section '*The scaling between noise and mean of single-cell cold shock repressed protein numbers is temperature dependent*' of main manuscript.

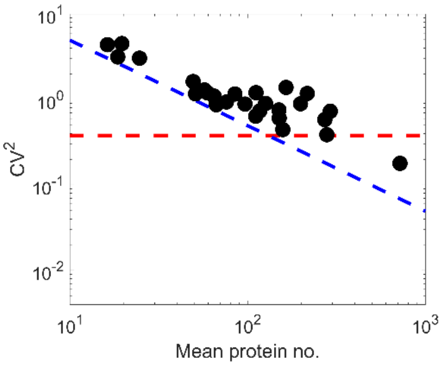

Figure S19: Squared coefficient of variation (CV^2) versus mean protein numbers. For mean protein number < 150 the protein expression noise is inversely proportional to the mean with a lower noise limit (blue dashed line), corresponding to intrinsic noise (35). For mean protein number >150 noise becomes independent of the mean with a lower bound of ~0.38 (red dashed line). Related with Section '*The scaling between noise and mean of single-cell cold shock repressed protein numbers is temperature dependent*' of main manuscript and Supplementary Section XV.

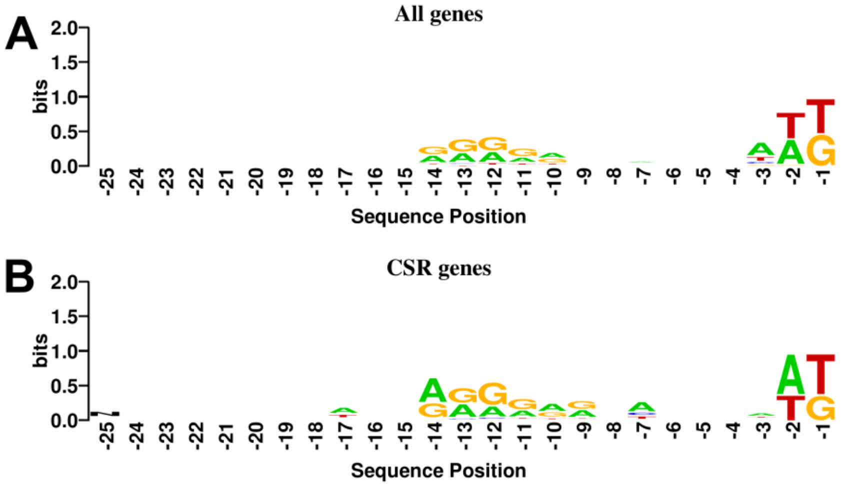

Figure S20: Sequence logos of the ribosome binding site sequence from the Shine-Dalgarno to the start codon for **(A)** 179 genes and **(B)** the 31 cold shock repressed genes of those 179. Data from RegulonDB. The last nucleotide of the start codon is placed in the position -1. Also, the nucleotides positions decrease in the upstream direction. Related with Section '*An ON-OFF model can explain the short-term dynamics of cold shock repressed genes*' of main manuscript and Supplementary Sections IX, X and XI.

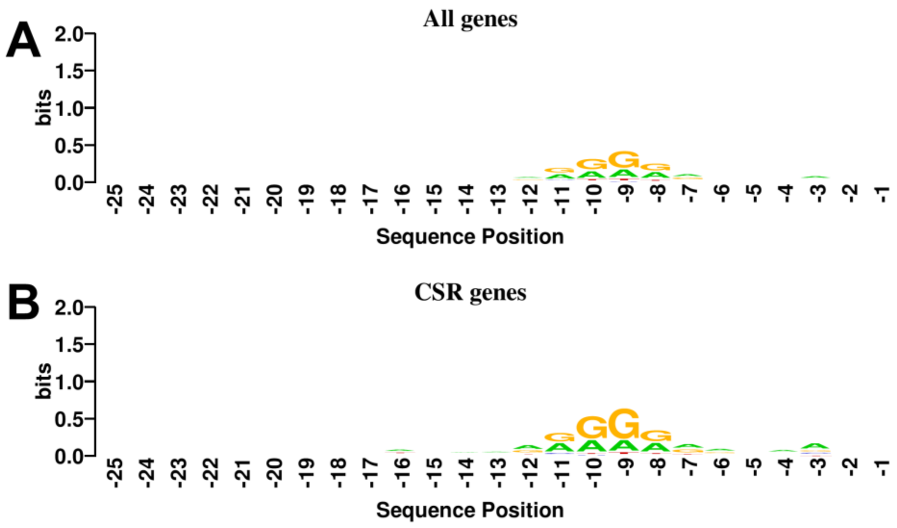

Figure S21: Sequence logo of the ribosome binding site sequence ranging from the Shine-Dalgarno sequence to the start codon **(A)** for all 4357 genes and **(B)** for 377 cold shock repressed (CSR) genes. The nucleotide upstream of the start codon of the gene is assumed to have the position -1 and the position of a nucleotide decreases as it is located more upstream. Related with Section ‘*An ON-OFF model can explain the short-term dynamics of cold shock repressed genes*’ of main manuscript and Supplementary Sections IX, X and XI.

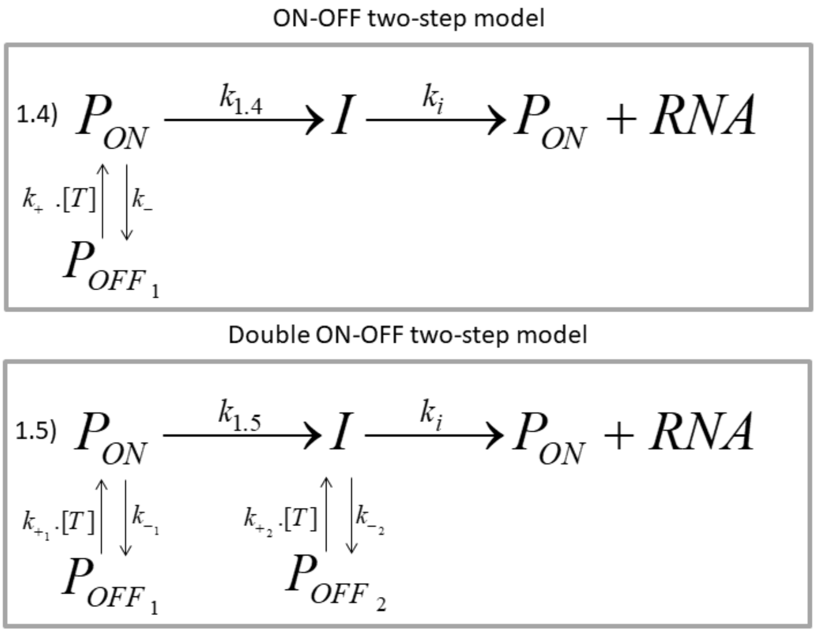

Figure S22: Kinetic models involving ON-OFF in either one (1.4) or two (1.5) rate-limiting steps in transcription initiation, while having the same reactions for translation and RNA and protein decay (reactions 2, 3, and 4, respectively in Figure 6A). Related with Section ‘*An ON-OFF model can explain the short-term dynamics of cold shock repressed genes*’ of main manuscript.

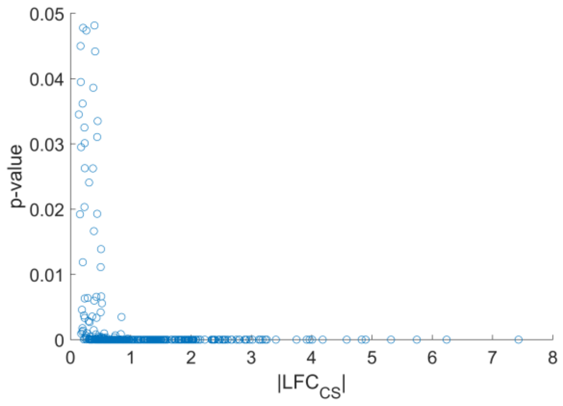

Figure S23: Scatter plot between |LFCcs| and p-values of the cold shock repressed genes from the RNA-seq data. Related with Section '*Response strength to cold shock is correlated with reduced levels of negative supercoiling*' of main manuscript.

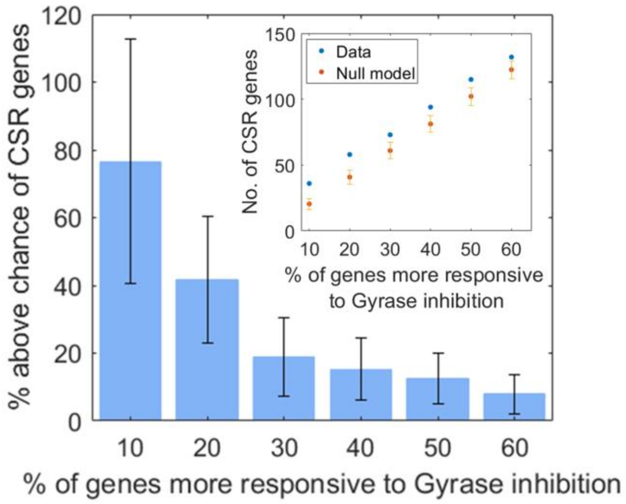

Figure S24: Percentage above random chance that a gene that is strongly supercoiling sensitive (in that it is strongly responsive to gyrase) is also cold shock repressed (CSR). Results are shown assuming different thresholds for defining a gene as strongly supercoiling sensitive, given a total of 1626 genes classified as being supercoiling sensitive. The inset shows the number of CSR genes (out of 381), out of the cohort of the (10% to 60%) most responsive genes to gyrase inhibition (1626 genes in total for a p-value < 0.05). For the null model, for each data point, we randomly selected, 1000 times, a cohort from the supercoiling sensitive responsive genes with the same size as the correspondent percentage of most responsive supercoiling sensitive genes. The orange data points correspond to the number of genes of the random cohorts that are also in the CSR cohort. Vertical error bars correspond to the standard error of the mean. Related with Section '*Response strength to cold shock is correlated with reduced levels of negative supercoiling*' of main manuscript.

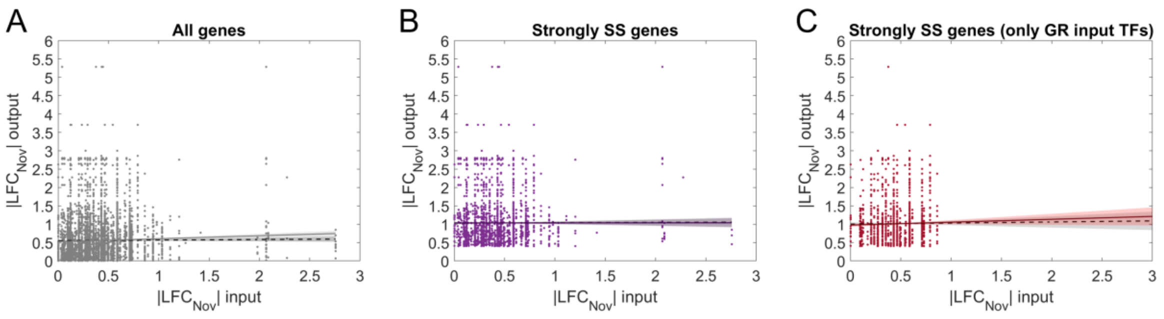

Figure S25: Signal propagation after subjecting cells to novobiocin **(A)** Correlation plots of $|LFC_{Novo}|$ of each output gene with the $|LFC_{Novo}|$ of the direct input TFs when subjected to novobiocin. **(B)** Correlation plots of $|LFC_{Novo}|$ of strongly supercoiling sensitive (SS) genes with the $|LFC_{Novo}|$ of their direct input TFs. **(C)** Correlation plots of $|LFC_{Novo}|$ of strongly SS genes with the $|LFC_{Novo}|$ of their direct input TFs. Here, only global regulators are considered as input TFs (listed in Supplementary Table S4). In all cases, we fitted the lines by OLS regression. Null models (black lines) were generated as described in Supplementary Section XIX. For each case, we performed an ANCOVA test with the null hypothesis that the best-fit and null model lines are not statistically distinguishable. In no case was the null hypothesis rejected (p-values in Supplementary Table S7). Shadow areas are 68% confidence bounds. Related with Section '*Response strength to cold shock is correlated with reduced levels of negative supercoiling*' of main manuscript and to Supplementary Section XVIII.

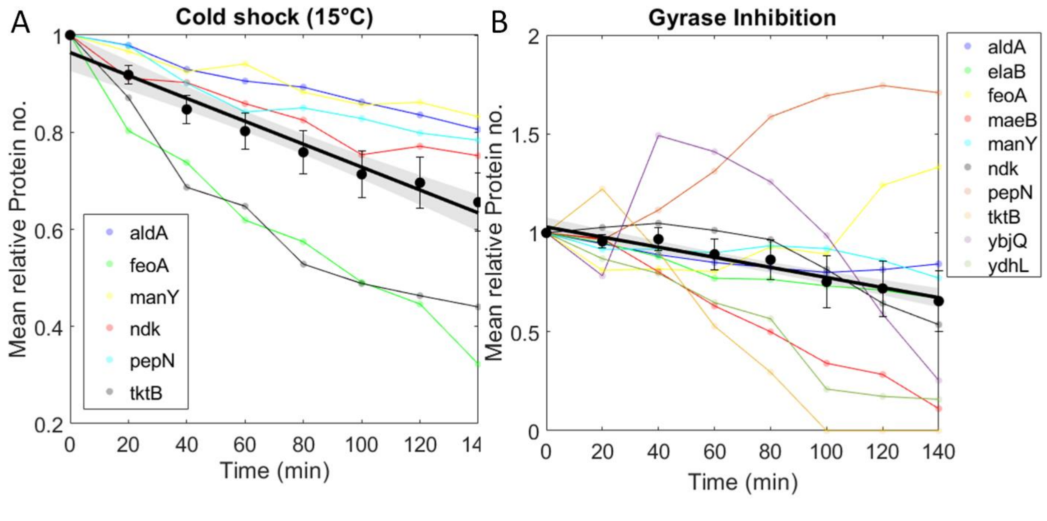

Figure S26: Mean protein numbers as a function of time after the perturbation. (**A**) Change in relative mean protein numbers of 6 cold shock repressed genes over time following cold shock. **(B)** Change in relative mean protein numbers of 10 cold shock repressed genes over time following gyrase inhibition by novobiocin. Related with Section '*Response strength to cold shock is correlated with reduced levels of negative supercoiling*' of main manuscript.

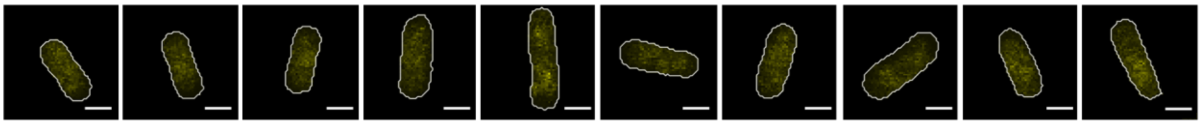

Figure S27: Example confocal microscopy images of cells expressing GyrA-YFP (yellow dots). Also shown are the pixel masks (white lines) obtained by automatic segmentation of the cell borders, using the method described in Supplementary Section III. Scale bars correspond to 1 µm. Related with Section '*The engagement between gyrases and nucleoid increases during cold shock*' of main manuscript.

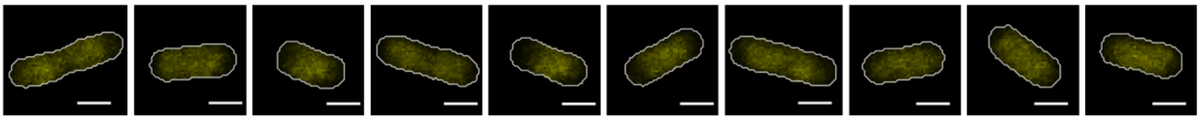

Figure S28: Example confocal microscopy images of cells expressing RpoB-YFP (yellow dots). Also shown are the pixel masks (white lines) obtained by automatic segmentation of the cell borders, using the method described in Supplementary Section III. Scale bars correspond to 1 µm. Related with Section '*The engagement between gyrases and nucleoid increases during cold shock*' of main manuscript.

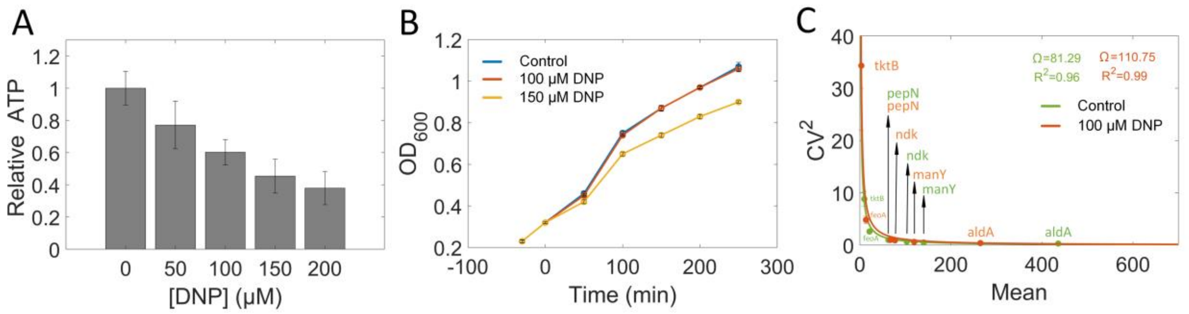

Figure S29: Cold shock repressed genes' response when subjected to 2,4-Dinitrophenol (DNP) **(A)** Relative changes in ATP levels (Supplementary Section XX), 120 min after subjecting cells to DNP. **(B)** Growth curves in optimal conditions following addition of DNP (0 min). **(C)** Squared coefficient of variation (CV^2) as a function of the mean protein numbers of 6 cold shock repressed genes (same genes as in Figure 4B). Related with Section '*Cellular energy levels decrease during cold shock*' of main manuscript.

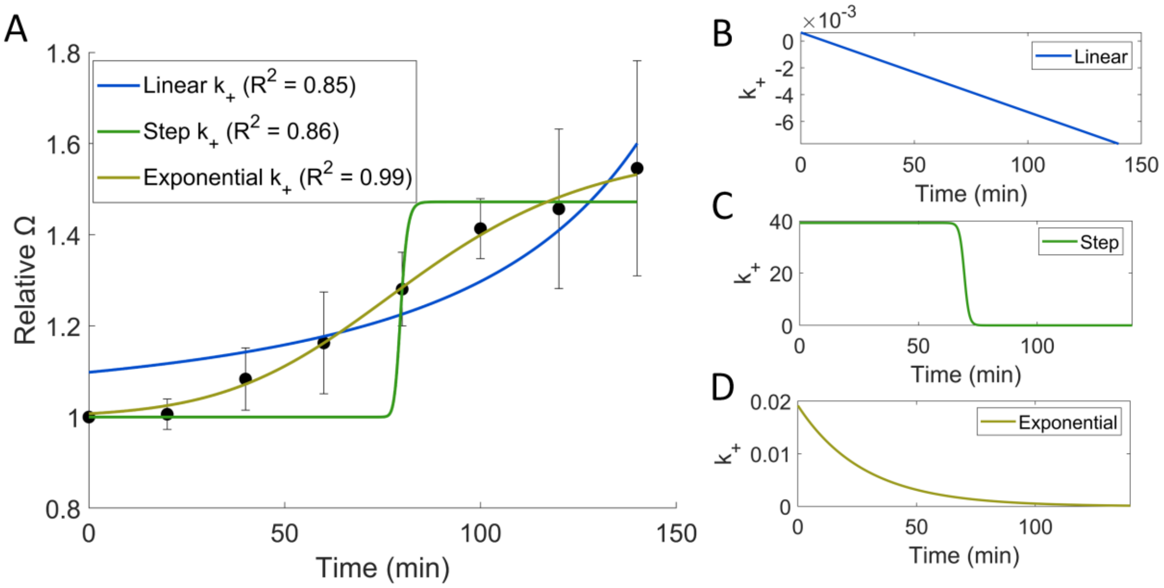

Figure S30: Relative Ω as a function of OFF-ON rates **(A)** Model fitting given a specific function for k_+ changes (linear, step and exponential function). Black dots are the empirical data for cold shock temperatures and error bars are the standard error. **(B-D)** Changes in k_+ over time assuming a linear, step and exponential function, respectively. In **A**-**D**, all lines are best fitting curves for respective model predictions. Supplementary Table S13 shows the R^2 for each model fit and prediction. Due to low R^2 when fitting the empirical data the fitting of quadratic type function is not shown. Related with Section '*Relative Ω as a function of OFF-ON rates in cold shock repressed genes*' of main manuscript.

Supplementary Tables

Table S1: Abbreviations and symbols used in this study.

Abbreviations	Full name
CS	Cold shock
CSR	Cold shock repressed
CV^2	Squared coefficient of variation
EF	Equal function
FDR	False Discovery Rate
GR	Global regulator
LFC	Log_2 fold changes
LFC_{CTRL}	Log_2 fold changes of control condition
LFC_{CS}	Log_2 fold changes of cold shock condition
LFC_{Novo}	Log_2 fold changes of novobiocin condition
M	Mean number of proteins
NAPs	Nucleoid-associated proteins
GO	Gene ontology
OLS	Ordinary Least Squares
RNAP	RNA polymerase
SD	Shine-Dalgarno sequence
SEM	Standard error of the mean
SKEW.	Skewness
SS	Supercoiling sensitive
STD	Standard deviation
TF	Transcription factor
TPM	Transcripts per million
Ω	Signal-to-noise ratio ($\Omega = CV^2 \times M$)
Γ	gamma function

Table S2: List of YFP-fusion strains (35) used in this study.

No.	Strain name	Genotype	Source
1	MG1655	λ-, rph-1	Yale CGSC (CGSC # 6300)
2	MGmCherry	Same as MG1655, with rpoS::mCherry, chromosomally integrated, replacing rpoS	Gift from James Locke (53)
3	SX1047	F-, Δ(argF-lac)169, gal-490, Δ(modF-ybhJ)803, λ[cI857 Δ(cro-bioA)], gyrA791-YFP(::cat), IN(rrnD-rrnE)1, rph-1	Yale CGSC (CGSC # 12602)
4	SX1056	F-, Δ(argF-lac)169, gal-490, Δ(modF-ybhJ)803, λ[cI857 Δ(cro-bioA)], IN(rrnD-rrnE)1, rph-1, gyrB792-YFP(::cat)	Yale CGSC (CGSC # 12611)
5	SX1020	F-, Δ(argF-lac)169, gal-490, Δ(modF-ybhJ)803, λ[cI857 Δ(cro-bioA)], topA791-YFP(::cat), IN(rrnD-rrnE)1, rph-1	Yale CGSC (CGSC # 12575)
6	SX1440	F-, Δ(argF-lac)169, gal-490, Δ(modF-ybhJ)803, λ[cI857 Δ(cro-bioA)], topB792-YFP(::cat), IN(rrnD-rrnE)1, rph-1	Yale CGSC (CGSC # 12995)
7	SX1051	F-, Δ(argF-lac)169, gal-490, Δ(modF-ybhJ)803, λ[cI857 Δ(cro-bioA)], IN(rrnD-rrnE)1, rph-1, rpoB791-YFP(::cat)	Yale CGSC (CGSC # 12606)
8	SX1397	F-, Δ(argF-lac)169, gal-490, Δ(modF-ybhJ)803, λ[cI857 Δ(cro-bioA)], clpA791-YFP(::cat), IN(rrnD-rrnE)1, rph-1	Yale CGSC (CGSC # 12952)
9	SX1519	F-, Δ(argF-lac)169, gal-490, Δ(modF-ybhJ)803, λ[cI857 Δ(cro-bioA)], pepN794-YFP(::cat), IN(rrnD-rrnE)1, rph-1	Yale CGSC (CGSC # 13074)
10	SX1812	F-, yaeH791-YFP(::cat), Δ(argF-lac)169, gal-490, Δ(modF-ybhJ)803, λ[cI857 Δ(cro-bioA)], IN(rrnD-rrnE)1, rph-1	Yale CGSC (CGSC # 13367)
11	SX1986	F-, Δ(argF-lac)169, gal-490, Δ(modF-ybhJ)803, λ[cI857 Δ(cro-bioA)], ydfG791-YFP(::cat), IN(rrnD-rrnE)1, rph-1	Yale CGSC (CGSC # 13541)
12	SX1882	F-, Δ(argF-lac)169, gal-490, Δ(modF-ybhJ)803, λ[cI857 Δ(cro-bioA)], yqjD791-YFP(::cat), IN(rrnD-rrnE)1, rph-1	Yale CGSC (CGSC # 13437)
13	SX1394	F-, Δ(argF-lac)169, gal-490, Δ(modF-ybhJ)803, λ[cI857 Δ(cro-bioA)], ndk-791-YFP(::cat), IN(rrnD-rrnE)1, rph-1	Yale CGSC (CGSC # 12949)
14	SX1989	F-, Δ(argF-lac)169, gal-490, Δ(modF-ybhJ)803, λ[cI857 Δ(cro-bioA)], yeeX793-YFP(::cat), IN(rrnD-rrnE)1, rph-1	Yale CGSC (CGSC # 13544)
15	SX1505	F-, Δ(argF-lac)169, gal-490, Δ(modF-ybhJ)803, λ[cI857 Δ(cro-bioA)], yciI793-YFP(::cat), IN(rrnD-rrnE)1, rph-1	Yale CGSC (CGSC # 13060)
16	SX1695	F-, Δ(argF-lac)169, gal-490, Δ(modF-ybhJ)803, λ[cI857 Δ(cro-bioA)], elaB792-YFP(::cat), IN(rrnD-rrnE)1, rph-1	Yale CGSC (CGSC # 13250)
17	SX1950	F-, Δ(argF-lac)169, gal-490, Δ(modF-ybhJ)803, λ[cI857 Δ(cro-bioA)], putP792-YFP(::cat), IN(rrnD-rrnE)1, rph-1	Yale CGSC (CGSC # 13505)
18	SX1550	F-, Δ(argF-lac)169, gal-490, Δ(modF-ybhJ)803, λ[cI857 Δ(cro-bioA)], IN(rrnD-rrnE)1, glpD792-YFP(::cat), rph-1	Yale CGSC (CGSC # 13105)
19	SX1674	F-, Δ(argF-lac)169, gal-490, Δ(modF-ybhJ)803, λ[cI857 Δ(cro-bioA)], gcvT792-YFP(::cat), IN(rrnD-rrnE)1, rph-1	Yale CGSC (CGSC # 13229)
20	SX1919	F-, Δ(argF-lac)169, gal-490, Δ(modF-ybhJ)803, λ[cI857 Δ(cro-bioA)], gcvP791-YFP(::cat), IN(rrnD-rrnE)1, rph-1	Yale CGSC (CGSC # 13474)
21	SX1917	F-, Δ(argF-lac)169, gal-490, Δ(modF-ybhJ)803, λ[cI857 Δ(cro-bioA)], gabD791-YFP(::cat), IN(rrnD-rrnE)1, rph-1	Yale CGSC (CGSC # 13472)
22	SX1901	F-, Δ(argF-lac)169, gal-490, Δ(modF-ybhJ)803, λ[cI857 Δ(cro-bioA)], aldA791-YFP(::cat), IN(rrnD-rrnE)1, rph-1	Yale CGSC (CGSC # 13456)
23	SX1488	F-, Δ(argF-lac)169, gal-490, Δ(modF-ybhJ)803, λ[cI857 Δ(cro-bioA)], IN(rrnD-rrnE)1, rph-1, tnaA791-YFP(::cat)	Yale CGSC (CGSC # 13043)
24	SX1284	F-, Δ(argF-lac)169, gal-490, Δ(modF-ybhJ)803, λ[cI857 Δ(cro-bioA)], gatZ794-YFP(::cat), IN(rrnD-rrnE)1, rph-1	Yale CGSC (CGSC # 12839)
25	SX1763	F-, Δ(argF-lac)169, gal-490, Δ(modF-ybhJ)803, λ[cI857 Δ(cro-bioA)], manY793-YFP(::cat), IN(rrnD-rrnE)1, rph-1	Yale CGSC (CGSC # 13318)
26	SX1416	F-, Δ(argF-lac)169, gal-490, Δ(modF-ybhJ)803, λ[cI857 Δ(cro-bioA)], pgk-791-YFP(::cat), IN(rrnD-rrnE)1, rph-1	Yale CGSC (CGSC # 12971)
27	SX1087	F-, Δ(argF-lac)169, bolA791-YFP(::cat), gal-490, Δ(modF-ybhJ)803, λ[cI857 Δ(cro-bioA)], IN(rrnD-rrnE)1, rph-1	Yale CGSC (CGSC # 12642)

28	SX1526	F-, Δ(argF-lac)169, gal-490, Δ(modF-ybhJ)803, λ[cI857 Δ(cro-bioA)], katE791-YFP(::cat), IN(rrnD-rrnE)1, rph-1	Yale CGSC (CGSC # 13081)
29	SX1771	F-, Δ(argF-lac)169, gal-490, Δ(modF-ybhJ)803, λ[cI857 Δ(cro-bioA)], nuoE794-YFP(::cat), IN(rrnD-rrnE)1, rph-1	Yale CGSC (CGSC # 13326)
30	SX1954	F-, Δ(argF-lac)169, gal-490, Δ(modF-ybhJ)803, λ[cI857 Δ(cro-bioA)], tktB792-YFP(::cat), IN(rrnD-rrnE)1, rph-1	Yale CGSC (CGSC # 13509)
31	SX1859	F-, Δ(argF-lac)169, gal-490, Δ(modF-ybhJ)803, λ[cI857 Δ(cro-bioA)], IN(rrnD-rrnE)1, rph-1, yjbQ792-YFP(::cat)	Yale CGSC (CGSC # 13414)
32	SX1781	F-, Δ(argF-lac)169, gal-490, Δ(modF-ybhJ)803, λ[cI857 Δ(cro-bioA)], IN(rrnD-rrnE)1, feoA791-YFP(::cat), rph-1	Yale CGSC (CGSC # 13336)
33	SX1718	F-, Δ(argF-lac)169, gal-490, Δ(modF-ybhJ)803, λ[cI857 Δ(cro-bioA)], wrbA791-YFP(::cat), IN(rrnD-rrnE)1, rph-1	Yale CGSC (CGSC # 13273)
34	SX1975	F-, Δ(argF-lac)169, gal-490, Δ(modF-ybhJ)803, λ[cI857 Δ(cro-bioA)], yccJ791-YFP(::cat), IN(rrnD-rrnE)1, rph-1	Yale CGSC (CGSC # 13530)
35	SX1085	F-, Δ(argF-lac)169, gal-490, Δ(modF-ybhJ)803, λ[cI857 Δ(cro-bioA)], IN(rrnD-rrnE)1, rph-1, tpiA791-YFP(::cat)	Yale CGSC (CGSC # 12640)
36	SX1349	F-, Δ(argF-lac)169, gal-490, Δ(modF-ybhJ)803, λ[cI857 Δ(cro-bioA)], IN(rrnD-rrnE)1, rph-1, pfkA791-YFP(::cat)	Yale CGSC (CGSC # 12904)
37	BW25993	pRSET B_QUEEN-2m_AMPR	Gift from Hiromi Imamura (47)

Table S3: Set of parameter values use to investigate the noise levels of each model: one rate-limiting step model, two rate-limiting step model and ON-OFF model (Figure 6A). Related with Section '*An ON-OFF model can explain the short-term dynamics of cold shock repressed genes*' of main manuscript.

Rate Constant (s^{-1})	One rate-limiting step model	Two rate-limiting step model	ON-OFF model
k_1	0.002	0.004	0.006
k_+			0.002
k_-			0.003
k_i		0.004	
k_2	0.230	0.230	0.230
λ_1	0.004	0.004	0.004
λ_2	2.9×10^{-5}	2.9×10^{-5}	2.9×10^{-5}

Table S4: List of global TF regulators (GRs) known to regulate 30 or more genes each. Related with Section '*Short-term responses of cold shock repressed genes cannot be explained by transcription factor interactions, AT richness, or closely spaced promoters*' of the manuscript and Supplementary Figure S9.

No.	GR	No. Genes regulated	Genes that code the GR
1	CRP	574	crp
2	FNR	310	fnr
3	IHF	258	ihfA; ihfB
4	Fis	239	fis
5	H-NS	194	hns
6	ArcA	184	arcA
7	NarL	138	narL
8	Fur	131	fur
9	Lrp	110	lrp
10	NsrR	84	nsrR
11	Cra	82	cra
12	FlhDC	82	flhD; flhC
13	CpxR	71	cpxR
14	NarP	66	narP
15	PhoB	66	phoB
16	LexA	61	lexA
17	PhoP	59	phoP
18	NtrC	56	glnG
19	MarA	46	marA
20	ModE	46	modE
21	SoxS	42	soxS
22	PdhR	41	pdhR
23	NagC	39	nagC
24	ArgR	38	argR
25	OxyR	35	oxyR
26	SlyA	34	slyA
27	IscR	32	iscR
28	CysB	31	cysB
29	PurR	31	purR
30	FhlA	30	fhlA

Table S5: Over-represented biological process according to the Gene Ontology (GO) Overrepresentation Test (Supplementary Section XVI) with Fisher's exact test with FDR correction. For each GO biological process, it is shown the number of genes related to it in the *E. coli* genome (out of the 4390 genes); the number of genes related in the cold shock repressed (CSR) cohort (out of the 376 recognized genes by GO); the number of genes expected to be present in a cohort of the size of the CSR cohort; and finally, the fold-enrichment. Related with Section '*Ontology and evolutionary fitness of short-term cold shock repressed genes*' of main manuscript and Supplementary Section IV.

Biological Processes	GO biological process complete	No. Genes in *E. coli* genome	No. Genes in CSR cohort	Expected No. Genes in CSR cohort	Fold Enrichment
Metabolic process (GO: 0008152**)**	Glycerol metabolic process (GO:0006071)	20	12	1.71	7.01
	Nitrogen cycle metabolic process (GO:0071941)	25	13	2.14	6.07
	Organic hydroxy compound catabolic process (GO:1901616)	52	16	4.45	3.59
	Generation of precursor metabolites and energy (GO:0006091)	210	60	17.99	3.34
	Organic acid catabolic process (GO:0016054)	191	35	16.36	2.14
	Carbohydrate metabolic process (GO:0005975)	389	61	33.32	1.83
	Organic acid metabolic process (GO:0006082)	542	77	46.42	1.66
Response to stimulus (GO: 0050896)	Response to stress (GO:0006950)	556	74	47.62	1.55
	Cellular response to toxic substance (GO:0097237)	28	10	2.4	4.17

Table S6: For each condition we did an ANCOVA test under the null hypothesis that the light blue and dark blue line are not statistically distinguishable. P-values are presented below. For conditions that did not reject the null hypothesis (p-values > 0.05). Related with Supplementary Figure S8.

Cold shock			Optimal
	80 min	180 min	80 min
20 min	0.15	0.10	0.52

Table S7: For each condition we did an ANCOVA test to test under the null hypothesis that the lines are not statistically distinguishable. P-values are presented below. For conditions that did not reject the null hypothesis (p-values > 0.05). Related with Supplementary Figure S9 (cold shock) and S25 (novobiocin).

Cold shock		
	80 min	180 min
20 min	0.36	0.18
Novobiocin		
All genes vs inputs	0.12	
Strongly SS vs Inputs	0.93	
Strongly SS vs GRs	0.72	

Table S8: For each condition we did an ANCOVA test under the null hypothesis that the grey and black line are not statistically distinguishable. P-values are presented below. For conditions that did not reject the null hypothesis (p-values > 0.05). Related with Supplementary Figure S8

	Cold shock		Optimal
	80 min	180 min	80 min
20 min	0.03	0.01	2.1×10^{-4}

Table S9: Equations of squared coefficient of variation (CV²) and skewness (*S*) as a function of Ω and the mean protein numbers (*M*). Also present is the derivation of CV² and *S* as a function of the model parameters. Step-by-step derivation is shown in Supplementary Section XIV.

Variable	Function of Ω and M	1-step model	2-step model	ON-OFF model
CV²	$CV^2 = \Omega \cdot \frac{1}{M}$	$CV^2 = \frac{1}{\frac{k_1 \cdot k_2}{\lambda_1 \cdot \lambda_2}} \cdot \left(1 + \frac{k_2}{\lambda_1 + \lambda_2}\right)$	$CV^2 = \frac{1}{\frac{\left(\frac{1}{k1} + \frac{1}{ki}\right)^{-1} \cdot k_2}{\lambda_1 \cdot \lambda_2}} \cdot \left(1 + \frac{k_2}{\lambda_1 + \lambda_2}\right)$	$CV^2 = \frac{1}{\frac{k_+}{k_+ + k_-} \cdot \frac{k_1 \cdot k_2}{y_1 \cdot y_2}} \cdot \left(1 + \frac{k_2}{y_1 + y_2}\left(1 + (1-P)\frac{k_1(k_0 + y_1 + y_2)}{(k_0 + y_1)(k_0 + y_2)}\right)\right)$
S	$S = \frac{2}{\sqrt{M}} \cdot \sqrt{\Omega}$	$S = \frac{2 \cdot \sqrt{\left(1 + \frac{k_2}{\lambda_1 + \lambda_2}\right)}}{\sqrt{\frac{k_1 \cdot k_2}{\lambda_1 \cdot \lambda_2}}}$	$S = \frac{2 \cdot \sqrt{\left(1 + \frac{k_2}{\lambda_1 + \lambda_2}\right)}}{\sqrt{\frac{\left(\frac{1}{k1} + \frac{1}{ki}\right)^{-1} \cdot k_2}{\lambda_1 \cdot \lambda_2}}}$	$S = \frac{2 \cdot \sqrt{\left(1 + \frac{k_2}{y_1 + y_2}\left(1 + (1-P)\frac{k_1(k_0 + y_1 + y_2)}{(k_0 + y_1)(k_0 + y_2)}\right)\right)}}{\sqrt{\frac{k_+}{k_+ + k_-} \cdot \frac{k_1 \cdot k_2}{y_1 \cdot y_2}}}$

Table S10: P-distances to the consensus Shine-Dalgarno (SD) sequence (AGGAGG) (30). The sequences were extracted from the available ones in RegulonDB. Shown are the mean and standard deviation (STD) of the p-distances of all genes (179 genes), of the 31 genes of those 179 that were found to cold shock repressed (CSR), and of 1000 cohorts of 31 genes assembled by random selection from the 179 genes. Related with Section '*An ON-OFF model can explain the short-term dynamics of cold shock repressed genes*' of main manuscript and Supplementary Sections IX, X and XI.

	Consensus SD sequence ('AGGAGG') (Mean ± STD)
Genome-wide p-distance (179 genes) ± STD	0.57 ± 0.22
p-distance of CSR genes (31 genes) ± STD	0.48 ± 0.20
p-distance of randomly selected genes (31 genes)	0.54 ± 0.20

Table S11: P-distances of the start codon sequences of cold shock repressed (CSR) genes to the most frequent start codon sequences (ATG; GTG; TTG; ATT; CTG) (31–33). Sequences extracted from RegulonDB. Shown are the mean and standard deviation (STD) of the p-distances of all genes (179 genes), of the 31 genes of those 179 that were found to CSR, and of 1000 cohorts of 31 genes assembled by random selection from the 179 genes. Related with Section '*An ON-OFF model can explain the short-term dynamics of cold shock repressed genes*' of main manuscript and Supplementary Sections IX, X and XI.

	ATG	GTG	TTG	ATT	CTG
Average start codon sequence (N = 179) ± STD *(RegulonDB)*	0.49 ± 0.22	0.64 ± 0.34	0.61 ± 0.29	0.47 ± 0.18	0.63 ± 0.31
Average start codon sequence of CSR genes (N = 31) ± STD	0.59 ± 0.47	0.71 ± 0.34	0.66 ± 0.29	0.52 ± 0.17	0.67 ± 0.30
Average start codon sequence of random cohort (31) ± STD	0.53 ± 0.49	0.63 ± 0.35	0.59 ± 0.28	0.52 ± 0.17	0.63 ± 0.31

Table S12: Reference parameter values assuming the one-step and the ON-OFF models (Figure 6A). Given that Lac is considered to be a strong promoter, combining these parameter values should result in relatively high RNA and protein numbers. Related with Section '*Relative Ω as a function of OFF-ON rates in cold shock repressed genes*' of main manuscript.

Rate Constant	Description	Values (s^{-1})	References
k_+	Promoter unlocking	7×10^{-4}	For the native Lac promoter (1, 54)
k_-	Promoter locking	0.0012	For the native Lac promoter (54, 55)
k_1	Transcription rate	8.3×10^{-4} to 0.02	Average expressing native genes (56)
k_2	Translation rate	0.3	Estimated from empirical data and (8)
λ_1	RNA degradation	0.004	Average RNA degradation rates (36)
λ_2	Protein decay (degradation and dilution in cell division)	2.93×10^{-5}	Average protein degradation rates (35, 57)

Table S13: Best fitting parameter values for model functions of k_+ as a function of temperature. Related with Section '*Relative Ω as a function of OFF-ON rates in cold shock repressed genes*' of main manuscript.

Function type	Equation	Estimated parameters	Average R^2
Linear	$b_2 \cdot x + b_1$	$b_1 = 6\times10^{-4}$; $b_2 = -5.9\times10^{-5}$; $k_- = 0.013\ s^{-1}$	0.85
Exponential	$e^{(-b\cdot x)} \cdot a$	b = 0.036; a = 0.019; $k_- = 0.002\ s^{-1}$	0.99
Quadratic	$b_3 \cdot x^2 + b_2 \cdot x + b_1$	$b_1 = 0.847$; $b_2 = 0.894$; $b_3 = -0.024$; $k_- = 1.183\ s^{-1}$	< 0
Step	$\left(1-\frac{1}{1+e^{-(x-L)}}\right)\times a$	a = 39.25; L = 69.32; $k_- = 0.003\ s^{-1}$	0.86

REFERENCES

1. Chong,S., Chen,C., Ge,H. and Xie,X.S. (2014) Mechanism of transcriptional bursting in bacteria. *Cell*, **158**, 314–326.
2. Bolger,A.M., Lohse,M. and Usadel,B. (2014) Trimmomatic: a flexible trimmer for Illumina sequence data. *Bioinformatics*, **30**, 2114.
3. Dobin,A., Davis,C.A., Schlesinger,F., Drenkow,J., Zaleski,C., Jha,S., Batut,P., Chaisson,M. and Gingeras,T.R. (2013) STAR: ultrafast universal RNA-seq aligner. *Bioinformatics*, **29**, 15–21.
4. Langmead,B. and Salzberg,S.L. (2012) Fast gapped-read alignment with Bowtie 2. *Nat. Methods*, **9**, 357–359.
5. Liao,Y., Smyth,G.K. and Shi,W. (2019) The R package Rsubread is easier, faster, cheaper and better for alignment and quantification of RNA sequencing reads. *Nucleic Acids Res.*, **47**, e47.
6. Love,M.I., Huber,W. and Anders,S. (2014) Moderated estimation of fold change and dispersion for RNA-seq data with DESeq2. *Genome Biol.*, **15**, 550.
7. Li,B. and Dewey,C.N. (2011) RSEM: accurate transcript quantification from RNA-Seq data with or without a reference genome. *BMC Bioinformatics*, **12**, 323.
8. Bar-even,A., Paulsson,J., Maheshri,N., Carmi,M., Shea,E.O., Pilpel,Y. and Barkai,N. (2006) Noise in protein expression scales with natural protein abundance. *Nature Genet*, **38**, 636–643.
9. Häkkinen,A., Muthukrishnan,A.-B., Mora,A., Fonseca,J.M. and Ribeiro,A.S. (2013) CellAging: a tool to study segregation and partitioning in division in cell lineages of Escherichia coli. *Bioinformatics*, **29**, 1708–1709.
10. Martins,L., Neeli-Venkata,R., Oliveira,S.M.D., Häkkinen,A., Ribeiro,A.S. and Fonseca,J.M. (2018) SCIP: a single-cell image processor toolbox. *Bioinformatics*, **34**, 4318–4320.
11. Mora,A.D., Vieira,P.M., Manivannan,A. and Fonseca,J.M. (2011) Automated drusen detection in retinal images using analytical modelling algorithms. *Biomed. Eng. Online*, **10**, 59.
12. Stracy,M., Schweizer,J., Sherratt,D.J., Kapanidis,A.N., Uphoff,S. and Lesterlin,C. (2021) Transient non-specific DNA binding dominates the target search of bacterial DNA-binding proteins. *Mol. Cell*, **81**, 1499-1514.e6.
13. Ashburner,M., Ball,C.A., Blake,J.A., Botstein,D., Butler,H., Cherry,J.M., Davis,A.P., Dolinski,K., Dwight,S.S., Eppig,J.T., *et al.* (2000) Gene Ontology: tool for the unification of biology. *Nat. Genet.*, **25**, 25–29.
14. Gene Ontology Consortium (2021) The Gene Ontology resource: enriching a GOld mine. *Nucleic Acids Res.*, **49**, D325–D334.
15. Mi,H., Muruganujan,A., Ebert,D., Huang,X. and Thomas,P.D. (2019) PANTHER version 14: more genomes, a new PANTHER GO-slim and improvements in enrichment analysis tools. *Nucleic Acids Res.*, **47**, D419–D426.
16. Benjamini,Y. and Hochberg,Y. (1995) Controlling the False Discovery Rate: A Practical and Powerful Approach to Multiple Testing. *J. R. Stat. Soc. Series B Stat. Methodol.*, **57**, 289–300.
17. Xavier,J.C., Gerhards,R.E., Wimmer,J.L.E., Brueckner,J., Tria,F.D.K. and Martin,W.F. (2021) The metabolic network of the last bacterial common ancestor. *Commun Biol*, **4**, 413.
18. Winter,D. (2017) rentrez: An R package for the NCBI eUtils API. *R J.*, **9**, 520.
19. Santos-Zavaleta,A., Salgado,H., Gama-Castro,S., Sánchez-Pérez,M., Gómez-Romero,L., Ledezma-Tejeida,D., García-Sotelo,J.S., Alquicira-Hernández,K., Muñiz-Rascado,L.J., Peña-Loredo,P., *et al.* (2018) RegulonDB v 10.5: tackling challenges to unify classic and high throughput knowledge of gene regulation in E. coli K-12. *Nucleic Acids Res.*, **47**, D212–D220.
20. deHaseth,P.L., Zupancic,M.L. and Record,M.T.,Jr (1998) RNA polymerase-promoter interactions: the comings and goings of RNA polymerase. *J. Bacteriol.*, **180**, 3019–3025.
21. Bahrudeen,M.N.M., Chauhan,V., Palma,C.S.D., Oliveira,S.M.D., Kandavalli,V.K. and Ribeiro,A.S. (2019) Estimating RNA numbers in single cells by RNA fluorescent tagging and flow cytometry. *J. Microbiol. Methods*, **166**.
22. Galbusera,L., Bellement-Theroue,G., Urchueguia,A., Julou,T. and van Nimwegen,E. (2020) Using fluorescence flow cytometry data for single-cell gene expression analysis in bacteria. *PLoS One*, **15**, e0240233.
23. Dahan,O., Gingold,H. and Pilpel,Y. (2011) Regulatory mechanisms and networks couple the different phases of gene expression. *Trends Genet.*, **27**, 316–322.
24. Yanofsky,C. (1981) Attenuation in the control of expression of bacterial operons. *Nature*, **289**, 751–758.

25. Proshkin,S., Rahmouni,A.R., Mironov,A. and Nudler,E. (2010) Cooperation between translating ribosomes and RNA polymerase in transcription elongation. *Science*, **328**, 504–508.
26. Alberts,B., Johnson,A., Lewis,J., Raff,M., Roberts,K. and Walter,P. (2008) Molecular Biology of the Cell 5th ed. Garland Science.
27. Crooks,G.E., Hon,G., Chandonia,J.-M. and Brenner,S.E. (2004) WebLogo: a sequence logo generator. *Genome Res.*, **14**, 1188–1190.
28. Schneider,T.D. and Stephens,R.M. (1990) Sequence logos: A new way to display consensus sequences. *Nucleic Acids Res.*, **18**, 6097–6100.
29. Ringquist,S., Shinedling,S., Barrick,D., Green,L., Binkley,J., Stormo,G.D. and Gold,L. (1992) Translation initiation in Escherichia coli: sequences within the ribosome-binding site. *Mol. Microbiol.*, **6**, 1219–1229.
30. Saito,K., Green,R. and Buskirk,A.R. (2020) Translational initiation in E. coli occurs at the correct sites genome-wide in the absence of mRNA-rRNA base-pairing. *Elife*, **9**, e55002.
31. Blattner,F.R., Plunkett,G., Bloch,C.A., Perna,N.T., Burland,V., Riley,M., Collado-Vides,J., Glasner,J.D., Rode,C.K., Mayhew,G.F., *et al.* (1997) The complete genome sequence of Escherichia coli K-12. *Science*, **277**, 1453–1462.
32. Sacerdot,C., Fayat,G., Dessen,P., Springer,M., Plumbridge,J.A., Grunberg-Manago,M. and Blanquet,S. (1982) Sequence of a 1.26-kb DNA fragment containing the structural gene for E.coli initiation factor IF3: presence of an AUU initiator codon. *EMBO J.*, **1**, 311–315.
33. Missiakas D, Georgopoulos C and Raina S (1993) The Escherichia coli heat shock gene htpY: mutational analysis, cloning, sequencing, and transcriptional regulation. *J. Bacteriol.*, **175**, 2613–2624.
34. Ng,W. (2019) Database of ribosome binding site of all genes in Escherichia coli K-12 MG1655. 10.6084/M9.FIGSHARE.10282685.V1.
35. Taniguchi,Y., Choi,P.J., Li,G.-W., Chen,H., Babu,M., Hearn,J., Emili,A. and Xie,X.S. (2010) Quantifying E. coli Proteome and Transcriptome with Single-Molecule Sensitivity in Single Cells. *Science*, **329**, 533–538.
36. Bernstein,J.A., Khodursky,A.B., Lin,P.-H., Lin-Chao,S. and Cohen,S.N. (2002) Global analysis of mRNA decay and abundance in Escherichia coli at single-gene resolution using two-color fluorescent DNA microarrays. *Proceedings of the National Academy of Sciences*, **99**, 9697 LP – 9702.
37. Selinger,D.W., Saxena,R.M., Cheung,K.J., Church,G.M. and Rosenow,C. (2003) Global RNA half-life analysis in Escherichia coli reveals positional patterns of transcript degradation. *Genome Res.*, **13**, 216–223.
38. Zaslaver,A., Bren,A., Ronen,M., Itzkovitz,S., Kikoin,I., Shavit,S., Liebermeister,W., Surette,M.G. and Alon,U. (2006) A comprehensive library of fluorescent transcriptional reporters for Escherichia coli. *Nat. Methods*, **3**, 623–628.
39. Leng,F., Chen,B. and Dunlap,D.D. (2011) Dividing a supercoiled DNA molecule into two independent topological domains. *Proc. Natl. Acad. Sci. U. S. A.*, **108**, 19973–19978.
40. Baptista,I.S.C. and Ribeiro,A.S. (2020) Stochastic models coupling gene expression and partitioning in cell division in Escherichia coli. *Biosystems.*, **193–194**.
41. Peter,B.J., Arsuaga,J., Breier,A.M., Khodursky,A.B., Brown,P.O. and Cozzarelli,N.R. (2004) Genomic transcriptional response to loss of chromosomal supercoiling in Escherichia coli. *Genome Biol.*, **5**, R87.
42. Yeung,E., Dy,A.J., Martin,K.B., Ng,A.H., Del Vecchio,D., Beck,J.L., Collins,J.J. and Murray,R.M. (2017) Biophysical Constraints Arising from Compositional Context in Synthetic Gene Networks. *Cell Syst*, **5**, 11-24.e12.
43. Zhang,Y.E., Bærentsen,R.L., Fuhrer,T., Sauer,U., Gerdes,K. and Brodersen,D.E. (2019) (p)ppGpp Regulates a Bacterial Nucleosidase by an Allosteric Two-Domain Switch. *Mol. Cell*, **74**, 1239-1249.e4.
44. Ohlsen,K.L. and Gralla,J.D. (1992) Interrelated effects of DNA supercoiling, ppGpp, and low salt on melting within the Escherichia coli ribosomal RNA rrnB P1 promoter. *Mol. Microbiol.*, **6**, 2243–2251.
45. Sanchez-Vazquez,P., Dewey,C.N., Kitten,N., Ross,W. and Gourse,R.L. (2019) Genome-wide effects on Escherichia coli transcription from ppGpp binding to its two sites on RNA polymerase. *Proc. Natl. Acad. Sci. U. S. A.*, **116**, 8310–8319.
46. McDonald,J.H. (2009) Handbook of biological statistics Baltimore, MD: sparky house publishing.

47. Yaginuma,H., Kawai,S., Tabata,K.V., Tomiyama,K., Kakizuka,A., Komatsuzaki,T., Noji,H. and Imamura,H. (2014) Diversity in ATP concentrations in a single bacterial cell population revealed by quantitative single-cell imaging. *Sci. Rep.*, **4**, 1–7.
48. Widdel,F. (2007) Theory and measurement of bacterial growth. *Di dalam Grundpraktikum Mikrobiologie*, **4**, 1–11.
49. Startceva,S., Kandavalli,V.K., Visa,A. and Ribeiro,A.S. (2019) Regulation of asymmetries in the kinetics and protein numbers of bacterial gene expression. *Biochimica et Biophysica Acta - Gene Regulatory Mechanisms*, **1862**, 119–128.
50. Newman,J.R.S., Ghaemmaghami,S., Ihmels,J., Breslow,D.K., Noble,M., DeRisi,J.L. and Weissman,J.S. (2006) Single-cell proteomic analysis of S. cerevisiae reveals the architecture of biological noise. *Nature*, **441**, 840–846.
51. Vogel,C. and Marcotte,E.M. (2012) Insights into the regulation of protein abundance from proteomic and transcriptomic analyses. *Nat. Rev. Genet.*, **13**, 227–232.
52. Liu,Y., Beyer,A. and Aebersold,R. (2016) On the Dependency of Cellular Protein Levels on mRNA Abundance. *Cell*, **165**, 535–550.
53. Patange,O., Schwall,C., Jones,M., Villava,C., Griffith,D.A., Phillips,A. and Locke,J.C.W. (2018) Escherichia coli can survive stress by noisy growth modulation. *Nat. Commun.*, **9**.
54. Palma,C.S.D., Kandavalli,V., Bahrudeen,M.N.M., Minoia,M., Chauhan,V., Dash,S. and Ribeiro,A.S. (2020) Dissecting the in vivo dynamics of transcription locking due to positive supercoiling buildup. *Biochimica et Biophysica Acta (BBA) - Gene Regulatory Mechanisms*, **1863**, 194515.
55. Stracy,M., Wollman,A.J.M., Kaja,E., Gapinski,J., Lee,J.-E., Leek,V.A., McKie,S.J., Mitchenall,L.A., Maxwell,A., Sherratt,D.J., *et al.* (2019) Single-molecule imaging of DNA gyrase activity in living Escherichia coli. *Nucleic Acids Res.*, **47**, 210–220.
56. Prajapat,M.K. and Ribeiro,A.S. (2018) Added value of autoregulation and multi-step kinetics of transcription initiation. *Royal Society Open Science*, **5**, 14–19.
57. Koch,A.L. and Levy,H.R. (1955) Protein turnover in growing cultures of Escherichia coli. *J. Biol. Chem.*, **217**, 947–957.
